# Should Australia Ban the Use of Genetic Test Results in Life Insurance?

**DOI:** 10.3389/fpubh.2017.00330

**Published:** 2017-12-13

**Authors:** Jane Tiller, Margaret Otlowski, Paul Lacaze

**Affiliations:** ^1^Public Health Genomics, Department of Epidemiology and Preventive Medicine, School of Public Health and Preventive Medicine, Monash University, Melbourne, VIC, Australia; ^2^Centre for Law and Genetics, Faculty of Law, University of Tasmania, Hobart, TAS, Australia

**Keywords:** genetics, insurance, genetic discrimination, regulation, legislation, moratorium

## Abstract

Under current Australian regulation, life insurance companies can require applicants to disclose all genetic test results, including results from research or direct-to-consumer tests. Life insurers can then use this genetic information in underwriting and policy decisions for mutually rated products, including life, permanent disability, and total income protection insurance. Over the past decade, many countries have implemented moratoria or legislative bans on the use of genetic information by life insurers. The Australian government, by contrast, has not reviewed regulation since 2005 when it failed to ensure implementation of recommendations made by the Australian Law Reform Commission. In that time, the Australian life insurance industry has been left to self-regulate its use of genetic information. As a result, insurance fears in Australia now are leading to deterred uptake of genetic testing by at-risk individuals and deterred participation in medical research, both of which have been documented. As the potential for genomic medicine grows, public trust and engagement are critical for successful implementation. Concerns around life insurance may become a barrier to the development of genomic health care, research, and public health initiatives in Australia, and the issue should be publicly addressed. We argue a moratorium on the use of genetic information by life insurers should be enacted while appropriate longer term policy is determined and implemented.

Australia has a concerning lack of regulation around the use of genetic test results by the life insurance industry. Many other countries have passed legislation or moratoria banning use of genetic data in life insurance (1). However, in Australia, life insurance applicants must still disclose results of any genetic test if requested. These include findings from research or direct-to-consumer genetic testing, if known to the applicant. Genetic results can be used for underwriting life insurance, permanent disability and total income protection insurance in Australia, with minimal consumer transparency or Government oversight into the process.

Genetic test results cannot affect private health insurance premiums in Australia, which are community-rated under the *Private Health Insurance Act 2007* (Cth). This means private health insurance companies in Australia must offer the same premiums to all consumers for equivalent policies and cannot discriminate on the basis of health or other information.

Section 46 of the *Disability Discrimination Act 1992* (Cth) permits life insurers to discriminate on the basis of genetic test results, only where actuarially sound or otherwise reasonable. Yet cases of life insurance policies being declined or premiums loaded without adequate supporting data or justification have been documented in Australia over a number of years (2–4), despite the known difficulties in documenting such cases of discrimination (5, 6).

In one case, a woman with an identified BRCA gene change indicating elevated risk of breast cancer, elected to have a bilateral prophylactic mastectomy to reduce her risk. However, the risk reduction surgery was not taken into account by her life insurer and in her application for death and critical illness cover, the insurer excluded any cancer cover and imposed a 50% premium loading for death cover (7).

In another case, a man with a family history of colorectal cancer had an identified gene change confirming his increased risk. He actively sought increased surveillance through colonoscopies to reduce his risk back down to population average, yet was still refused cancer cover. The man eventually obtained cover, but only after taking a complaint to the Human Rights Commission (8).

These examples of genetic discrimination can occur because of the current lack of enforced regulation of the Australian life insurance industry. The issue is of increasing public health concern, with evidence of insurance fears now deterring the uptake of genetic testing and participation in medical research at a critical time for genomics in Australia.

The strongest evidence for deterred uptake of genetic testing due to insurance fears in Australia comes from within the context of Lynch syndrome (increased risk of hereditary colorectal cancer), whereby predictive gene testing can identify risk and prompt surveillance to prevent cancer. A Victorian study related to Lynch syndrome saw the number of individuals declining predictive gene testing more than double after insurance was mentioned on consent forms, compared with a similar time period without mention of insurance (9). Predictive genetic testing for Lynch syndrome can identify risk and prompt surveillance to prevent cancer, and so the deterrence of at-risk individuals is a significant public health concern. A qualitative study from the same group found insurance fears quoted as a leading reason for refusal of testing in Lynch syndrome families (10). Documented cases of deterrence from medical research participation by healthy volunteers are more difficult to identify, yet do exist in Australia (11).

We argue that this mounting evidence, in conjunction with the ethical and social imperatives, justifies a moratorium on the use of genetic data in life insurance in Australia, with the exception of negative (mutation-absent) test results, until appropriate long-term policy is implemented.

## International Action, Australian Inaction

Currently in Australia, genetics professionals commonly recommend clients organize life insurance policies before undertaking genetic testing. This practice, which is designed to protect clients from insurers refusing cover based on the results of future genetic tests, can also result in some individuals declining genetic testing altogether due to insurance fears (9, 10). For some individuals, declining predictive genetic testing can mean missing out on information that could prompt life-saving measures, such as surveillance and early intervention for serious but treatable conditions such as cancer.

Internationally, many countries have instituted bans on the use of genetic test results by life insurers (1). Two noteworthy examples are Canada and the UK. Canada passed the *Genetic Non-Discrimination Act* (previously Bill S-201) in May 2017, prohibiting insurers from requesting or requiring disclosure of any previous or future genetic test results. There is some controversy over whether the Act is a legitimate exercise of Federal power (12) and it has been referred to the Court of Appeal of Quebec for determination of a challenge of its Constitutionality (13). This challenge is unique to the division of power under Canada’s Constitution and would not apply in Australia.

Since 2001, a moratorium and concordat between the UK Government and the Association of British Insurers has been in place on the use of predictive genetic test results by life insurers (other than negative test results and results for Huntington’s Disease for policies above £500,000). This moratorium has been extended until 2019 (14).

Furthermore, the European Convention on Human Rights and Biomedicine and Recommendation CM/Rec(2016)8 direct Member States to take steps to prevent discrimination, including on grounds of genetic characteristics, in insurance contracts. A mix of legislative reforms and moratoria have been enacted as a result in many European countries (1).

By contrast, Australia has left its life insurance industry to self-regulate the use of genetic information, without independent regulatory oversight. The Australian Government has not reviewed regulation since 2005 when it made non-binding recommendations following the Australian Law Reform Commission report “Essentially Yours” (15, 16). Many of these recommendations, although commendable, unfortunately have not been implemented or adhered to by the Australian life insurance industry.

The Financial Services Council, the peak industry body in Australia for life insurers, writes the Industry Standard on Genetic Testing (17) which binds its members. The Standard now contains several clauses that could be considered to conflict with the 2005 Government recommendations, including a recently added clause requiring applicants to disclose to insurers even a *consideration* of genetic testing, if requested. It is uncertain how insurers will use an affirmative response, but we consider even the inclusion of this request to be evidence of an erosion in consumer rights made possible by lack of regulatory oversight (18). Any model of industry self-regulation for the use of genetic information by life insurers, who are inherently motivated by commercial gain, represents a conflict of interest. Independent government oversight is needed.

## Australian Parliamentary Inquiry into the Life Insurance Industry

In 2016, the Australian life insurance industry came under scrutiny by an Inquiry of the Parliamentary Joint Committee on Corporations and Financial Services in relation to a range of practices. Authors of this article, with input from others, presented our concerns regarding genetics and life insurance to the Committee in May 2017 making recommendations for an immediate moratorium on the use of genetic test results and a flexible legislative instrument for long-term regulation. The written submission and transcripts of the public hearings can be found online (19) (see Supplementary Material).

Any moratorium or ban on use of genetic test results for life insurance in Australia should consider the use of negative (mutation-absent) test results to counter family history. That is, individuals who undertake predictive gene testing for a known family variant but are found *not* to carry the family variant, thereby having their risk reduced compared with gene-positive family members, should have this information taken into account by insurers to counter increased risk indicated by family history of disease. Without introducing such measures, any regulation aimed at regulating insurer conduct and protecting consumers from insurance discrimination is likely to have unintended consequences. These include excluding individuals from being able to prove that family history does not lead to an increased individual risk, which would put them in a worse position than currently. This would benefit some consumers to the detriment of others, which is a poor public health outcome. For this reason, an exception for negative test results has been incorporated into the UK Moratorium and Concordat (14).

## Possible Implications of a Ban

The insurance industry claims that if genetic test results cannot be used in life insurance, adverse selection by gene-positive applicants will lead to significantly increased premiums for consumers and incapacitate the operation of insurance markets (17). However, there is little evidence produced in Australia to support this claim. A report prepared for the Actuaries Institute 2017 Summit (20) asserts that a ban on genetic test results will result in adverse selection, but its claims are arguably based on a set of worst-case assumptions that are unlikely to be met (18). Independent modeling undertaken elsewhere, including in Canada prior to legislation being passed, indicates that a ban on the use of genetic test results would not have a significant effect on the operation of a reasonably sized life insurance market (21–23).

Another argument is that genetic data should not be treated differently from other medical risk information. However, we argue that given the lack of underlying actuarial data currently available for genetics, the family implications of genetic test results, and other attendant ethical, legal and privacy issues, genetic data is different than and should be treated differently to other types of medical risk information.

We acknowledge the insurance industry must be commercially viable. However, the use of individuals’ genetic information has wide-ranging ethical and social implications which warrant curtailment of the industry’s use of this information. Over time, the self-regulating industry in Australia has changed its policy on genetic testing with relative freedom, meaning current requirements for disclosure of genetic test results could be further changed, without necessary government involvement or independent regulatory oversight. This poses a growing concern for consumers.

Our understanding of human genetic variation is still evolving, and the classification of most genetic variants is not yet supported by robust population data, certainly not to the level of being sufficient for insurance underwriting. Some of the first large-scale surveys of human genetic variation are only now underway (24), and we are still largely unaware of the true population frequency of most genetic risk variants. Social policy considerations, which include factors such as privacy, fairness, equality of access to insurance, non-deterrence and non-maleficence should also be carefully considered.

## Future for Australia

More than ever, now is a critical time for genomics and genomic research in Australia. The Commonwealth, Queensland, Victorian, and New South Wales Governments have each recently committed $25 million toward the implementation of genomics into health care, with new genomic technologies and whole-genome sequencing showing much promise. Consultation has been undertaken for a National Health Genomics Policy Framework, which aims to integrate genomics further into national health care. However, these steps are being taken without adequately addressing the issue of life insurance. As the lines between research and clinical care for genomics are blurred, Australia needs more education, consumer protection and building of public trust in genetics, not an environment of uncertainty, consumer fears and inadequate regulation. The Government must be more proactive, and take ownership of the issue within a specific department for closer oversight.

Insurance fears now represent a growing threat to public trust in genetics in Australia at a time when it is needed most. A failure to address this key issue will remain an ongoing barrier if action is not taken. The threat of genetic discrimination in Australia has been voiced for well over a decade without a satisfactory Government response. The Human Genetics Society of Australasia has for years called for both a moratorium and legislation banning use of predictive genetic test results by the insurance industry (25). We urge Australia to follow most developed nations and enact a moratorium, then pass legislation to safeguard its population. There is still time for Australia to proactively address this issue; however, the time to take action is now.

## Author Contributions

JT, MO, and PL conceived and wrote the manuscript jointly.

## Conflict of Interest Statement

The authors declare that the research was conducted in the absence of any commercial or financial relationships that could be construed as a potential conflict of interest.
