# Supplementary material for: Should Australia Ban the Use of Genetic Test Results in Life Insurance?
Source: Front Public Health. 2017 Dec 13;5:330. doi: 10.3389/fpubh.2017.00330 (PMC5733354; doi:10.3389/fpubh.2017.00330)
Supplement: Supplementary file 1 [file Presentation_1.PDF]

Committee Secretary
Parliamentary Joint Committee on Corporations and Financial Services
PO Box 6100
Parliament House
Canberra ACT 2600

By email: corporations.joint@aph.gov.au

Submission regarding Inquiry into the life insurance industry

Dear Sir/Madam

The Australian Genetic Non-Discrimination Working Group, comprised of genetic and medical professionals, research scientists, lawyers, genetic counselling, law and bioethics academics, social scientists and a senior actuary with experience in this area, encloses its submission to the current inquiry into the Australian Life Insurance Industry.

Our submission summarises the concerns of the Working Group regarding the use of genetic information by the life insurance industry. Please contact us if the Inquiry requires further information regarding any of the matters raised in this submission. We would be pleased to assist the Inquiry to understand this complex area if further information is required.

Yours Faithfully
Members of the Australian Genetic Non-Discrimination Working Group

- *Margaret Otlowski* (chair)
  Deputy Director, Centre for Law and Genetics, Faculty of Law, University of Tasmania
- *Ainsley Newson*
  Associate Professor of Bioethics, Centre for Values, Ethics and the Law in Medicine - University of Sydney
- *Alan Doble*
  Senior actuary and past convenor of an Institute of Actuaries of Australia genetics working group
- *Bruce Holloway*
  Emeritus Professor of Genetics and former Foundation Professor of Genetics, Monash University
- *Elly Lynch*
  Genetic Counsellor and Clinical Project Manager, Melbourne Genomics Health Alliance
- *Ingrid Winship*
  Executive Director of Research and Director of Genetic Medicine, Royal Melbourne Hospital
- *Jane Tiller*
  Lawyer and Master of Genetic Counselling student, University of Melbourne
- *Kristine Barlow-Stewart*
  Director, Master of Genetic Counselling Programme, University of Sydney
- *Louise Keogh*
  Health Sociologist and Associate Professor, Gender and Women's Health, University of Melbourne
- *Paul Lacaze*
  Head, Public Health Genomics Program, Monash University
- *Penny Gleeson*
  Lawyer and member of Victorian Genomic Advisory Committee

Australian Genetic Non-Discrimination Working Group
Submission to Inquiry into the Australian Life Insurance Industry

An Australian working group has recently formed to explore issues around the use of genetic information, including by the insurance industry. The group is comprised of genetic and medical professionals, research scientists, lawyers, genetic counselling, law and bioethics academics, social scientists and a senior actuary with experience in this area.

This submission primarily addresses subsection (a) of the Terms of Reference– the need for further reform and improved oversight of the life insurance industry. It also briefly addresses subsection (e) – the effectiveness of internal dispute resolution in life insurance.

SUMMARY

A. Australian government review of the use of genetic test result data by the life insurance industry is over 10 years old, and given advances in this field over this period, further review is required

B. Internationally, concern over the use of genetic test results has led other countries to adopt regulatory responses such as legislation or moratoriums to prohibit or discourage the use of certain genetic information by the insurance industry

C. New technologies and cost reductions are making genetic testing far more readily accessible than before – in particular, the increased accessibility of whole-genome sequencing (WGS) has resulted in a significant increase in the amount of genetic information available, with potential for wider implementation

D. In a research context, the availability of WGS is also leading to a dramatic increase in the amount of genetic information generated. Such findings are not usually validated through laboratories accredited by the National Association of Testing Authorities (NATA) for use in the clinical setting

E. Direct-to-consumer (DTC) testing at a decreasing cost is also leading to an increased amount of genetic test result data that is returned to consumers, and which is not necessarily validated through NATA accredited laboratories

F. The first population-scale surveys of human genetic variation are showing that the ability to predict the risk of many diseases through genetic information is even less statistically robust than previously believed

G. Australian life insurers should not be able to make underwriting decisions using genetic information in the absence of robust statistical data as per the exemption under the Disability Discrimination Act

H. Concerns around the insurance implications of the use of genetic information are affecting public willingness to participate in research as well as the uptake of potentially life-saving genetic testing, and Human Research Ethics Committees have been reticent to approve genetic research projects due to concerns about insurance implications

I. Life insurers are not required to transparently justify underwriting decisions made on the basis of genetic information, and there is no easily accessible appeals process for applicants

J. Australia must be proactive in regulating the use of genetic information to protect individuals, safeguard the future of genetic research and facilitate genetic testing in the health-care setting

RECOMMENDATIONS

1. The Commonwealth Department of Health (DOH) convene a roundtable meeting of genetic health and research experts, and patient and insurance industry representatives. At that meeting participants will agree on actions to ensure that genetic information is not used by the insurance industry in a manner that would undermine the current and potential public health benefits of genetic research and medicine. At that meeting, the recommendations discussed should include the remainder of the recommendations listed below.
2. Formation of a suitably qualified working group within the DOH which will determine which genetic tests or results can be used in underwriting decisions.
3. Development of standards to ensure that life insurance companies comply with the determinations of the working group.
4. Development of a clear and accessible avenues for the giving of reasons for adverse insurance decisions and appeals by applicants.
5. Exclusion of the use of genetic information generated by genetic tests conducted in research studies by life insurance companies in making underwriting decisions.
6. Development of a formal structure to govern regular audits of the use of genetic information by life insurance companies, in cooperation with the FSC.
7. One of the steps to be considered by the roundtable would be the implementation of a temporary moratorium on the use of genetic test results by life insurance companies, except in the case of using a negative (mutation free) predictive test result to counter family history, while appropriate and flexible policies are developed which can adapt to rapidly advancing technology and the increasing volume of genetic information.

DISCUSSION

Use of genetic information in underwriting decisions

International context

Internationally, there has been concern for some decades regarding the use of genetic information generated from genetic testing and the potential for discrimination against individuals on the basis of their genetic characteristics, particularly by insurance companies.

In the United Kingdom, a voluntary concordat and moratorium agreed between the government and the Association of British Insurers prevents life insurers from using genetic test result information in their policy decisions up to a certain limit, and only very limited information above that limit. However, recipients of ‘negative’ predictive test results can use this to counter an adverse family history.

The USA has introduced the Genetic Non-Discrimination Act (GINA). As health insurance is mutually rated in the USA, this is the focus of the Act – it does not apply to life insurance policies. However, some states in the USA have enacted separate legislation to regulate the use of genetic information by life insurance companies.

In Canada, Bill S-201 (passed by the Senate and now in the House of Commons) prohibits requesting genetic testing, asking for or disclosing genetic test results without consent. It also amends the Canadian Human Rights Act to add "genetic characteristics" as a type of discrimination.

The Council of Europe's Convention on Human Rights and Biomedicine prohibits genetic discrimination. Different countries in Europe have taken different approaches in terms of the implementation of this prohibition, but there is a general agreement that the use of genetic testing in private insurance should be prohibited or at least restricted. Many countries, including Belgium, Austria, Denmark, France, Germany, Lithuania, Norway, Portugal, and Sweden, have enacted legislation that prohibits the use of genetic information by insurers in setting premium levels.

Australia

In 2003, the Australian Law Reform Commission and the Australian Health Ethics Committee published the results of an inquiry into the use of genetic information, titled "Essentially Yours: The Protection of Human Genetic Information in Australia (ALRC Report 96)" (**the 2003 report**). The Inquiry stated that, "As a general rule, there should be no departure from the fundamental principle underlying the market in voluntary, mutually rated life insurance, namely equality of information between the applicant and the insurer. However, where the underwriting of insurance involves the use of human genetic information, the insurance process should be subject to the Recommendations in this Report." (Recommendation 26-1). That recommendation was made in the context of a number of other recommendations (set out in full in Appendix 1 of this submission) which were intended to lead to regulation regarding the use of genetic test results by insurers and protect individuals from discrimination in the use of that information.

Currently, Australian life insurance companies do not require applicants to undergo genetic testing before applying for life insurance policies, regardless of their family history. The working group considers that this practice is sound and should remain. However, once genetic tests have been undertaken, the use of that information by insurance companies is currently not well regulated. In the 2003 report, the Inquiry found that existing legal mechanisms and industry practices fell short of the desired standards in several respects. Their recommendations relate mainly to the review, by an expert body, of genetic tests for use by the insurance industry and measures to promote transparency by the industry.

Despite Commonwealth Government support for many of the 2003 report recommendations in its response, the (now disbanded) Human Genetics Advisory Committee (HGAC) did not establish procedures for assessing the use of genetic tests, and the Government left the implementation of many of the recommendations to the insurance industry, which has not taken steps to do so. Clearly this approach was not sufficient to ensure the level of protection which the Inquiry considered in 2003 should be afforded to individuals as a balance to the ability of insurers to use their genetic information. Thirteen years later, with the development of and rapidly decreasing cost of technologies such as next-generation sequencing technology, the volume of genetic data and the ease with which it can be obtained is vastly increased and the need for protection of individuals is critically higher. On this basis, the Government must take an approach that directly requires implementation by insurers if it is to be satisfied it is discharging its duty to protect individuals from misuse of genetic test result information. At the very least, the insurance industry should be required to implement those recommendations (or adopt measures consistent with those recommendations and appropriate to take into account the changes in technology and genetic science since 2003).

The working group appreciates that this topic is a significant one which requires expertise and consultation with a number of key stakeholders, including representatives from the insurance industry.

Life insurance code of conduct – October 2016

The working group is aware that the FSC began rolling out a Code of Conduct to its member life insurers with effect from 1 October 2016, and expects them to have completed their transition to the new Code by 30 June 2017. That Code indirectly addresses several of the points made in this submission, but it does not specifically mention genetic information. The working group agrees with the views presented in the 2003 report, which considered that genetic information presents a special case, requiring specific consideration. We consider that the Life Insurance Code of Conduct would need strengthening to address those specific concerns. Nevertheless, the advent of the Code shows a willingness by the life insurance industry to improve self-regulation. The Code may therefore be a good vehicle through which to introduce the reforms on the use of genetic information advocated in this submission.

Influence of insurance implications on uptake of genetic testing

Australian and international research has shown that a fear of adverse determinations by life insurers acts as a deterrent to obtaining life-changing or possibly life-saving genetic testing. In one study, the number of people who declined predictive testing when informed of the insurance implications was more than double the number who declined without knowledge of the insurance implications[1].This obviously has both health and cost implications both for individuals and the public health system. The decision not to have testing for fear of insurance implications may mean that the public health system will bear the burden of care for individuals who choose not to undergo genetic testing for predisposition to preventable conditions.

Genetic information with uncertain significance and research findings

Historically, genetic studies have focused on families affected by disease. Recently, genetic research in healthy population genetics has shown that many genetic changes previously thought to be disease-causing are in fact benign and commonly found in the healthy population. In addition, many gene changes (mutations) can cause disease in some people but not in others. This is known as incomplete penetrance, and it can create issues for the calculation of risk on the basis of genetic testing, especially given the volume and complexity of data that is being generated through the development of new technology. Sufficient population data (including data from individuals with a range of ethnicities) has not yet been generated to allow for robust statistical analysis of many apparently disease-causing genetic changes.

The Inquiry's findings in the 2003 report did not distinguish between genetic test results showing the basis for disease (diagnostic testing), and predictive genetic testing showing pre-symptomatic potential (but not certainty) for disease development in future. As technology advances, its ability to detect genetic changes that are not fully understood by researchers grows exponentially. The statistical value of these results is often low, but current policy does not regulate of the use of this information by insurers through determination by an independent body. The working group is concerned that the lack of regulation could allow insurance companies to make policy decisions based on poorly understood genetic data. This is particularly pertinent in a research setting, where research participants may have genetic results returned to them outside of a clinical context.

---

[1] L. Keogh et al. (2009)

Currently, the FSC Standard No 11 (Genetic Testing Policy) does not distinguish between genetic information received through participation in research and in a clinical setting. This means that, if requested, applicants for life insurance are required to disclose any genetic data that has been returned to them through participation in a research study, even if that data has not been validated by an appropriately accredited laboratory to enable it t be used clinically, and even if the significance of that data is uncertain. Such data is not appropriate for use by insurance companies in making underwriting decisions, but there is currently no independent oversight over how insurance companies may use this data, whether insurance companies' advisors have sufficient expertise to understand and interpret complex genetic information or whether their underwriting decisions are made with a sufficient basis.

Further, it has been shown that fear of insurance implications also affects the public's willingness to participate in research, which will detrimentally affect future genetic research. Human Research Ethics Committees have also been reticent to approve genetic research studies without amendment based on insurance concerns. For example, one study that was to provide a risk estimate regarding risk of melanoma using genetic analysis initially had approval withheld unless the researchers stipulated in the participant information sheet that they should seek independent legal advice prior to participating. The researchers successfully appealed this determination[2], but it is clear that if such requirements become commonplace, the willingness of research participants to be involved in research for altruistic purposes will dramatically decrease.

Regardless of how the use of genetic information obtained in a clinical context is ultimately regulated, genetic test result information that is returned to participants in a research context should be treated distinctly and should not be used by insurers. The UK's current moratorium has adopted this approach in respect of whole-genome sequencing carried out in major research projects.

Another area of concern is the rising trend of direct-to-consumer (DTC) genetic testing. This is available, often online, directly from a provider. There is variability in the industry as to whether a test is offered with a medical or scientific intermediary interpreting the results, or scrutinising their validity. The decreasing cost of DTC testing is leading to an increase in the number of consumers accessing testing for novelty or 'for interest' purposes, without an understanding of the potential insurance implications of that testing or the fact that this testing should be disclosed to insurers. Under current policy, there is no regulation regarding how these results might be used by life insurers. The working group submits that DTC testing results should not be used by insurance companies in making underwriting decisions without the oversight of an independent expert working group, and that until such a time as that group can be formed, DTC results should be excluded from consideration by life insurers.

Avenues for review of adverse underwriting decisions

This working group is concerned about the ability of applicants to access review or remedies in the case of an adverse determination by a life insurer. A particular case which exemplifies the difficulties with the current practice is that of James, as reported in a 2013 paper[3]. In summary, James was denied increased life insurance cover for cancer after having predictive testing for a gene known to be associated with increased risk of colorectal cancer, despite providing the insurer with evidence that with appropriate screening his cancer risk would be equal to population risk. The insurer ignored James' request for information regarding the basis of its decision, and only after he made an

---

[2] Personal communication, Associate Professor Anne Cust, University of Sydney
[3] L. A. Keogh and Otlowski (2013)

application to the Human Rights Commission did the insurer provide him with increased cover. James was well-educated and particularly tenacious – far from a typical consumer, for whom the avenues for challenging decisions by insurers are limited, not advertised and difficult to negotiate. Further, genetic information has the ability to affect other family members and a mechanism that requires public appeals processes that may inadvertently disclose genetic results to family members is inappropriate.

HGSA Position Statement

The Human Genetics Society of Australasia (HGSA) has published a position statement that specifically considers the use of genetic information by life insurance companies. The HGSA's position statement aligns with many of the comments, concerns and recommendations raised by the working group. Relevantly, the HGSA, inter alia:

- considers insurers should not require disclosure of genetic testing undertaken as part of a research project;
- considers insurers should implement a moratorium on the use of predictive genetic information pending improved actuarial estimates of the impact of that information on adverse selection;
- considers insurers should seek information from expert geneticists to assist with reviewing its actuarial modelling of the impact of predictable genetic disease; and
- supports implementation of legislation to protect against abuse of predictive genetic information.

Accordingly, the working group submits that the Commonwealth Government must reconsider the use of genetic information by the insurance industry and ensure that it is appropriately regulated.

REFERENCES

Cooper, D. N., Krawczak, M., Polychronakos, C., Tyler-Smith, C., & Kehrer-Sawatzki, H. (2013). Where genotype is not predictive of phenotype: towards an understanding of the molecular basis of reduced penetrance in human inherited disease. *Human Genetics, 132*(10), 1077-1130.

Joly, Y., Burton, H., Knoppers, B. M., Feze, I. N., Dent, T., Pashayan, N., . . . Van Hoyweghen, I. (2014). Life insurance: genomic stratification and risk classification. *European Journal of Human Genetics, 22*(5), 575-579. doi:10.1038/ejhg.2013.228

Keogh, L., van Vliet, C., Studdert, D., Maskiell, J., Macrae, F. A., St John, D. J., . . . Giles, G. G. (2009). Is uptake of genetic testing for colorectal cancer influenced by knowledge of insurance implications? *Medical Journal of Australia, 191*(5), 255.

Keogh, L. A., & Otlowski, M. F. A. (2013). Life insurance and genetic test results: a mutation carrier's fight to achieve full cover. *The Medical Journal Of Australia, 199*(5), 363-366. Retrieved from https://ezp.lib.unimelb.edu.au/login?url=https://search.ebscohost.com/login.aspx?direct=true&db=cmedm&AN=23992195&site=eds-live&scope=site

Minikel, E. V., Vallabh, S. M., Lek, M., Estrada, K., Samocha, K. E., Sathirapongsasuti, J. F., . . . Gambetti, P. (2016). Quantifying prion disease penetrance using large population control cohorts. *Science translational medicine, 8*(322), 322ra329-322ra329.

Natarajan, P., Gold, N. B., Bick, A. G., McLaughlin, H., Kraft, P., Rehm, H. L., . . . Seidman, J. G. (2016). Aggregate penetrance of genomic variants for actionable disorders in European and African Americans. *Science translational medicine, 8*(364), 364ra151-364ra151.

Otlowski, M., Taylor, S., Barlow-Stewart, K., Stranger, M., & Treloar, S. (2007). The use of legal remedies in Australia for pursuing allegations of genetic discrimination: findings of an empirical study. *International Journal of Discrimination and the Law, 9*(1), 3-35.

Otlowski, M., Taylor, S., & Bombard, Y. (2012). Genetic Discrimination: International Perspectives. *Annual Review of Genomics and Human Genetics, 13*(1), 433-454. doi:doi:10.1146/annurev-genom-090711-163800

Shah, N., Hou, Y.-C. C., Yu, H.-C., Sainger, R., Dec, E., Perkins, B., . . . Telenti, A. (2016). Identification of misclassified ClinVar variants using disease population prevalence. *bioRxiv*, 075416.

Song, W., Gardner, S. A., Hovhannisyan, H., Natalizio, A., Weymouth, K. S., Chen, W., . . . Willis, A. (2015). Exploring the landscape of pathogenic genetic variation in the ExAC population database: insights of relevance to variant classification. *Genetics in Medicine*.

Van Driest, S. L., Wells, Q. S., Stallings, S., Bush, W. S., Gordon, A., Nickerson, D. A., . . . Carrell, D. S. (2016). Association of arrhythmia-related genetic variants with phenotypes documented in electronic medical records. *JAMA, 315*(1), 47-57.

Walsh, R., Thomson, K., Ware, J. S., Funke, B. H., Woodley, J., McGuire, K. J., . . . Taylor, J. C. (2016). Reassessment of Mendelian gene pathogenicity using 7,855 cardiomyopathy cases and 60,706 reference samples. *bioRxiv*, 041111.

Appendix A – selected recommendations from ALRC Report 96 – "Essentially Yours: The Protection of Human Genetic Information in Australia"

- *Recommendation 27-1*: the Human Genetics Commission of Australia (HGCA) should establish procedures to assess and make recommendations on whether particular genetic tests should be used in underwriting mutually rated insurance, having regard to their scientific reliability, actuarial relevance and reasonableness;
- *Recommendation 27-2*: The insurance industry should develop mandatory policies for their members to ensure that, once the HGCA has made a recommendation in relation to the use of a particular genetic test in underwriting, that test is used only in conformity with the recommendation; and
- *Recommendation 27-3*: the insurance industry should require its members to state, on relevant insurance application forms, that not all genetic test results have to be disclosed and that applicants may obtain further information about this from the insurer.
- *Recommendation 27-5*: the Commonwealth should amend the Insurance Contracts Act 1984 (Cth) to clarify the nature of the obligation of an insurer to provide written reasons for an unfavourable underwriting decision upon the request of an applicant. Where such a decision is based on genetic information, including family medical history, the insurer should be required to give reasons that are clear and meaningful and that explain the actuarial, statistical or other basis for the decision.
- *Recommendation 27-6*: the insurance industry should require their members to inform applicants of their statutory entitlement to reasons for an adverse underwriting decision based on genetic information, including family medical history, and develop mandatory policies for their members about appropriate mechanisms for providing sensitive information to applicants in response to a request for reasons.
- *Recommendation 27-7*: the insurance industry should develop mandatory policies for their members regarding the provision of reasons by an insurer to an applicant following an unfavourable underwriting decision based on family medical history. These policies should ensure that the reasons given are clear and meaningful and that they explain the actuarial, statistical or other basis for the decision.
