# Supplementary material for: Should Australia Ban the Use of Genetic Test Results in Life Insurance?
Source: Front Public Health. 2017 Dec 13;5:330. doi: 10.3389/fpubh.2017.00330 (PMC5733354; doi:10.3389/fpubh.2017.00330)
Supplement: Supplementary file 2 [file Presentation_2.PDF]

Parliamentary Joint Committee Inquiry into the Life Insurance Industry **26/05/2017**

Australian Genetic Non-Discrimination Working Group Opening Submission

The use of genetic information by the Australian life insurance industry

Recommendations

1. **The Australian government should enact legislation to regulate the use of genetic information**
2. **Until this legislation is in place, the Australian government should enact a legislative ban or moratorium on the use of genetic data by life insurers, except for the use of negative test results to counter a family history.**

Summary of the issues

1. Our ability to use genetic information to improve healthcare is growing rapidly. However, it raises concerns around privacy and the potential for **genetic discrimination**.
2. The 2003 *Essentially Yours* Inquiry found that insurers' use of genetic information should be **carefully balanced by key consumer safeguards. However many of these have not been implemented or adhered to**. The Australian government has not revised regulation since 2003.
3. VIC, NSW , QLD and Commonweatlth governments have invested **$25M each** into the implementation of genomics in healthcare, **however the insurance implications have not been addressed**.
4. Internationally, **many countries have enacted moratoria or bans on the use of genetic information by insurance companies** due to the range of ethical, legal and social issues.
5. Currently in Australia, the life insurance industry largely **self-regulates the use of genetic information** and this is not in the public's best interest.
6. Insurance fears **deter the uptake of potentially life-saving genetic testing and participation in research** in Australia, at a time when maintaining public trust and developing industry is crucial.
7. The risk implications for many genetic changes are not supported by sufficiently robust data. **Much more research is required to understand genetics for prediction of future disease risk**.
8. **Little evidence has been provided to support claims of adverse selection** should the use of genetic information be prohibited in Australia.
9. The Group's initial submission made a number of recommendations. We've now arrived at the conclusion that **legislation and moratorium should be enacted**.
