# Supplementary material for: Should Australia Ban the Use of Genetic Test Results in Life Insurance?
Source: Front Public Health. 2017 Dec 13;5:330. doi: 10.3389/fpubh.2017.00330 (PMC5733354; doi:10.3389/fpubh.2017.00330)
Supplement: Supplementary file 3 [file Presentation_3.PDF]

COMMONWEALTH OF AUSTRALIA

Proof Committee Hansard

PARLIAMENTARY JOINT COMMITTEE ON CORPORATIONS AND FINANCIAL SERVICES

Life insurance industry

(Public)

FRIDAY, 26 MAY 2017

CANBERRA

CONDITIONS OF DISTRIBUTION
This is an uncorrected proof of evidence taken before the committee.
It is made available under the condition that it is recognised as such.

BY AUTHORITY OF THE SENATE

[PROOF COPY]

INTERNET

Hansard transcripts of public hearings are made available on the internet when authorised by the committee.

To search the parliamentary database, go to:

http://parlinfo.aph.gov.au

PARLIAMENTARY JOINT COMMITTEE ON CORPORATIONS AND FINANCIAL SERVICES

Friday, 26 May 2017

Members in attendance: Senators Ketter, O'Neill, Williams and Mr Irons, Mr Van Manen.

Terms of Reference for the Inquiry:

To inquire into and report on:

a. the need for further reform and improved oversight of the life insurance industry;

b. assessment of relative benefits and risks to consumers of the different elements of the life insurance market, being direct insurance, group insurance and retail advised insurance;

c. whether entities are engaging in unethical practices to avoid meeting claims;

d. the sales practices of life insurers and brokers, including the use of Approved Product Lists;

e. the effectiveness of internal dispute resolution in life insurance;

f. the roles of the Australian Securities and Investments Commission and the Australian Prudential Regulation Authority in reform and oversight of the industry; and

g. any related matters.

WITNESSES

ANDERSON, Mr Philip, Chief Operating Officer, Life and Investments, Zurich Financial Services Australia Ltd. 31

BAILEY, Mr Tim, Chief Executive Officer, Life and Investments, Zurich Financial Services Australia Ltd. 31

ENTHOVEN, Mr Richard, Executive Chairman, Greenstone Pty Ltd 18

GROBLER, Mr Brenard, Chief Operating Officer, Greenstone Pty Ltd 18

HAGGER, Mr Andrew, Chief Customer Officer, Consumer Banking and Wealth Management, National Australia Bank 1

LACAZE, Dr Paul, Head, Public Health Genomics, Department of Epidemiology and Preventative Medicine, School of Public Health and Preventative Medicine, Monash University; and Founding Member, Australian Genetic Non-Discrimination Working Group 59

LONGSTAFF, Dr Simon AO, Executive Director, The Ethics Centre 70

MILLER, Mr Greg, Executive General Manager, Wealth Advice, National Australia Bank 1

MORPHY, Mr Timothy, Chief Executive Officer and Director, MedHealth Pty Ltd 43

OTLOWSKI, Professor Margaret, Law Dean, University of Tasmania; and Chair, Australian Genetic Non-Discrimination Working Group 59

TILLER, Ms Jane, Legal and Social Adviser, Public Health Genomics, Department of Epidemiology and Preventative Medicine, School of Public Health and Preventative Medicine, Monash University; and Member, Australian Genetic Non-Discrimination Working Group 59

HAGGER, Mr Andrew, Chief Customer Officer, Consumer Banking and Wealth Management, National Australia Bank

MILLER, Mr Greg, Executive General Manager, Wealth Advice, National Australia Bank

Committee met at 08:52

CHAIR (Mr Irons): I declare open this hearing of the Parliamentary Joint Committee on Corporations and Financial Services. Today the committee is taking evidence as part of its inquiry into the life insurance industry. This is a public hearing and a *Hansard* transcript of the proceedings is being made. The hearing is also being broadcast via the Australian Parliament House website.

The committee generally prefers evidence to be given in public but, under the Senate's resolutions, witnesses have the right to request to be heard in private session. I remind all witnesses that, in giving evidence to the committee, they are protected by parliamentary privilege. It is unlawful for anyone to threaten or disadvantage a witness on account of evidence given to a committee and such action may be treated by the Senate as a contempt. It is also a contempt to give false or misleading evidence to the committee.

If a witness objects to answering a question, the witness should state the grounds of the objection and the committee will determine whether it will insist on an answer, having regard to the ground which is claimed. In addition, if the committee has reason to believe that evidence may reflect badly on a person, the committee may direct the evidence to be heard in private. Witnesses should be aware that if, in giving their evidence, they make adverse comments about another individual or organisation, that individual or organisation will be made aware of that comment and given a reasonable opportunity to respond to the committee.

On behalf of the committee, I would like to thank witnesses here today for their time and cooperation and also thank my fellow committee members, the secretariat and Broadcasting. The committee welcomes the National Australia Bank. I remind committee members and witnesses that, while this is a public hearing, care should be taken to protect the privacy of individuals and that arguments should be made without naming individuals. I now invite you to make a short opening statement and, at the conclusion of your remarks, I will invite members of the committee to put questions to you.

Mr Hagger: I would add that I am also here as a director of MLC Limited. We pride ourselves in serving the life-long financial needs of generations of Australian families and offering certainty when they need it most. This parliamentary joint committee is inquiring, particularly, into life insurance. I am proud of what I and NAB have done in providing hundreds of thousands of customers with not only the peace of mind that comes with insurance but also the actual outcomes. In 2016 alone over 8,000 customers and their families were paid over $900 million, with fewer than one per cent of denied claims disputed externally.

In October last year NAB sold 80 per cent of our life insurance business to Nippon Life, one of the world's largest specialists in life insurance. I was the executive responsible for recommending to the NAB board Nippon Life as the best future owner of that business I could find globally. This was after a period of due diligence that I conducted with my team. The MLC Life business is in extraordinarily good hands. Nippon Life will bring innovation, investment, patience and focus to the development of MLC Life.

NAB retains 20 per cent ownership of the new life insurance business and we have entered a long-term partnership to provide life insurance products through NAB's own and aligned distribution networks. Members of the committee, I know you have followed the progress of NAB's Wealth Management division and my responsibility as the head of that division from April 2013. As I have stated from the beginning, we are proud, not perfect. And we are strengthening things, fixing things. Our record remains amongst the very strongest, in this regard, in the industry.

When I came into the role of group executive of NAB Wealth, in 2013, I made it my mission to lift both the culture and performance of NAB's wealth business, to drive change and to find things that were wrong and needed to be fixed. This included fixing existing issues, looking for new issues and making things right for customers and, then, improving systems and controls to prevent those issues from reoccurring. And this is what I have done. I have led the implementation of our five customer-response initiatives, which are market leading, and have overseen the mediation of various fees and advice for customers.

Our focus on improving standards and performance is ongoing, and with our focus on our customers we may continue to find issues. This is what happens when you constantly look to raise the bar for customers. It is my job to lead that process of getting it right and, through doing that, we will continue to build trust and confidence in NAB. At NAB we believe in life insurance and we believe it is in our customers' interests that they consider it. Greg Miller and I look forward to your questions this morning.

Senator WILLIAMS: My first question is about MLC. Are you involved in group insurance?

Mr Hagger: MLC insurance is involved in group insurance.

Senator WILLIAMS: And you do direct insurance, where customers come straight to MLC.

Mr Hagger: Yes.

Senator WILLIAMS: And, of course, you do personal insurance, where a financial planner gives specific advice to a customer and what sort of insurance they need.

Mr Hagger: That is right, yes. In fact, that is the bulk of it. In retail advice, insurance is 65 per cent of the MLC Life business. Group insurance is about 30 per cent. The other five per cent is direct.

Senator WILLIAMS: With your direct insurance, you just advertise on TV, do you? Do you have brokers, different companies, and you underwrite through MLC when they advertise all sorts of insurance, life insurance?

Mr Hagger: There is some insurance advertising on television, sometimes, yes.

Senator WILLIAMS: Can you give me an example?

Mr Hagger: There is an ad campaign going on, at the moment, during breaks of *MasterChef* or whatever the case may be. In that ad we show the picture of a woman who has had a dinner party with her family and friends around her. She says, 'Really, all I want in life is exactly what I've got right now.' The point being made in the ad is that insurance provides protection for people because unexpected events might occur. For all that families have to deal with at those kinds of moments, insurance can play a very important role in easing financial burdens.

Senator WILLIAMS: Let us say I am watching TV and see your advert. I think, 'Yeah, I'll ring up and I'll take out a death policy for $500,000.' I give my credit card and say, 'Take the payments out each month.' I am not actually underwritten, am I?

Mr Hagger: With the digital products for what you are describing, which is life insurance for lump sum term life, no, you are not underwritten at the time of take-up.

Senator WILLIAMS: Is it the same in group insurance if I had it with my superannuation—I have not; I cancelled it because it was such a terrible policy: $36 a month for $23,000, so I canned that. Group insurance is not underwritten either. Is that correct?

Mr Hagger: That is largely correct. There are questions that are asked at the time of taking out a policy, but the concept of group insurance is that you have got a very big group of people—often 1,000 or 2,000 people. What happens under group insurance is that you are addressing a bigger volume of the population and you know that there will be incidents within that population—for example, death, as you mentioned. So it is quite a different experience to what happens through the retail advice area, where there will be a more specific, tailored approach to your insurance needs.

Senator WILLIAMS: If I took out direct insurance for, say, life insurance and all of a sudden had a heart attack and dropped dead and then the insurance company went back through my history and saw that at two years old I had a valve replaced in my heart, I was born with a hole in my heart and I had a history of heart disease, that could give the company reason not to pay the claim. Is that correct?

Mr Hagger: It depends how you answered the questions at the beginning when you took out the policy.

Senator WILLIAMS: But with direct insurance you answer very few questions. This is the point I am making. You do not answer many questions with direct insurance, do you?

Mr Hagger: No, you do not.

Senator WILLIAMS: What I am saying is that, if I died of a heart attack and I had a history of heart disease, heart problems, heart surgery et cetera, when the insurance company finds out about my history, that can give them reason not to pay the claim. Is that correct?

Mr Hagger: It depends on how you have answered the questions. If you have answered the questions truthfully, the cover has been taken out and you are not excluded then the claim would be paid. In fact, we pay claims like that.

Senator WILLIAMS: With direct insurance, do you get that many questions? We know that with personal retail insurance the financial planner will take you through everything and explain everything and you will tick the boxes. When you take the policy out, you are underwritten. With direct insurance am I right when I say you do not answer many questions?

Mr Hagger: That is right. You do not answer as many questions.

Senator WILLIAMS: With direct insurance would I get a question about whether I had any history of heart problems?

Mr Hagger: That would be a typical question that would be there. Maybe it is best if I give you an example, because I think what you are testing is whether claims approaches in direct insurance pass the pub test. I think that is at the heart of this.

Senator WILLIAMS: Yes. Here is the problem I have got. People take out direct insurance and then, when the insurers find out the history and details of that person for a claim, they are rejected because of some past history that was not disclosed. But they were never asked about it. If a young bloke gets married, has a couple of kids and a mortgage on a house and takes some insurance out direct but all of a sudden gets run over by a bus, there is no payment, because of his history, yet he was sleeping peacefully at night, thinking, 'If something does happen, my wife and my children are covered and can stay in the house' et cetera. This is the problem I have.

Mr Hagger: I understand the problem. The way we look at it—and I am speaking here on behalf of MLC life insurance, where we are a shareholder in the business and do not run it but did used to run it. We used to run it—and on my watch—so I want to give you an example. There was a customer who took out some direct insurance with us who was suffering from depression. There was a direct question on depression, and that customer did not answer with the details about that.

Senator WILLIAMS: So the question was, 'Have you ever suffered depression, mental illness or whatever?'

Mr Hagger: That is right. The customer said 'no' but they had and they had been treated for it et cetera. The depression got worse after the time they had taken out the claim. Their employer let them go and they could not find employment. So in that situation we are faced with a dilemma as an insurer.

Senator WILLIAMS: Was this income replacement insurance?

Mr Hagger: Yes it was. In this case, whilst in the legal sense the customer had not disclosed everything they should and, really, in a legal sense, therefore there was no claim to be paid, we decided to pay an amount to that customer to help them with involuntary unemployment.

One of the things that we feel strongly about is the difference between the t's and c's of the terms and conditions, and trust and confidence. There are times that MLC Life has paid claims when the terms and conditions say something different. We want to pass the pub test. People should feel when they take out an insurance policy that, if things go against them, that the insurer is there for them. So we want to see those claims paid. That is how you get to have a long-term business.

There are features of the direct insurance business—and I know this committee is very good at looking very carefully at that—which are different in characteristics to group insurance and to retail advice. Actually, the kind of insurance we like the most is retail advice insurance. You can see that by the shape of our book. We would love to see all Australians get quality financial advice and get quality insurance outcomes but we know that many customers in this modern world want to reach out for insurance online—they want to do things directly—and also, for group insurance, it does handle a large volume of people. So in those cases, MLC Life offers those types of insurance.

On the NAB, Greg Miller leads our advice practice. Our advisers want more business. We want to grow. We want to grow more retail insurance in addition to group insurance and direct insurance.

Mr VAN MANEN: I want to follow on from where Senator Williams was going with this. I would be interested if you could provide for the committee an application for direct insurance and a copy of an application for retail life insurance so it gives us some more detailed information between the two.

Mr Hagger: We would be very happy to do that.

Mr VAN MANEN: I do not know if you can do that for a group life policy as well but if you have got the capacity to do that, it would be appreciated. Then we can see more plainly in front of us what the difference is in the detail of what you are asking. In the direct insurance, I have a concern. I would be interested in what training you do with your people that are taking the phone calls or processing these applications for direct policies as to what questions or what discussion is being had with potential customers through the direct channel about replacement or other activity on their existing insurance policy. So are there questions being asked of those people as to whether they have existing policies? Are there questions being asked of those people as to whether this new policy is replacing an existing policy? I have questions in that area because I have a concern that if there are questions or discussions being had in that space, that is beginning to border on advice. As a consequence, if that is starting to border on advice then what work is being done to ensure that that discussion is being properly documented and those potential customers whose lives are insured are being properly protected?

Mr Miller: We agree with you. If somebody starts to talk about the placement of policy and other matters, that is absolutely delving into personal advice. We are quite considered and regarding the different ways you might come into our organisation—whether it be digitally, phone or talking to an adviser—you are actually looked after in the right way and we follow the right legal approach as per Corporations Law. We are very strict there. In some of our cases, you can only apply for our insurance online, for example. So it is straight through with the customer completing the information.

Mr VAN MANEN: In that process, is there any human interaction or is it purely an online application?

Mr Miller: Some of it is purely online. For some, where there is a human interaction, we are very particular about what is said to the customer; that it is general advice or information only.

Mr VAN MANEN: Are those discussions recorded?

Mr Miller: We record those conversations. We then listen to those for quality and compliance to make sure that people are following it in the right way. It is also used if a customer at any stage says, 'I was given that advice or told this information.' We can get that recording and listen to those recordings. So it is very important that each person when they are dealing with a customer knows what their boundaries are under licence. You cannot give personal advice if you are not a licensed adviser and an AFSL holder. We are very clear about those components. We look at that across all of our chain across our group to make sure that it is properly looked after. Then send off hand-offs. If somebody says, 'Gee, I've got another insurance policy; maybe I should think about whether this is the right one for me,' that is an advice discussion and we would hand that on to an adviser to have the proper and fit discussion.

Mr VAN MANEN: I would like to move to remuneration. I know Nippon Life now owns 80 per cent of MLC. However, are there any differential remuneration arrangements for your advisers internally for business written to MLC as opposed to other products?

Mr Miller: No, there are not.

Mr VAN MANEN: What is the breadth of your approved product list?

Mr Miller: Across our broader approved product list, we have nine companies on our approved list. That is the total list and then depending on licensees, it is a subset of that. Our salary channel currently has two and is about to go to a third. Our MLC advice franchise is about to go to its third. Our other self-employed licensees have somewhere between four and eight, depending upon the business that they are in. That is because in the self-employed world an adviser might choose a certain niche demographic or occupation; therefore they need to think about what ones they need. So we do it slightly differently. We have nine across our broader approved list.

Mr VAN MANEN: At what level are those approved product list created? Are they assessed at a Nippon Life board level or are those created for each individual licensee at a licensee board level?

Mr Miller: Nippon Life have nothing to do with our approved lists—that is really important. We set that at the advice licensee level. We have an internal research team, 360, that do the research. They adjunct to that research from the likes of Lonsec, Morning Star, Dexar, IRESS researcher to look at that. They then make their decision on what they think is most appropriate for our various licensees in putting together. It then goes through an investment and product committee. That investment and product committee is chaired by a non-executive director and there are two non-executive directors on that committee as well as some management. It is goes through that process before it is put on our approved list. Nippon Life or MLC Life have nothing to do with that process.

Senator WILLIAMS: I bet you when you play football you hog the ball!

Mr VAN MANEN: I am a goalkeeper.

CHAIR: Only when he can get it off you.

Senator WILLIAMS: Mr Miller, you are in charge of the financial advice in NAB. Have you got your house completely in order now given the Graeme Cowper episodes et cetera?

Mr Miller: Andrew summed it up at the start by saying we are proud but not perfect. We have been working diligently over the last two years to make sure we continue to improve what we do. That is not to say that some adviser will not do something that we do not like in the future. But we are doing everything possible to make sure we improve, and we have been improving our monitoring, supervision and compliance over the last two or three years. We have implemented a new process for compliance—

Senator WILLIAMS: Yes, we put it under the spotlight; didn't we?

Mr Hagger: You did, yes. We have done a range of things to improve that, and that is important to us. We will continue to improve our monitoring and supervision and those things we are putting in place—

Senator WILLIAMS: It is important to the people—especially the people—

Mr Miller: It is.

Senator WILLIAMS: when you have roads and people doing the wrong thing. The reason we are having this inquiry was the CommInsure *Four Corners* program. Their medical criteria for claims was outdated—for example, troponin levels for heart attacks, et cetera. James Kessel from Wee Waa featured on the program. Does MLC have those medical criteria as well?

Mr Hagger: MLC has updated heart definitions. That is one of the first things we looked at after we had seen the program. The other thing we did at that time was—

Senator WILLIAMS: After you had seen the program?

Mr Hagger: What I am saying is, when the program came and shone a light on CommInsure—

Senator WILLIAMS: It did.

Mr Hagger: and I cannot comment on CommInsure; I do not know their situation—but the first thing we did was to announce a claims assurance review to make sure that our definitions et cetera were where they needed to be, and our heart definition was where it needed to be at that time. We have continued on with our claims assurance review since then under the chairmanship of Malcolm Arnold, who is our ex-FOS. He is the independent chair of that claims assurance review. And on an ongoing basis—and I think the Life Insurance Code of Practice will help with this—there will be an updating of definitions. I think updating of definitions will be a feature of the industry for a long time to come—

Senator WILLIAMS: I hope so, yes.

Mr Hagger: because there are medical advancements.

Senator WILLIAMS: Do you use IMEs?

Senator O'NEILL: Can I just ask a clarifying question?

Senator WILLIAMS: Certainly, Senator O'Neill.

Senator O'NEILL: What is your current definition of troponin, and is it the same as other players in the industry?

Mr Hagger: Yes, it is the same as those that have updated their definitions.

Senator O'NEILL: What is it?

Mr Hagger: I would like to take on notice the specifics of that.

Senator O'NEILL: Thank you, Mr Hagger. Do you know if that is consistent across the entire sector?

Mr Hagger: I do not know about other people's policies. The only ones that we know about are those that are on our product list.

Senator O'NEILL: This is about the definitions—and I know that Senator Williams is right onto this as well—this timing of changing of definitions: the differences between the insurers is a concern to ordinary purchasers of the product. They think a flood is a flood, a heart attack is a heart attack. They expect there to be a commonality of definitions. Is that the case currently?

Mr Hagger: I think what they expect is to pass, what I think we all call, the pub test.

Senator O'NEILL: Yes, but is it the same across the sector currently, or is it varied?

Mr Hagger: I do not know everybody's product features. People are updating their products all the time. What we look at—

Senator O'NEILL: That is right, Mr Hagger. The point is: if you do not know, how does an ordinary Australian know? Then you spoke in the second part of your answer about the capacity of the industry itself to come up with definitions. I would be very interested if there is a proposal for standardising definitions across the industry so that there is less confusion for an ordinary purchaser of the product.

Mr Hagger: I think the life insurance code, which has been entered into, talks about the updating of definitions. But, yes, a flood is a flood and, yes, a heart attack is a heart attack, and I began this by saying—

Senator WILLIAMS: It used to be like that.

Mr Hagger: I would like to give you an example of that. In the case of the floods in Queensland of 2010-11. Around that time NAB did not manufacture general insurance, but we have a partnership with a general insurance

manufacturer, and many people in Queensland who went through the awful things that happened with the flood then looked at their insurance policy, and it did not cover for flood. We decided, at NAB, that it was the right thing to do to pay out to those people, and we did that. We have a very proud record in this area, because our focus is on trust and confidence beyond terms and conditions. Terms and conditions are important. The insurance industry is very much built on actuarial input and really the rules that go back to maritime insurance of the 1600s—

Senator O'NEILL: I will come back with some more questions on that. I am aware of taking away from Senator Williams. But there is no standard definition across the sector at this point in time? The industry has not imposed that on you, have they?

Mr Hagger: No, the industry has not done that. And I am not sure that would be the best approach. If we see the need to update a definition—or MLC Life does; I need to be careful because they manage the business—why should they wait for the rest of the industry to update the definition? The life insurance code, as I recall it—and I am happy, if I am wrong, to correct the record—says that definitions will be updated periodically. But there is nothing to stop an insurer jumping ahead of that, and nor should there be, in my opinion.

Senator O'NEILL: It depends on whether the change is in favour of the customer or in favour of the insurer.

Mr Hagger: I am happy to come back to you on that.

Senator O'NEILL: Thanks, Senator Williams.

Senator WILLIAMS: I will ask the question: why does it take a TV program to clean up the industry? People should have been aware that those troponin measurements have been outdated for years. Yet James Kessel has a heart attack, he is in the emergency ward of the Guyra hospital, his heart stops beating, he tells me he heard the nurse say, 'I think we've lost him,' and then they said he did not have a heart attack. Give me a break. I want to go to IMEs, independent medical examiners. Does MLC use them?

Mr Hagger: I believe so, yes.

Senator WILLIAMS: How are they remunerated?

Mr Hagger: That I do not know. I would have to take that on notice and ask MLC Life to answer that.

Senator WILLIAMS: We might be able to find out later on from some of the other insurance companies. I want to know how these IMEs are remunerated. We will have MedHealth in front of us later on—Mr Tim Morphy, CEO of MedHealth—and we can ask him as well. I want to know how they collect their money. Do they get a share of the doctor's fee when they employ the doctor? Does the insurance company pay them, et cetera? We can come to that afterwards. I will hand on to others. We have plenty of time. I will come back with more questions, thanks.

Mr Hagger: Chair, may I just make an update. I have just looked at my notes. I have here that the code of practice will include standard definitions for cancer, heart attack and stroke resulting in specified permanent impairment. So there are some things in the code. There are lots of definitions in the insurance industry for a lot of potential medical errors. I know mental health is a particular area of interest to the committee, which I am happy to speak to. MLC Life has already stated that we will pass back the new definitions for all policies written in the two years preceding the introduction of standardised medical definitions. In these key areas, the code, which comes in in July 2017—in just a few weeks time—will incorporate those aspects, and I think that will be positive for the industry.

CHAIR: On that, I did ask the FSC, when they were at a hearing, how close the industry was to being ready to jump on 1 July to that code. They could not actually advise me how prepared they were. They said they had been having plenty of training. How close are you to switching?

Mr Hagger: I believe MLC Life is ready for the introduction of the code in July 2017. Looking through it, it was pleasing to me that most—if not nearly all of what is in the code—are already practices that MLC Life follows.

Senator O'NEILL: I will go to mental health immediately. I have lots of questions for you. What barriers and policy exclusions are used to prevent or deny claims generally, and then in particular with regard to mental health?

Mr Hagger: Firstly I will answer in relation to mental health. We have no blanket exclusions across the MLC Life portfolio. I believe there is one direct product which has an exclusion for schizophrenia, in which case there are other parts of insurance offered.

Senator O'NEILL: What is that one direct policy?

Mr Hagger: Let me go to my notes. I want to answer correctly on that.

Mr Miller: In our Essential Life cover, the underlying question is whether or not the customer has been diagnosed with schizophrenia. The customer who answers yes is offered accidental death only. It is made clear to the customer, therefore, what they are covered for in terms of that.

Senator O'NEILL: So they are offered accidental death only. Would being refused insurance be something that they would have to then declare at other points of inquiry with other companies?

Mr Miller: If they were going through an underwriting process, for example, they would talk about that, but then they would have the opportunity to also submit their medical records and go through that and have an assessment. Obviously, one of the advantages of underwritten insurance is being able to put all the information on the table to an insurer.

Senator O'NEILL: Or they could go through a direct process, as Senator Williams was discussing earlier, and answer questions online or over the telephone and not disclose that.

Mr Miller: That is correct, they could do, and if they did not disclose—

Senator O'NEILL: Then what would happen to their claim?

Mr Miller: That would depend on which company they went to and how they saw that.

Senator O'NEILL: So some companies will cover for schizophrenia, but you have one product that does not.

Mr Miller: We have one product that does not, but, on the question of other companies, I cannot answer that.

Senator O'NEILL: If somebody was refused at another insurance company and they went to MLC through to you, would they be asked if they had been denied insurance elsewhere?

Mr Miller: They could be, depending on which contract they come through.

Senator O'NEILL: So there is a chance that somebody might have every door shut to them in seeking insurance once they have been refused by one?

Mr Miller: I cannot comment on that because I cannot comment about what all insurers would do in relation to that.

Senator O'NEILL: Would you be able to take that on notice to find out a bit more about what your policies are around refusal of insurance for people who have had a refusal somewhere else. This is one of the issues that has been raised with us.

Mr Hagger: We will take that on notice.

Senator O'NEILL: Is schizophrenia the only diagnosed mental illness that you have an exclusion around?

Mr Hagger: I can take that on notice, but I believe so. We, meaning MLC Life, pay $50 million per annum in mental health claims, and we would expect that to rise. The emergence of mental health is something that the industry is challenged by and adapting to. Also, for us, one of the key areas that might be helpful to this committee is that we believe there would be virtue if we could make targeted payments for medical and rehab in areas of mental health for psychological support. At the moment, we are unable to do that. To give you an example, I think mental health makes up nine per cent of the incidents of the book—

Senator O'NEILL: Of the MLC Life book?

Mr Hagger: of the MLC Life book—but is something like 25 per cent of the ongoing claims portfolio. That means that these claims run for quite a period of time, and sometimes those claims run for some period of time because the insured is not able to access the psychological support that they need.

Senator O'NEILL: We have had this problem explained to us on a number of occasions now, so we are aware that the industry is seeking that capacity to make a change.

Mr Hagger: Yes, so we would like to add our voice to that, please.

Senator O'NEILL: What is the practice of life insurers in regard to obtaining information from a claimant's treating doctor?

Mr Hagger: What I know about that is that the insurance code is fairly specific about what will be required as of July 2017, and I believe those practices are the practices that we follow today. In the code, it talks about aspects relating to when the insured goes to see an expert. I believe you asked questions of the MLC Life team when they appeared, so I think I would direct you to those comments and to the code.

Senator O'NEILL: Going to some of the evidence that we got from MLC, it sounds like you have read their evidence and are aware of the disclosure of the ticking of a box that would give MLC access to the entire medical health records of anybody who signs up for insurance with them. Are you aware of that?

Mr Hagger: Obviously the evidence is the evidence; if I direct you to it and that is what they have said, then that is what they are doing. I do not know, but what I recall of the evidence is that Mr Hackett and Mrs Eckersall discussed how the approach of MLC insurance is very targeted and very limited in relation to how it operates in areas of accessing of records and—

Senator O'NEILL: Yes, that is correct. That was the qualifying thing that they gave at the end. But is it still the policy of MLC life insurance—in which you still have a 20 per cent stake—to allow people to unwittingly tick a box that gives their entire medical health records over to the insurer?

Mr Hagger: I will take that question on notice.

Senator O'NEILL: I am a little surprised by that. Not to be too—

Mr Hagger: We do not manage the business. Mr Hackett and Mrs Eckersall have answered your questions on that. They are closer to it than I am. I am aware of the code and I am aware of their evidence. I think you are asking me to go beyond that. I am happy to take that on notice. If we can go beyond that, then we will.

Senator O'NEILL: I take you to your opening comments where you talked about a cultural lift, existing systems, new systems and improving standards and performance.

Mr Hagger: Yes.

Senator O'NEILL: One of the things that is really important for people's health and wellbeing, particularly mental health, is the safety of their disclosure of their illness to their doctor. In their interactions with an insurance company, I think most Australians would be absolutely outranged to think that they have inadvertently ticked a box that gives their insurer access to files that they probably have not even seen themselves and which are about the intimate nature of their own body and their interaction with the health system in this country. I have had conversations with members of the Royal Australian College of GPs and I have seen statements from the AMA that indicate they are extremely concerned about this as a practice and that, in fact, it goes against the oaths that doctors make about the sanctity of the private relationship between doctors and their patients.

Mr Hagger: I am very happy to take your question on notice. There was the time prior to us selling the business to Nippon Life Insurance. If I think back, one of the things I used to do was look through our complaints process. From time to time, I would hold discussions with the AMA and other various other bodies. This is not something that came out as a key issue for us, and it was probably because of the care taken in the use of information in the application of it and the approach that the company took. Nippon Life are now the owners of the business. They will take this to a whole new level. The industry itself is clearly improving—

Senator O'NEILL: What you mean by that? A more invasive level? A whole new level? What are you talking about there, Mr Hagger?

Mr Hagger: Nippon Life is a fantastic life insurance company. It is one of the top global insurers. It operates in a number of countries, it has a very strong customer-first methodology, and it knows that it has bought a business which is in a very dynamic industry. As changes are occurring in mental health, in technology and in other areas, Nippon Life is very well equipped to respond to those challenges. One of the reasons why we sold the business to them rather than to somebody else is that we have every confidence that they will strengthen the business and improve it in a way that means it has a very customer-first methodology. They are here for the long term. You do not build long-term businesses if you are outraging customers. The whole approach is—

Senator O'NEILL: Is that why you sold your share in it—to change the nature of your relationship with your customers?

Mr Hagger: No. In fact, we kept 20 per cent. I am the chief customer officer of NAB. The reason why we sold it, which we were very clear about in the announcements, was that insurance requires a very large capital base. Insurance is a very capital-intensive product and—

Senator O'NEILL: Can I go back to my surprise? It has been put on the record before this committee that MLC is currently undertaking a practice where a tick of a box gives the insurance company, which you are intimately connected with, access to Australians' entire medical records in a way that is generally very unclear and unknown in the community, and you did not immediately act to change that. It seems to me that that is an indication of a cultural practice that is a very significant breach of trust.

Mr Hagger: I am very happy to take your question on notice. Perhaps, once you see the answers to the question on notice, we can have another discussion.

Senator O'NEILL: I will point out to you that MLC, in their response to us, did indicate that it was very likely that those seeking insurance would be unaware that that was what they were doing when they ticked that box, and I think that is where the breach of trust really comes in. Also, could I counsel you to undertake, seeing as

you are a significant player in this space, conversations with the relevant medical bodies—the RACGP, the AMA and others—to negotiate a much more ethically sound way of ensuring that you get the information you need, as an insurer that is your right and business, while the protection of the privacy of ordinary Australians is maintained. I think that is a very significant—

Mr Hagger: I appreciate your comments and, in taking the question on notice, I will also look further into it, of course. We will give you the response so that we can have another discussion. Should that lead to further discussions with the AMA and changes to practices, then, through the management and the governance processes, we will certainly explore that and consider that seriously.

Mr VAN MANEN: I want to follow up on an earlier query around medical definition updates. I have discussed the issue of legacy products with members of NAB previously. Given now the sale of the business to Nippon Life, what is the process of managing the legacy product caseload? MLC, as a long-standing business, would have some very old products—

Mr Hagger: Yes, we do.

Mr VAN MANEN: as I know some of the other insurance companies do. To take that a little bit further, you mentioned in your testimony earlier that you are going through a process of updating some of you medical definitions back two years for the standard definitions that will be included in the life insurance code of practice. But what are you doing with other legacy products that obviously are a lot longer tailed than two years in terms of dealing with some of the legacy definitional issues, medical definitional issues, that Senator Williams raised earlier?

Mr Hagger: Mr Van Manen, you make a very good point. We do have legacy products—or MLC Life does have legacy products. When we sold the business, we sold the legacy products with that. I think the APRA commissioner, Mr Summerhayes, has spoken on the record about legacy products just in the last day or two; he was asking for further investment in the systems that sit behind them. As a company, MLC Life is investing hundreds of millions of dollars in systems.

In relation to definitions, this is probably a very important issue for the committee to consider because we have quite a rigid structure in insurance legislation around 'guaranteed renewable'—that is the phrase. What can happen over time is that definitions can change. When these legacy products occurred, mental health, for example, was not something that was on anyone's radar screen. An adviser will sit with a client and work through what does that all mean for them.

In terms of the insurance company, one of the ideas that MLC Life would add to others about this inquiry is to look at the rigidity of the guaranteed renewable approach, because I think that needs to become more adaptive. Life insurance is a very adaptive, dynamic industry, and yet the legacy products are caught up in what provides a lot of difficulty in terms of the rigidity of that regime. That is something we would suggest that the committee looks at. I do not think it is great to have an industry that has a rigid structure when it is actually dynamic in its movements. All of the areas you are rightly looking to on consumer protection, they are dynamic areas. They require adaptive approaches. If you look at general insurance and some of the other insurance industries, they have got much more adaptive processes, and I think that is helping those industries get stronger, whereas that is not the case in life insurance. I think it is worthwhile for the committee to take a close look at that.

Mr VAN MANEN: The important thing from my perspective is: how do we reduce the cost of insurance to consumers and therefore make it more attractive? Obviously, there is a significant interest to government for people to be adequately insured because, if they are not, government ends up wearing some of the costs through our welfare system. What would be the value to the life insurance industry of a more flexible arrangement and to borrow something from the IR space—something similar to a better-off overall test? You go to APRA and you seek an application to say, 'Well, we want to move these clients into this particular product. They will be better off overall even though some of the definitions may change.' What would be the impact on the industry of doing that; and what is the overall benefit to consumers in terms of product definitions but also cost savings to their insurance policies?

Mr Hagger: I think you are onto a really key point. There will be benefits for consumers. What will happen, if there is more adaptiveness in the regime, is that there will be more trading up out of legacy products just as you mentioned in other areas in the investments field. As that happens, it means that customers will come onto modern products with modern definitions, and there is a whole flow to that. I think that will be very healthy for the industry. It will also help in relation to systems. I think we were quoted—we, meaning MLC Life—in the paper as having some microfiche records on a legacy product, which is true for record keeping our purposes. I

think it would be helpful for the industry to upgrade these products, but we need the regime and the assistance of the regulator to help that to happen.

CHAIR: Mr Hagger, just for the record for the committee: I just looked up MLC, and it came up with MLC Limited retail banking company. It has got you stated as the CEO of MLC Limited—is that correct?

Mr Hagger: I think that would be a problem with Google, Chair. If I can just explain: MLC Limited is a 130-year-old company.

CHAIR: So 1886.

Mr Hagger: Yes. It was started by James Garvan, because he wanted every Australian to have life insurance. Over the years, MLC also became an investments company, so MLC Private Equity is famous for bringing Facebook and other things to Australian customers. At MLC Limited, say, two years ago, we were operating the investment side, the advice side and the life insurance side under the MLC name. Then we had to unscramble MLC Limited in order to sell the life insurance company, and so we went through a process of successive fund transfers, which had legacy product aspects to it. We emerged out of that with MLC Limited, the life insurance company, being sold to Nippon Life with other aspects of MLC on the investments and advice side remaining with NAB.

MLC Superannuation Fund is Australia's largest retail super fund, and that is a NAB-run organisation. MLC Advice, Mr Miller oversees. I apologise for the confusion, and this morning I am trying very hard to give you answers as an MLC Life board director the best way I can. If there is anything I am saying which is at odds with management in any way, please take their comments on the record, and I will correct mine, because they are closer to this business than I am. But I am on the board. I want to help the committee, and MLC—

CHAIR: I just wanted to establish—

Mr Miller: And we run the brand across a couple of entities, so there is the MLC Superannuation Fund that is part of the NAB world—we use the brand there—as well as the MLC brand used in life insurance, which is 80 per cent owned by Nippon.

CHAIR: Just to go back to a couple of questions by Senator Williams in relation to online—I guess you would call it low doc—insurance. Do you think customers should be made aware that they are not actually underwritten at the time they start paying their premiums?

Mr Hagger: Firstly, there is a whole group of customers that want to deal through that channel. They are simple products. If they were underwritten the premiums would be much higher. So it is getting that—

CHAIR: That is not what I am asking you. Do you think they should be told that they are not underwritten?

Mr Hagger: I think their exclusions are very clear to customers through the direct channel. When they purchase the product—through the product disclosure statements and through the questions they are asked—I think it is made clear to them. Occasionally, as mentioned earlier, a claim will come, and then we will assess: is that fair in the circumstance? As I mentioned, the foremost tenet here is that we want to build trust and confidence in the business.

CHAIR: What weight does a report from an independent medical examiner—employed or under contract to the insurer—have in comparison to a report from a claimant's treating doctor? Is there a weighting to the report? You can take it on notice if you like.

Mr Hagger: I do not think there is a strict formula, but typically it is a claimant's treating specialist that we think is best positioned to provide insights into the claimant's experience, their current condition, their symptoms and their likely prognosis. There will occasionally be differences between medical professionals, and we see that. I mentioned at the beginning, in my opening statement, that we have around one per cent of disputed claims taken externally. It is in those environments that you often see differences in medical experts. Sometimes we need some independent checks, whether that is because the medical science is still growing in that area or because we think the doctor, for whatever reason, was not as experienced in that area as they could be, or whatever. But the primary specialist is the claimant's treating specialist.

CHAIR: After 96 years MLC moved under the banner of Lendlease—Lendlease acquired it—and then, in 2000, NAB acquired it. Now you have moved 80 per cent of it off to Nippon Life. We have heard from quite experienced salespeople who work for MLC or other mutual benefit companies about how the attitudes of mutual life companies were different to the current status of assessments; the mutuals were there for the benefit of the policyholders, not for the benefit of the shareholders. But it is interesting that you have now moved into another arena where Nippon Life is a mutual company.

Mr Hagger: Yes, it is mutual.

CHAIR: So we have a Japanese company—a mutual company—that owns 80 per cent of a company in Australia but none of the policyholders in Australia will get the mutual benefits that the Japanese Nippon Life policyholders will get. Do you see it as a strange area where maybe the attitude of insurance in Australia has gone from being for the benefit of the policyholders to purely a shareholder-benefit situation? Would it not have been easier to move back to a mutual so the Australian policyholders could get benefits?

Mr Hagger: In a moment I will ask Mr Miller to comment because he has been with MLC for quite a period of time. But first, I think Mr Hackett made some comments in his testimony that, as far as Nippon Life is concerned, the Australian policyholders are family to them. In my opening statement I pointed to areas in which I believe Nippon Life is very well situated to take forward the MLC life business. These are features that come with the Japanese life insurance approach, which is about patience, focus, innovation, specialism and customer-first methodology. Over the time from 2000 to 2015, when we announce the sale to Nippon Life, I believe that NAB was a good steward of that business. I believe that Nippon Life will be a great steward of the business going forward.

CHAIR: I am not asking what the NAB's strategy is, but I guess NAB did not see it as a core part of their business?

Mr Hagger: It came down to the issue of capital. I think you are seeing, across the life insurance industry at the moment, that international players are coming into the market. When you look at the amount of capital required—for NAB shareholders, this was a very capital intensive business. From a regulatory perspective, to be a modern-day bank we need to be unquestionably strong. Even as we speak, APRA is working through its requirements for unquestionably strong capital within banks. In a world where the NAB wants to be strong on capital, we have to use that capital wisely. So we did a strategic review, which I led, and we came to the conclusion that Nippon Life would be a great steward of this business—that we could be in business with them on an 80-20 basis, with Nippon Life being the 80 per cent. So we put up 20 per cent of the capital for the business, which we were very happy to do. But putting up 100 per cent of the capital was too much for us—and, as I said, Nippon Life brings all these benefits.

So the consumer in Australia wins from this. In the areas of pricing, product innovation, claims transformation and systems development, what Nippon Life is bringing to MLC Life augurs well. MLC Life wants to be the No. 1 life insurer in Australia, so we want to bear the scrutiny of all your questions. We will not get to No. 1 unless we are doing the right thing by customers and building trust and confidence with them.

Mr Miller: Whether it be shareholder owned or mutual, the balance sheet strength that sits around life insurance companies is vitally important. If you think back through the eighties and nineties—there were a lot of both shareholder and mutual brand life insurance companies that are no longer there. They have been amalgamated over time. That includes the Legal and General brand, the Prudential brand and so on. It is about the balance sheet. That is my first point.

My second point is that, in lots of ways, life insurance is the epitome of the mutual. Life insurance, at its very heart, is based on the fact that a lot of people will pay a premium knowing full well that only some people will get paid out of that. That is what life insurance is about. I think setting it up inside a mutual like Nippon—I think that is a good home for it.

CHAIR: With your group insurance, do you provide it through any of the super industry funds?

Mr Hagger: Very little. We are proud to be the group insurer for QuickSuper, but MLC has not been in a position to pursue business in the group market for the very big industry super funds. Nippon Life needed to make significant system upgrades before that could happen. I think we will see that over time. While I was overseeing the business, it was not an area that was pursued strongly. That is why in my opening comments I pointed to the fact that 65 per cent of the business of MLC Life is through retail advice.

Mr VAN MANEN: You said before that MLC Super is one of the largest super funds in the country. Is the insurance written through that written on a group life basis, or is it written on an individual underwritten basis?

Mr Hagger: It is written on a group life basis.

Mr Miller: Mr Hagger is correct. For our corporate service line, it is on a group life basis. In the retail superannuation space, which is also a segment of that overall super fund, there could be retail insurance connected to a super fund as part of that.

Senator O'NEILL: You would probably be aware of the article in *The Australian* by Michael Roddan about APRA and the decades of neglect by insurers?

Mr Hagger: Yes, I read that article. I do not have it in front of me. I can call it up if you like.

Senator O'NEILL: There might be a copy here for you. We did hear something about this from APRA in one of our hearings—I think the one in Sydney. They talked about the prudential risk that was not yet fully formed, but they were raising flags of concern about the back office capacity of the insurance industry. MLC Life rates a mention in Mr Roddan's commentary:

Another insurer, understood to be MLC Life, stores customer records on microfiche, a dated technology that was formerly used to store photographs of newspaper pages. One super fund has had to source a spare part on eBay to keep one of its legacy systems operational …

Are you aware of the situation with MLC Life?

Mr Hagger: Yes, I am.

Senator O'NEILL: Were you actually using microfiche?

Mr Hagger: We still use microfiche. Let me explain. Firstly, we have 1.4 million customers in MLC Life, so I do not want the committee to be under the impression that the entire business is run via microfiche in the back office.

Senator O'NEILL: I am feeling very relieved, Mr Hagger!

Mr Hagger: You are entitled to feel relieved. Mr van Manen was talking about legacy products, and we have some traditional legacy products—a small number of them—going back to the 1980s. Actually, under record-keeping rules, we have not needed to keep records, but we keep, I think, something like two boxes of records in one office in Australia for those very old products so that if somebody needs to reference them we have still got those records there.

The core point of this article is something that we entirely believe in, and that is that APRA is saying, 'You need to spend money on your systems.' When we sold the business to Nippon Life Insurance Company, we agreed at the point of sale with Nippon Life—it was one of the key things they wanted and a key thing we agreed to—that there would be a bucket of money—and it is a big bucket of money, around $400 million—set aside to improve the systems of MLC Life to provide the latest state-of-the-art technology. This project, which is something that I see as a board member on a monthly basis, is upgrading the systems of MLC Life in a great way. This will be great for customers.

Senator O'NEILL: Did they think that the business was a renovator's dream when they found out about this?

Mr Hagger: I do not know whether you would go that far. A microfiche would certainly make it that. If the microfiche had been for all 1.4 million customers, it would probably be even beyond that, but the systems work as they are. We have very high respect for APRA member Geoff Summerhayes. His point is that we know the insurance industry is not making a lot of money. You have may have seen that the life insurance industry has had another fall in profits this year. The regulator quite rightly is saying, 'Just because you are making less profit, don't stop investing in your systems for the benefit of your customers.'

Senator O'NEILL: Can I take you to that. With there being less profit, what sort of changes in sales practices does that bring about? Is there pressure on culture to sell more when there are such significant losses as are written about by Michael Roddan in the article today? What pressure does that apply?

Mr Hagger: When I first came in as Group Executive of NAB Wealth, in my first half-year of overseeing that business we reported a loss in the life insurance business. That may have been the first loss—I am not sure whether it was or not, but it may have been—in 130 years of MLC Life insurance. My instructions to the team were that we needed to improve the business the right way for our customers and give very good customer outcomes.

Senator O'NEILL: What year was that, Mr Hagger?

Mr Hagger: It was 2013. If you compare the amount that we paid in claims, for example, in 2016, when we sold the business, versus 2013, we paid $150 million more in claims. Our sales practices were stronger in 2016 and our advice practices were stronger in 2016. We took the approach that the best way to turn the business around was to act for customers, and it worked.

Senator O'NEILL: I appreciate the fullness of your answers, Mr Hagger, but I have a lot that I would like to get on the record today, in addition to ones that I will probably put on notice.

Mr Hagger: I will try to keep my answers short, then.

Senator O'NEILL: Thank you very much. I appreciate you trying to answer fully, but I have a few that I want to get through. You said you instituted these changes in 2013, but in 2016 NAB was going to conduct a review into denied claims dating back to January 2014, particularly in relation to critical illness including heart attacks. What is the status of that review?

Mr Hagger: I believe that review is well progressed. From time to time we get updates at the board, and as I understand it—

Senator O'NEILL: When did it start? When did you commence the review?

Mr Hagger: Can I take on notice the exact dates?

Senator O'NEILL: Sure. And you are getting updates on that review?

Mr Hagger: Yes.

Senator O'NEILL: At board meetings—how frequently?

Mr Hagger: The board meets monthly, so we get reviews from time to time.

Senator O'NEILL: When do you expect it to be completed?

Mr Hagger: From memory—I should take it on notice—I think it is of the order of August this year.

Senator O'NEILL: Okay. So it has been going for quite some time. Have recommendations been acted on in the interim or are your waiting for recommendations followed by action?

Mr Hagger: Recommendations are acted upon in the interim. For example, where Mr Arnold has found anything where he believes a claim should have been paid or a higher amount should have been paid, obviously that gets actioned. You do not wait till the end of the report season to action those things.

Senator O'NEILL: How many claims have been identified and responded to so far?

Mr Hagger: I will take that on notice to give you the exact amount.

Senator O'NEILL: With regard to sales practices, which I was just speaking briefly with you about, in 2015 it was reported that NAB removed some financial advisers due to their practices—

Mr Hagger: Yes.

Senator O'NEILL: which was after 2013, when you instituted change. So, two years later, they were still there. What work is currently being done to prevent conflicts of interest and inappropriate advice and practices by financial advisers and others who sell insurance?

Mr Miller: There are a range of things in there. I have already covered off the fact that we have a broad approved list, and so an adviser can use a range of companies. Clearly we have been really supportive.

Senator O'NEILL: I wonder if you could take on notice to give us the detail of what you have got there, because there is a lot of information that is coming verbally. I am trying to understand how distinctive that is as a suite of options that are available with NAB that might not be replicated somewhere else. I appreciate that they could come from a whole lot of places, but is this a NAB suite that does not have much cross-over into other areas, or is it extremely varied and the same products would be found in a range of major institutions?

Mr Miller: They certainly would be your common products of life insurance, income protection and critical illness, but we have nine different suppliers of those, and different suppliers will have sets of different philosophies of underwriting our claims, different premium rates, and, certainly, acceptance of different occupations and so on because of the way they build up their suites.

Senator O'NEILL: Are those nine primarily associated with MLC and NAB or do they interact with all the other majors?

Mr Miller: No. Eight of those are owned and controlled by other life insurance companies.

Senator O'NEILL: Okay. Thank you.

Mr Miller: Is that what you were asking, Senator?

Senator O'NEILL: Yes, that was one of the things I wanted to know.

Mr Miller: Those eight have probably been in front of you over various times through these sorts of hearings. We use a range of other life insurers. So, when I say there are nine, MLC Life is one, and there are eight other insurance providers on our approved list, with a range of their products underneath so that advisers can have a wide range. The comment I was going to make is that there are a few key things in professional standards across the business. There are things we have been doing internally in terms of our practices, but, at the same time, the best interests of FOFA, ABA reference-checking protocols and those sorts of things are really important parts of lifting the professional standards of advice. That is really important because that leads to much better sales practices and much better outcomes for customers.

Senator O'NEILL: Can you take on notice how many new RG 146 qualified people you have taken on in the last financial year—

Mr Miller: Sure.

Senator O'NEILL: because I think there is continuing concern about the nature of that qualification.

Mr Miller: Sure. I can take that on notice.

Senator O'NEILL: It is a very big problem. Is there a need for an independent tribunal to consider life insurance policies with a focus on benefits rather than premiums?

Mr Hagger: Can you please explain a little more what is on your mind?

Senator O'NEILL: The focus is often on premiums and the cost of insurance rather than on the benefits and the outcomes for consumers, and there are certainly conversations about the difficulty of people easily accessing places to have their complaints resolved. So is it time for an independent tribunal?

Mr Miller: Are you talking about FOS and those sorts of approaches?

Senator O'NEILL: Yes. I guess another question—

Mr Miller: FOS is certainly one area today, as the Superannuation Complaints Tribunal would be if your insurance is in super and so on. So there are some avenues today that people would go to if they felt that they were aggrieved by what they were provided in terms of advice and ultimately the process of life insurance.

Mr Hagger: Can I just make an aside to this. I know the core of your question is tribunals, but actually the benefits of insurance go beyond the claims process. That is one of the areas that we are focused on—the broader customer experience including, for example, on some products access to best doctors—which is something that is industry leading for us.

Senator O'NEILL: I will probably put on notice a fuller question. Excluding remuneration associated with sales, are there other remuneration arrangements that may create conflicts of interests—for example, case managers, medical examiners, investigators or superannuation trustees?

Mr Miller: No.

Mr Hagger: Not that I am aware of, but perhaps we could take that on notice.

Senator O'NEILL: For the multiple policies with a single payout—that was a bit of a shock for me to find out about—are you aware of that?

Mr Hagger: Are you talking about if somebody takes a policy with life insurers A, B and C and then the one event happens and they claim with A, B and C?

Senator O'NEILL: Yes.

Mr Miller: With the offset provision?

Senator O'NEILL: Yes.

Mr Miller: I am aware of the offset provision. I would not say that I could talk eloquently about the range of carriers and their various offset provisions, but I am aware of the provision.

Senator O'NEILL: Does your business engage in that offset provision with other businesses? Do you negotiate?

Mr Miller: I would have to take that on notice to understand what MLC Life do in relation to that and how that is worked.

Senator O'NEILL: I would like to know how it is provided—

Mr Miller: It is probably worked around a salary continuance or income protection component where it is predominantly seen. On the nature of how it is applied, I would have to take it on notice to give you the details.

Senator O'NEILL: I would like to perhaps understand who decides which one pays.

CHAIR: How do they know who is insured and who is on it? How would the other insurers know that you have another insurance policy?

Senator O'NEILL: Exactly. If they have had multiple policies and the claim is successful, do they get a refund from the agencies that have not paid?

Mr Miller: We can supply some information on that. I think we will just wait to do that.

Senator O'NEILL: It seems like a pretty good rort to me to be able to keep that money and not pay out on it. It is of concern.

CHAIR: Can Senator Williams jump in here and ask a couple of questions, please.

Senator O'NEILL: Sure, and I will hand over to Senator Ketter as well.

Senator WILLIAMS: Mr Hagger, in 2016, NAB was to conduct a review into denied claims dating back to January 2014, particularly into critical illness, heart attack et cetera. What is the status of this review?

Mr Hagger: That is the same review that the deputy chair was talking about. It is on foot. I believe it will be completed around August. I have taken that on notice to give more procedure.

Senator WILLIAMS: Can you take this on notice: I want a percentage ratio of the claims that are paid by group insurance, direct insurance and retail insurance. I do not want you to go back the last 50 years and count up how many claims you have paid et cetera. I do not want to put you through that work, but I would like the committee to have an idea of the percentage of claims paid—retail, where you have a financial planner involved and for direct and group. Is that a problem?

Mr Hagger: No. We can provide that. I can give you some estimates to give you—

Senator WILLIAMS: Estimates will be fine. Give us estimates, please.

Mr Hagger: Typically, group insurance tends to be around 70 to 80 per cent. It has the highest claims ratio, because it does not have the cost of the advice and various other things inside it. The claims rate for advised products—

Senator WILLIAMS: You have lost me there. With group insurance, 70 to 80 per cent pay on the claims, but they do not have the cost of the adviser. What would the cost of an adviser have to do with whether you approve the claim?

Mr Hagger: What I mean is that, when you run a group insurance business, you present to a trustee and provide cover to a large group of people.

Senator WILLIAMS: You do the deal with a super fund or whatever.

Mr Hagger: Yes.

Senator WILLIAMS: You have done a deal and they have covered everyone, whether they are smokers or drunks.

Mr Hagger: Yes. It is sort of an economies of scale approach. Therefore, you pass the benefit of that back to the consumer.

Senator WILLIAMS: Okay.

Mr Hagger: That tends to be 70 to 80 per cent.

Senator WILLIAMS: Right. What about with retail insurance?

Mr Hagger: Retail insurance tends to be around the 45 to 55 per cent range—

Senator WILLIAMS: Approved?

Mr Hagger: No. I need to make sure I am being precise.

Senator WILLIAMS: What I am saying is I want—

Mr Hagger: The percentage of claims to premiums—

Senator WILLIAMS: the percentage of claims approved.

Mr Hagger: That is very different.

Senator WILLIAMS: Yes.

Mr Hagger: Now I understand. We will give you that data, but what you will find is—

Senator WILLIAMS: If there are 100 claims from group insurance, 100 claims from direct insurance and 100 claims from retail—

Mr Hagger: I understand—

Senator WILLIAMS: what are the percentages that are granted—that are approved?

Mr Hagger: I will give you the data. I would be horrified if my earlier comments were applied to that question.

Senator WILLIAMS: That is what I was worried about—35 per cent was retail.

Mr Hagger: Do not have any fears.

Senator WILLIAMS: Crikey, they were getting bad advice! We will delete that bit of history in this report.

Mr Hagger: I think our internally disputed claims overall are four per cent, and we have less than one per cent externally disputed. I will give you the split between the three but we are already at the 96 per cent of claims approved, overall.

Senator WILLIAMS: Good. I am glad I clarified that.

Mr Hagger: I am glad we did too.

Senator KETTER: You would recall that earlier this year your company was questioned about financial adviser Mr Shylesh Sriranjan, a financial adviser who had cheated on the compliance exams.

Mr Hagger: Yes.

Senator KETTER: Could you tell us what steps you have taken to ensure that this type of activity does not continue?

Mr Miller: We put a note out to all of our advisers in NAB financial planning and across our network, once again, explaining and enforcing the ethics that we have as an organisation and the culture that we want to uphold. In addition to that, at a broader level, what we have done over the last year is ask all of our advisers to be a member of a professional association. We think that is really important because, with that, comes code of practice and ethics and so on. So we have reinforced that. As an aside to that, we also wrote to all his customers.

Senator KETTER: I note that gentleman was expelled from the Financial Planning Association as a result of that.

Mr Miller: That is my belief; I will have to take that on notice.

Senator KETTER: Given these relatively recent developments, do you support the Life Insurance Code of Practice being registered with ASIC's regulatory guidance 183?

Mr Hagger: I was involved, in my time, when I was on the board of the FSC, in the development of the code, and we talked to various consumer groups. This is a several-step process. Step No. 1 was to establish the code. That is what has happened so far. You are talking about the next step, which is to make it an enforceable code of conduct. Now that we have done the first step, the next thing going on is that the parties that are not subject to the code—this is, in particular, the superannuation industry—are now saying, 'We're happy to explore what to do about the life insurance code so that it applies to us as well.' They will have their views and that process will go on. Once that occurs, the industry is in a situation where it may be the right thing to do, to take that next step to be an enforceable code.

I know at the beginning we had discussions with ASIC about this. We also talked to a number of consumer groups, some of whom have addressed this committee. We believe the right steps have been taken so far. For MLC Life and NAB, we are both subject to that code, and we will comply with that code.

Senator KETTER: My question is: do you support, at some point, that code of practice being registered with ASIC?

Mr Hagger: Yes, we do. The precursor to that is the broader superannuation industry. Once the whole industry is across it and in the gates, so to speak, if ASIC wants to register it and the various things that go along with that, then that will be a natural step when that moment comes.

Senator O'NEILL: With regard to the use of genetic information—there has been quite a lag since the last report and we will be taking some evidence around that today—how is genetic information used by insurers, particularly when making underwriting and policy decisions? And is there sufficient expertise to understand genetic data, its statistical value and significance, and whether the data has been validated through laboratories accredited by the National Association of Testing Authorities?

Mr Hagger: Firstly, I know later today you are hearing from Simon Longstaff, and I think that will be a very interesting discussion on the ethics of genome testing et cetera. Where medical science is up to is not where the industry has gone to. It raises so many ethical issues from so many different angles, but I think it is very good for the committee to look at that. I have had discussions myself with Dr Longstaff on these issues, because we want to strike the right note ethically.

Senator O'NEILL: Can I say, Mr Hagger—not to hold you personally to account for decisions that have been made perhaps in other entities that you are associated with—our discussion earlier about people's private records with their doctors being available in toto is the first ethical consideration that I think needs to be looked at, because it is happening right now. Given that it is not of immediate concern to you, I really worry about unleashing the capacity of genetic testing as a tool for actuarial accounting in the industry.

Mr Hagger: Firstly, the fact that you raised the matter earlier and I took it on notice was for me to explore it more. I do not want you to leave this discussion thinking it is not a matter of concern to me. You have raised it; I have taken it on notice, and I will follow through with that.

Senator O'NEILL: I appreciate that very much, Mr Hagger. Thank you.

Mr Hagger: In relation to genome testing, that will be something that the industry will need to look at over time to work out what the right approach is. As I mentioned earlier, life insurance is a very dynamic industry. But we at NAB and MLC Life are very alive to the ethical dilemmas involved in that, so we would tread very, very cautiously in relation to those matters—and we would be guided by people like Dr Longstaff, who we respect highly.

Senator O'NEILL: What is your position at this point in time with regard to Australia having legislation similar to that already in place in many countries around the world that actually prohibits the use of genetic information by insurers when setting premium levels?

Mr Hagger: I am not expert enough on that situation to have a personal position, but I am happy to take that on notice.

Senator O'NEILL: Thank you very much. Are you aware if genetic testing and genetic profiling are being used at the moment for actuarial modelling within the MLC?

Mr Hagger: No; I am not aware.

Senator O'NEILL: Does that mean you do not know, or you think it is not happening?

Mr Hagger: I think your question was whether I am aware, and I am not aware. But, if you would like me to check, I am happy to check.

Senator O'NEILL: Yes. Could you find out what is going on in that space. If there is any use of that, are customers being made aware of that?

Mr Hagger: Because I do not know the answer to the first part—

Senator O'NEILL: Yes; I know. It is a corollary.

Mr Hagger: I will take it on notice.

Senator O'NEILL: Fantastic. With regard to unfair contracts, what benefits and challenges may arise if unfair contract terms in legislation were extended to cover all sectors of the life insurance industry?

Mr Hagger: I think the main challenge that would occur is that the legislative framework, as I understand it, is through the Insurance Contracts Act, which has a number of protections in it. I am not a lawyer, so I do not know a lot about that, but we do experience challenges in financial services just by the different rafts of legislation that apply. I think the Insurance Contracts Act has been the focus of attention, and that is the area that the industry responds to. I probably do not need to say anything more than that.

Senator O'NEILL: If you could take on notice to give us a more formal position about that, I would appreciate it. In terms of minimum standards, what are the benefits and challenges in establishing a set of minimum standards for the life insurance sector? Do you see the code, which is voluntary, as achieving that, or is that only one step in the right direction?

Mr Hagger: As I mentioned earlier, it is a step in the right direction. There are minimum standards here. I mentioned today—and had to clarify, really, to make the point—that we support standard medical definitions, which is a key area. There are minimum standards of customer service enshrined in the code, and we intend to meet them. There is a natural hedge here, which is: the more we can do for our customers, in our experience, the better that is for our business, and it makes us a long-term business that has the trust and confidence of customers. That is our compass in this.

Senator O'NEILL: Is that perhaps an explanation of the shift to a mutual company rather than a short-term, bank-owned business?

Mr Hagger: No. It is not the explanation of it. The shift is due to different capital treatments, as I mentioned earlier. The philosophies of NAB and Nippon Life around customers are very much alike.

Senator O'NEILL: But they have a lot more money to put into making that real.

Mr Hagger: They have varied patient capital there. They are innovative. They are strong. They will be fantastic owners of MLC Life.

Senator O'NEILL: I do have a number of other question that I will ask the secretariat to send on notice.

Mr Hagger: Sure.

Senator O'NEILL: Thank you very much for your time today.

Mr Hagger: Thanks.

CHAIR: As we have run out of time, I thank the witnesses for attending today. A copy of any questions that have been given to you or taken on notice will be provided to you by the secretariat. Those answers should be

provided back to the secretariat by 9 June. Again, thank you for attending the hearings today and for your evidence.

Proceedings suspended from 10:20 to 10:30

ENTHOVEN, Mr Richard, Executive Chairman, Greenstone Pty Ltd

GROBLER, Mr Brenard, Chief Operating Officer, Greenstone Pty Ltd

CHAIR: I reopen this hearing on life insurance. The committee welcomes Greenstone Pty Ltd. I remind committee members and witnesses that, while this is a public hearing, care should be taken to protect the privacy of individuals and that arguments should be made without naming individuals. I now invite you to make a short opening statement, and at the conclusion of your remarks I will invite members of the committee to put questions to you.

Mr Enthoven: Good morning and thank you for the opportunity to appear before the committee today. We believe this is an important forum, and as an associate member of the Financial Services Council we were involved in their submission to you.

Greenstone Financial Services Pty Ltd is an Australian company employing over 500 people, based in north-west Sydney. We provide a suite of insurance products, under an Australian Financial Services licence, directly to customers—we have since 2007—primarily under the Real Insurance brand. Greenstone acts as an agent of Hannover Life Re of Australasia Ltd, who have operated in Australia since 1958. Unlike Greenstone, Hannover is an APRA-authorised life insurance company who issues the Real Insurance policies. Greenstone handles the following functions in respect of those policies: the promotion and marketing of the Real Insurance branded policies, the underwriting process for all applicants and the referral to Hannover of cases where further underwriting is required, the issuance and distribution of policy documents, collection of premiums, ongoing policy administration, the management of policy cancellations and lapses, and the management of claims.

We proactively engage with Hannover Life Re when they set the underwriting criteria that apply at the time of application to simplify the process and enhance clarity for customers. Importantly, as customers are acquiring their policy under our brand, we have a vested long-term interest to ensure they receive satisfactory outcomes. As such, we take great pride in the fact that Real Insurance has won the Roy Morgan Customer Satisfaction Award in the life insurance category in three of the past five years.

As the committee is aware, there are three primary distribution channels for life insurance in Australia: advisers, group and direct. Greenstone believes that all three channels play a valuable and important role in making life insurance—which is a critical component of the social safety net—easy for the community to access. However, Greenstone only participates in the direct channel. The direct channel represents an important mechanism for individuals to acquire life insurance in a convenient and affordable fashion. Within the direct channel, there are a number of different operating models and product types, so I would like to take this opportunity to draw the committee's attention to a few salient points.

Greenstone underwrites the Real Insurance policies at the point of application. Indeed, we conduct a full tele-underwriting process prior to any sale being made and not at claims time. As a result, our life insurance policies have only one single exclusion—namely, suicide in the first 13 months.

Greenstone policies have no additional cost beyond the premiums agreed with the customer. In other words, our customers are not required to pay any additional policy or advice fees, nor are they required to incur the costs of a medical exam or other specialist fees.

We agree with ASIC's findings in report 498 that there is no consistency in claims reporting, and, as such, comparing claims data is complicated. Additionally, Greenstone believes that any comparison that does not consider policy tenure—in other words, how long a customer has been a policyholder—is flawed. For example, it is widely accepted within the industry that a death claim in the first year of a policy is more likely to be declined for breach of duty of disclosure than a death claim in the fifth or the 10th year of a policy, for example. As a result, unless claims denial rates are compared by policy tenure, younger or faster growing companies will be unfairly prejudiced.

Greenstone is embracing the Life Insurance Code of Practice as one of the most important steps forward for our industry in recent times. We are wholly supportive of the increased disclosure obligations and the customer protections—in particular, the codifying of financial hardship obligations. We would like to acknowledge the importance of this inquiry in that it will help shape the future of the life insurance sector for both underwriters and their agents such as Greenstone, ensuring that Australians are supported at a time when they are most in need.

In closing, we at Greenstone take very seriously our responsibilities to customers and their families. The manner in which we service our customers is paramount to the success of our operation. We shall continue to listen to their needs and innovate to enhance the quality of their lives. Thank you.

Senator O'NEILL: Before we hear from Mr Grobler, is there any way we could get a copy of your opening statement?

Mr Enthoven: I would be delighted to share it.

Senator O'NEILL: Thank you.

Senator WILLIAMS: Mr Enthoven and Mr Grobler, thank you for your presence here. When I go to your website about Real Life Insurance, it says:

- Apply over the phone in a single call with no medicals or blood tests
- You can keep your cover for life—

et cetera. I would get a call from one of your staff, obviously. What sort of questions do they ask me?

Mr Grobler: It is actually quite a lengthy underwriting process. If you answer no to every up-front question, it will probably be 34 questions you need to answer, but there is an opportunity for you to end up answering more than 100 questions, depending on the responses you give to the initial questions. All our questions require a clear yes or no answer in response, and it would include all aspects of your health and lifestyle. We fully underwrite hazardous pursuits, so we will ask the questions: 'Do you currently participate in hang-gliding, scuba diving et cetera?' We underwrite family history at point of application. We underwrite all health issues and residency questions at point of application.

Senator WILLIAMS: So it is all underwritten at the conclusion of questions?

Mr Grobler: Yes, and we will only capture a yes or a no as a response. That could then result in acceptance at standard rates, or potentially a loaded premium based on your responses, or potentially a decline.

Mr Enthoven: To be clear, that is why we are able to offer a policy with one single exclusion, which is suicide in the first 13 months.

Senator WILLIAMS: When your staffer calls me and I say, 'Look, I've got another policy,' do they give me any advice on my previous policy?

Mr Grobler: It is really important to put on record that we do not offer any form of advice. We operate under a general advice licence. In most instances, customers or potential customers will contact us. They will respond to one of our promotions, whether on the radio or on Google or the websites, and they will call into our business in Sydney, or there is an option on our website to request a call or a quote, and we will then call them at home or at work.

Senator WILLIAMS: What steps do you take to ensure that your staff do not give any advice? Do you monitor that et cetera? What steps do you take in that regard?

Mr Grobler: Importantly, every single call in our business is recorded. We have an independent quality assurance department that sits removed from the sales floor, and they will assess sales lines to ensure that there was adherence to our strict compliance regime.

Senator WILLIAMS: When it comes to claims, what is your percentage of claims approved?

Mr Grobler: Before I respond to that question, it is important to put in context the age of our book, which is quite young. We only really started distributing life insurance products since 2007, so we are not expecting an influx of claims yet on such a young book, numbering only 160,000 policies at this date. But measured over the last 12 months—I asked our claims department to give me some statistics—our decline rate was 2.1 per cent so only 33 claims were declined. Invariably all of those declines were on the basis of fraudulent non-disclosure at the point of application.

Senator O'NEILL: Could you repeat that last sentence. I did not quite catch it.

Mr Grobler: As Richard mentioned in his opening statement, we only had this one single exclusion on our life policies—that is, suicide in the first 13 months. Should a claim be declined, it would be on the back of dishonest disclosure at the point of application. We read out the duty of disclosure to the customer when they go through the underwriting question there at the point of application and they are made aware that their response is what the insurer will rely on them to offer them cover. If they then dishonestly or fraudulently non-disclose a specific health issue that they might have had—it might be a question where we ask them: have you ever been diagnosed with cancer—if they knowingly answer 'no' whereas they are currently, for instance, undergoing chemo treatment, that would be a fraudulent non-disclosure. In those instances, the insurer would deny that claim and

return all their premiums paid to date and void the policy because they would have a point in case to say, 'Had we known that condition existed at the point of application, we would never have offered cover.'

Senator WILLIAMS: Using the example you gave where somebody said, 'I have never had cancer,' when in fact they have had cancer and are getting treatment with chemotherapy or treatment for a particular cancer, when someone puts in a claim, you go right back through their medical history. Is that correct?

Mr Grobler: No. I do not want to speak on behalf—

Senator WILLIAMS: The question is this: how do you know that they are being dishonest? How do you go back through their medical history? When somebody says on their policy, 'No, I have never had cancer,' when in fact they are being treated for cancer, how do you find that out? Do you go through the medical history, the books, the records of the client?

Mr Grobler: We will not because we do not assess the claims. Our insurer will do that. But we will forward them a record of the call at the point of application, the record that we keep.

Senator WILLIAMS: Your insurer is who?

Mr Grobler: Our insurer for the Real Insurance is Hannover Life Re of Australasia.

Senator WILLIAMS: So they do all the research on the medical history?

Mr Grobler: They assess all claims.

Senator WILLIAMS: Do you have independent medical examiners?

Mr Grobler: I would not want to speak on their behalf but I know that in the terms of conditions of the policy they have the right to use their own medical practitioner as well.

Senator WILLIAMS: So you do not know a lot about how they have access to my medical records if I was putting a claim in? You do not handle that side of it?

Mr Enthoven: As my opening remarks made clear, we are acting as an agent of theirs. We specifically do not have a licence and therefore do not make claims decisions. That said, as was not mentioned, we have done a review of all of the claims that have been submitted; 2.1 per cent have been declined and that represents an absolute number of 33 claims.

Senator WILLIAMS: ASIC did an inquiry into you about your funeral insurance. Is that correct?

Mr Enthoven: That is correct.

Senator WILLIAMS: You have made changes to your funeral insurance. For example, premium payments must stop when someone turns 80 years of age.

Mr Grobler: The funeral product has three integrated guarantees. The first one is your premium will never increase as long as you hold the policy. The second one is your premium will actually decrease every five years by five per cent and that is to assist policy holders in old age when their income deteriorates. And the third one is you will never get a payment less than your premiums paid. So in no instance can a policy holder pay more than what they will get out when they claim. By default, every single claim is paid except if it is due to natural causes in the first 12 months. That is the only exclusion on the funeral product. If you pass away in the first 12 months due to natural causes, we decline the claim—or Hannover Life Re would decline the claim—and return premiums paid. Thereafter, if death is due to any causes, there is a guaranteed payout. There is absolutely no underwriting on that product but there are also no exclusions on that product.

Senator WILLIAMS: Does Real Insurance have retail insurance as well, or do you do all direct insurance?

Mr Grobler: Only direct insurance.

Senator WILLIAMS: Only direct. You do not do any group insurance, of course?

Mr Grobler: No.

Senator O'NEILL: I have a number of general questions, but I want to go to the evidence you gave this morning. You said the acceptance of somebody's request for insurance could go three ways: they might be offered insurance at a standard rate, or with a loaded premium, or it might be denied. What is the quantum of business that you wrote in the last financial year?

Mr Grobler: The number of policies?

Senator O'NEILL: Yes.

Mr Grobler: On life insurance, it would be circa 25,000 to 28,000—roughly 2,000 a month.

Senator O'NEILL: Of that 25,000, how many people would be offered a standard rate?

Mr Grobler: Typically—and I can take it on notice for the exact number—it would be in the vicinity of 70 per cent of all applicants.

Senator O'NEILL: Seventy?

Mr Grobler: Seven zero, yes

Mr Enthoven: Can I just clarify that: if we take it as a pool of applicants, 70 per cent are approved on the standard rates, roughly 10 per cent are approved with a loading—

Senator O'NEILL: Premium?

Mr Enthoven: Premium, or a condition excluded; in other words, if somebody says, 'I have a specific condition,' then the policy may exclude that specific condition. And finally, circa 20 per cent we are not able to insure.

Senator O'NEILL: What would be excluded or covered with a loaded premium?

Mr Grobler: If it is a premium that is only loaded, there are no additional exclusions; it is still only the suicide exclusion. But if it is—

Senator O'NEILL: Sorry; how you determine the additional payments of the loaded premium?

Mr Grobler: There are various instances where that could be triggered in the underwriting rules engine, it might be based on weight and height, it might be based on certain health issues—if you have been treated for depression more than once, that could trigger a loading.

Senator O'NEILL: So you get one get-out-of-jail-free card for depression?

Mr Grobler: That was per exemple, I can take on notice the exact underwriting rules on mental health.

Senator O'NEILL: I would appreciate that, because that is an area of significant concern.

Mr Enthoven: I would like to clarify, though, that we do offer cover for certain individuals with certain mental health conditions, and there are no mental health condition exclusions in our policy.

Senator O'NEILL: What are the ones that you would cover and that would still secure a standard offer?

Mr Enthoven: We will take it on notice to give you detailed map of who would and would not be covered, and who would get a loading.

Senator O'NEILL: Okay. So are there some people who would disclose a mental health condition who would still be offered a standard contract?

Mr Grobler: Yes.

Senator O'NEILL: So some would get a standard contract?

Mr Grobler: Yes.

Senator O'NEILL: I will not make assumptions, I will wait to hear from you in more detail. For the 10 per cent who receive a loaded premium, what is the scale of the loading?

Mr Grobler: It could be as low as 25 per cent additional; it could be as high as 150 per cent.

Senator O'NEILL: So the lowest loading is 25 per cent, and the highest is 150 per cent.

Mr Grobler: Yes. There could be multiple loadings attached. It could be, for a certain disclosure there is 50 per cent and then for another disclosure there is an additional 50 per cent. But there is a limit on the maximum loading a policy may get.

Senator O'NEILL: How much is the maximum loading?

Mr Grobler: I can take that on notice but, from memory, it is 250 per cent, and more than that is not allowed.

Mr Enthoven: To be clear, we do not set those loadings. That is set by our insurance company partners.

Senator O'NEILL: Is that based on their actuarial—

Mr Enthoven: Based on their actuarial analysis of the risk that that condition imparts to the policy contract.

Senator O'NEILL: I would like to come back to talking about the genetic markers a little bit later.

CHAIR: When you go back to someone with a loaded premium, do you tell them the reasons why and give them a full explanation?

Mr Enthoven: Correct.

CHAIR: I do not know if you are keeping records of it, but how many times would they reject or accept the contract, or the insurance policy?

Mr Grobler: It could play out in two ways. They might say, 'That's unaffordable.' For instance, let us say it is a 50 per cent loading. They might say then, 'The premium is unaffordable for me,' and end up taking a lower sum insured or a lower cover, or they might say, 'Let me take this under consideration,' and they at that point might take up the policy. Or they might take up the policy and utilise our 30-day cooling-off period and, once they have all the documentation to hand and they fully understand the terms and conditions of the policy, they might then elect to keep it, or they can cancel the policy in that 30-day cooling-off period, and we will return their premiums to them.

CHAIR: Surely, if you are underwriting at the point of application, that would be a point of difference against most of your online insurers—and you do not scream about it on the front of your website?

Mr Grobler: The last thing we would ever want to happen to one of our customers is for them to take up one of our products, pay a premium diligently for 10 years and then understand they never had cover. That is on purpose—

CHAIR: I understand that. I am saying it is a great point of difference. Why don't you use it?

Mr Grobler: We work with the insurers to come up with an underwriting rules engine that ensures the customers get value for their money when they buy it. That is why we opted for not having any exclusions, not even on hazardous pursuits. Some products out there will exclude hang gliding or scuba diving—

Senator O'NEILL: As a mother, I completely agree!

Mr Grobler: We prefer to underwrite at point of application for that, and that then means that, if you take up those hobbies or activities at a later stage in your life, you are covered, because a lot of customers do not remember the exclusions in a policy.

Senator O'NEILL: That is a very honest reflection. Insurance is something people do not think about. They do it, they get it done and it is in the bottom drawer. They want they reassurance. It is set and forget, and they are done.

Mr Grobler: Agreed.

Mr Enthoven: I would just like to say that I think there have been some self-interested distortions of the practices of the direct life industry that have been put into the media. We are not the only direct life company that has this approach, although certainly some of our competitors would use things like pre-existing conditions exclusions, which in my view is a time bomb and will cause those insurers significant brand and reputation damage in the years to come.

CHAIR: I want to go back to Mr Grobler's statement about a 30-day cooling-off period. That sounds a bit like a car contract or a telephone contract or something.

Mr Grobler: Regulation actually stipulates that it should be at least a 14-day cooling-off period. We decided to offer our customers enough time so that, if at that point they want to canvass advice or compare our product against other products in the market, they have 30 days to do it. In those 30 days, they can contact our business again to ask for further clarification or they can contact us and cancel the product.

CHAIR: So after 30 days it would be a new application?

Mr Grobler: After 30 days it is a policy that they cannot cancel from inception. They can cancel it from that day going forward, but we will not return the premiums. Remember, they enjoyed the cover that the product provides from day one. If somebody holds our product for eight months and then decides they want to cancel, they have had the benefit of, let us say, $1 million worth of cover and they have paid eight months of premium of $50. That is a decision of the customer.

Senator O'NEILL: We got up to the loaded premium. You were going to provide me with some further details on notice. With the denial rate of 20 per cent, on what basis are those denials most commonly given?

Mr Grobler: Again, there are a few instances throughout the underwriting rules set where it would trigger a decline.

Senator O'NEILL: What are they?

Mr Grobler: Cancer would be a definite one, if you have been diagnosed with cancer.

Senator O'NEILL: That is an automatic exclusion?

Mr Grobler: Not an exclusion; it is actually a decline, so you cannot qualify for our cover. Another one would be if there were instances of heart attack or heart conditions. I can take it on notice and send through a full list. There are not many. There might be 10 or 12 decline points in an underwriting rules set that would include up

to 340 questions. The intention is to offer cover for as many as possible of our customers, but unfortunately we cannot accommodate everybody.

Senator O'NEILL: So the message remains get insurance while you are well.

Mr Enthoven: The risk pool obviously depends on young and old and healthy and unhealthy to balance, and if people are trying to acquire the policy when they are unhealthy that would have a significant impairment of the risk pool.

Senator O'NEILL: You said that you have a young book that just commenced in 2007 and I think you went on to make some points about the one year, five year and 10 year. Could you expand on the point you were making there.

Mr Enthoven: It is well known that duty of disclosure is a reason for declining claims. Obviously not the only one; the other one is policy terms and limits. But if we just look at that single one, once a customer has been a customer of yours for an extended period of time, the likelihood of relying on that significantly diminishes. So in the first year of a policy, you can imagine—

Senator O'NEILL: The facts that they have given you on their application are proven in the course of their ongoing health.

Mr Enthoven: Imagine the following scenario. You are diagnosed with cancer and you commence treatment for cancer. You are asked in our application process if you have ever been diagnosed with cancer and you say, 'No, I have not,' knowing full well that you have, and you die within the first year of the policy. That is a situation which would be deemed to be fraudulent misrepresentation; therefore the contract would be voided and the claim would be denied based on the duty of disclosure. But if you have been with us for 20 years—

Senator O'NEILL: Did I hear you say that the claim is denied, but you would also return any of the premiums in full that I have paid to you?

Mr Enthoven: Correct.

Mr Grobler: In full.

Mr Enthoven: Effectively, you have a free option.

Mr Grobler: The reason behind that is that the insurer would say, 'Had we known we would never have issued the contract; therefore we would have never collected a premium from you and therefore we return all your premiums.'

Senator O'NEILL: Okay. Of the 2.1 per cent that you have denied, are they all of that kind?

Mr Grobler: I looked at the detail of each and every one of those and all of them were on the back of nondisclosure, except one where it was an unfortunate situation of suicide in the first 13 months.

Senator O'NEILL: Right. What were the nondisclosures around?

Mr Grobler: I can take on notice and send you a complete list, but I can give you an example of one if you allow me a minute. I will not mention any names, but it came down to a review of the claimant's medical history. We found that she clearly failed to disclose her history of heart murmur, thrombocytopenia, emphysema and cirrhosis. So there were a number of instances where the question would have been specifically asked and she responded in the negative. All those are not conditions that somebody might not know about. You are either taking ongoing medication or you are seeing your medical practitioner on a monthly basis to treat the conditions. We limited our questions to ones that can be answered with a simple yes or no. So it is not open to interpretation. You either have been diagnosed with cancer or not.

Senator O'NEILL: In the course of your series of questions, have you ever considered asking for access to the patient's records through their doctors? Is there a tick-a-box option where they can say 'Yes, you can go and check with my doctor'? Have you ever instituted that?

Mr Grobler: No, never. But we will do it on the phone. If the automated rules engine cannot assess it, there might be an option to refer to the underwriters at Hannover Re. We will then collect two or three questions from customer not from medical practitioners and we will send that through to the insurer at Hannover Re in a real-time portal that we have between our business and the insurer. They will assess that and within 24 hours we will come back to the customer with the outcome. That might be a non-standard loading or it might be a decline, or it might be a specific exclusion. We will put that position to the applicant and if they will agree, they will take up the policy or not.

Senator O'NEILL: Do you think your business is robust enough and sustainable without you requiring access to customers' entire medical health records?

Mr Grobler: We will never require information from their medical records—because we fully underwrite at the application stage. Thereafter it is a matter for the insurer through their client processes.

Senator O'NEILL: There are sufficient disincentives, in your view, to prevent people from lying in that application process?

Mr Grobler: We make it very clear what their up-front duty of disclosure is. They should understand that, if they dishonestly disclose, their claims will not be valid. That does not prevent certain applicants from taking a chance and hoping they will get a payout on the back of fraudulent disclosure.

Mr Enthoven: To be absolutely clear: it needs to be fraudulent nondisclosure. If somebody made an error or was not aware of something, those are not good enough grounds on which to decline a claim.

Senator O'NEILL: Could you give me an example of that?

Mr Enthoven: Let us say we asked you, 'Have you been diagnosed with cancer?' At the time, you not been, but you actually did have cancer at the time—you were just unaware of it. You take out the policy and then, the next day, you go to see your doctor and your doctor says, 'You have cancer.' In those circumstances, you are covered for that condition. But, if you know you have cancer and you say no when you know you should have said yes—in that unfortunate situation your claim would be denied.

Senator O'NEILL: Have you had any disputes over denial of such claims? If so, how are they being mediated and what is their status?

Mr Grobler: We do have a few that have been escalated. Firstly they go through our internal dispute resolution process. Then they go through external resolution: the Financial Ombudsman Service. Over the past 12 months ending 17 April, we—when I say 'we', I am referring to the insurer, because invariably these disputes are raised against the insurer, but I see us as being in partnership—had four disputes that were escalated through FOS that were claims related.

Senator O'NEILL: Say that again—four disputes?

Mr Grobler: Four disputes that were claims related were escalated to FOS over the last 12 months.

Mr Enthoven: That is four where the ruling was in favour of the claimant. On four claims out of around 2,000, the beneficiaries elected to take their dispute to FOS and FOS found in their favour.

Senator O'NEILL: What do you take away from those encounters with FOS? What policy changes have you made as a result of those four disputes?

Mr Enthoven: Normally it would require us to engage with our life insurance partners to get them to make the questions more specific. FOS may have taken a view that somebody did not understand a question and that therefore, even though they were dishonest, it was not wilful.

Mr Grobler: Or blatant.

Mr Enthoven: As a result of that, we would spend time revising the questions to make them clearer.

Senator O'NEILL: How do you make that determination earlier in your process—so that it does not get to FOS—of what is wilful or blatant?

Mr Enthoven: Once again: 2.1 per cent of claims are denied. A small portion of those go to FOS. Of those that go to FOS, the majority of the findings are in favour of our life insurance partners. In a very small number of cases—four—the findings have been in favour of the claimant. That is, effectively, a difference of opinion between reasonable people about what is wilful misconduct versus factually inaccurate information that has not been provided wilfully. In my view that is actually the process working well, right? That is what the system is designed to do: give customers independent-free access to a third party to look over their specific situation. Clearly, we take great care not to allow it to happen but, when you are talking about thousands of claims, I think it is reasonable to expect that there potentially could be disputes over a small number. It is, in my view, appropriate that FOS would find across the industry sometimes in favour of the customer and sometimes in favour of the company, because that suggests that the dispute processes are working well.

Senator O'NEILL: Could I ask about income protection—do you provide that as well as direct insurance?

Mr Grobler: We do.

Senator O'NEILL: Could you give me some data around that.

Mr Grobler: It is a very small proportion. Predominantly, we focus on funeral insurance and life insurance, but we do offer underwritten income protection. Again, there is full underwriting at the application stage so customers know exactly what they are covered for, and there is no underwriting at the claims stage.

Senator O'NEILL: What is the size of your book and what are last year's stats?

Mr Grobler: I can give you an indication of the size of the book with a—

Mr Enthoven: Less than 10 per cent of our business is income protection.

Mr Grobler: It is really small. I do not seem to have that to hand—I thought I had; I think my indexing is not what it should be.

Senator O'NEILL: When all else fails, read the instructions somebody prepared for you.

CHAIR: You can take it on notice, if you like.

Mr Enthoven: We will take it on notice.

Mr Grobler: It is less than 10 per cent of our—in total we have 160,000 real insurance customers. I would be surprised if we have more than 16,000 income protection policies in force.

Senator O'NEILL: And how many claims, do you know, were made?

Mr Grobler: I do not have that data to hand. We have got life insurance claims statistics over the last 12 months to hand, but I can—

Senator O'NEILL: Do you want to give me those, or have we already done that?

Mr Grobler: I have given you life insurance. Again, we had 2.1 percent for clients, and that was a number of 33. It is such a small young book—

Senator O'NEILL: So 32 for dishonest disclosure and one for suicide.

Mr Grobler: Unfortunate suicide.

Mr Enthoven: And that is across all of our products. Given—

Senator O'NEILL: That includes income protection?

Mr Enthoven: Yes, and, given that income protection is a very small part of our portfolio, in absolute numbers, it will be very small.

Senator O'NEILL: And you did make payouts under income protection during the course of that year?

Mr Grobler: We do not make any payouts; the insurer would have made them.

Senator O'NEILL: You are going to provide me with some more details about the mental health descriptors that you use.

Mr Grobler: It is important at this point, for clarity's sake: we fully underwrite for mental health on our life insurance products; mental health is excluded on income protection, and that is consistent with most of the products in Australia through the direct channel.

Senator O'NEILL: I understand that that is an effective business model. I put on the record here, as I have on a number of other occasions: the problem with that for small business owners is that they are now aware that this is a problem. If they get refused by one, they have to disclose that to everybody else, if they seek insurance somewhere else. They are also not seeking treatment for their mental health condition, because they do not want to jeopardise their insurance. Are you aware of this problem for Australian businesses?

Mr Grobler: I am aware of it but, in our instance, we underwrite the income protection product and we do not underwrite the mental health condition. So we do not decline them; it is a policy-wide exclusion. Before we take disclosure, we will make the applicant aware that mental health is not covered under this policy. For argument's sake, let's say somebody had schizophrenia: they do not have to disclose that to us, because we do not ask it of them. We mention upfront that there is a mental health exclusion and, at that point, they might elect to not continue the process, but we do not decline them. They can still qualify for the policy.

Senator O'NEILL: I understand. Let me dig a little deeper. Say I was engaged in a pretty messy divorce 15 years ago and had a period of being particularly unwell. I was perhaps not fully diagnosed as suffering from full depression, but I still undertook some chemical treatment to assist me through that crisis, and I did not disclose that to you. What would happen to my claim?

Mr Grobler: On income protection?

Senator O'NEILL: On income protection.

Mr Grobler: If there is a claim due to mental health, it would be excluded.

Senator O'NEILL: So it does not matter about the scale before—

Mr Enthoven: On life insurance, in that particular situation you would likely have been approved and then be covered.

Mr Grobler: Yes.

Senator O'NEILL: Would I have been forced to disclose that because of your questioning on life insurance?

Mr Grobler: On life insurance, yes.

Senator O'NEILL: Would you be aware of incidents such as that?

Mr Enthoven: If they are disclosed to us, yes, we would be.

Mr Grobler: Only if they claim—

Senator O'NEILL: Do you ask questions to get that disclosure? One of the concerns we have is that people do not actually claim that they were depressed, even though your definition of depression is if you have had a period on Zoloft. They were just looking after themselves—it was just the break-up. That is what a lot of people describe. They do not pathologise it or call it depression, and often doctors do not do that because it can be paralysing for people to be labelled when they are just dealing with their grief and, 'This will help you with your grief,' is the conversation that the doctor might have.

Mr Enthoven: We are well aware of the complex nature of this issue, we have great sympathy for the consequences of the industry's current practices and we are working with our insurers to try to be more proactive in solving this problem. But, as I am sure you have heard from other participants in the industry, it is a highly complicated and multifaceted challenge, and, to some extent, it will require a significant leap of faith by the industry to deal with this.

Senator O'NEILL: What might that look like?

Mr Enthoven: I am not an expert in this, but my own personal view is that the industry will find a solution for mental health in the next decade.

Senator O'NEILL: Will that involve definitions standardised across the industry?

Mr Enthoven: We are a small business that distributes a limited number of policies. There are people in the Financial Services Council, board members, who are working on this. I am very keen for us to find a solution as an industry to this specific issue, and I do believe that we will because I think the community's expectations are that we have to find a solution. My view is that the consumer ultimately wins, and consumers are engaged in this issue now, and so the industry is going to be forced, in my view, to find a solution. But it would be premature of me to hold myself as some expert or to give you a view as to where that might land.

Senator O'NEILL: I know that you used 10 years just as a throwaway figure, but I would hope that there might be something happening a lot more quickly than that in terms of the industry undertaking the responsibility for this area, which is a burgeoning challenge to the industry, but also for clarity for the community and for access to people who do have an episode of feeling particularly unwell mentally and then get over it, live their normal lives and then perhaps encounter that further down the track.

Mr Enthoven: And those customers for our life insurance policies, which are the vast majority of our business, would in fact be able to acquire cover and there would be no exclusions for that condition.

Senator O'NEILL: I think Senator Williams raised independent medical examiners. You said that that is really with your insurer. I have some questions on that. We might get you to send them on to your insurer and give us some detailed responses. That would be good.

What are the laws and regulations that apply to you with the use of investigation and surveillance of claimants and what are your practices?

Mr Enthoven: We do not ever appoint investigators. Our life insurance partners may. I am not aware of that happening.

Senator O'NEILL: Could you take that on notice and provide some detail?

Mr Enthoven: I am happy to take that on notice, yes.

Senator O'NEILL: If they do, could you advise if they use their own staff or engage third parties to conduct those investigations. And, if you have the chance, you might think about the professional standards and accreditations required to undertake such practices. My last line of questioning is around genetic information—again, you might need to refer this on. Are you aware of the use of genetic information as part of the business model that you are the front for?

Mr Enthoven: We do not use genetic information in our business model. I was in the audience during your previous session, and I heard you ask if I am aware of it being used. I am not aware of it being used.

Senator O'NEILL: In Australia?

Mr Enthoven: In Australia. I am aware of it being used in other jurisdictions.

Senator O'NEILL: Which ones are they, to your knowledge? If you want to take it on notice, you can.

Mr Enthoven: I will take it on notice. My understanding is that it is being used in the United States.

CHAIR: Is it being used to exclude people or is it being used to reduce premiums?

Mr Enthoven: It is being used to assess risk. To be fair, that is my view; I cannot speak factually on that.

Senator KETTER: ASIC report 498 dealt with the issue of life insurance claims. There was a review done, and there was a finding that the direct or the non-advised sector had higher decline rates. You have touched on this issue with Senator O'Neill. In terms of the non-advised sector, how does your decline rate compare with the average in that sector?

Mr Enthoven: I think, at 2.1 per cent across our product suite, we vastly outperform any of the data that we saw in the ASIC report. Broadly, we are comfortable that we are—and, to be clear, our data was not solicited. It was solicited via our insurers but, nevertheless, we compiled the data and independently and involuntarily provided it to ASIC, because we think that the presentation of their data was methodologically unsound. In my view, duration is a very important issue, but another very important issue is policy exclusions. There are group policies; there are advised polices, and there are direct policies which have exclusions. Ultimately, it is those exclusions which cause claims denials over time. Therefore, my own view is that it is less channel sensitive and more product construct sensitive in terms of the denial rates.

Senator KETTER: The advised sector would say that it is because people are not aware of exclusions lying within the PDS that they are not told about.

Mr Enthoven: I think there are cases in the advised sector where that happens. There are cases in the direct sector where people are not aware of exclusions that they may be acquiring in their policy. As we have said, we could charge a lot less for our product if we had exclusions, but our view is that it is ultimately not a sustainable business model.

Senator KETTER: ASIC formed the view that there were potential issues and sales practices for direct or non-advised policies. Do you think that is a flawed finding?

Mr Enthoven: I think ASIC is about to conduct a review of the direct life industry, and we will actively participate in that and work with ASIC to help secure best practice outcomes. I am concerned that, within the direct and other distribution channels, there are policies with broad pre-existing condition exclusions which, like I said earlier, I believe will create reputation and brand risk for the industry going forward.

Senator KETTER: Your staff operate under your financial services licence.

Mr Enthoven: That is correct.

Senator KETTER: Can you tell us about the training that your staff receive and what they are required to do to keep up their registrations.

Mr Grobler: Due to the fact that we do not provide any personal advice, our sales staff are not qualified for RG 146 et cetera. They undergo internal induction training and ongoing product and upskilling training. This is all facilitated through our internal L&D department—our learning and development department. But they are not equipped or trained to provide any level of advice. They provide factual product information to the customers and navigate through it carefully and systematically under our rules, where they read the questions verbatim and all their calls are recorded. They do not vary off that path.

Senator KETTER: And their remuneration structure is not tied to any sales outcomes?

Mr Grobler: The remuneration is predominantly a fixed base. There is a small component available, and that is based on various internal metrics that we measure. There are a range of items we measure their performance on.

Senator KETTER: But are they linked to sales outcomes?

Mr Grobler: One of the outcomes is premiums sold in a month. But they are also penalised if that premium ends up cancelling later—and we do this to specifically prevent any aggressive selling tactics. We will take that commission off them. As Richard mentioned earlier, our quality assurance department will review the sales calls, and if they did not adhere to our compliance regime then they will earn no reward on that activity.

Mr Enthoven: And would be subject to disciplinary action.

Mr Grobler: And it might lead to termination.

Mr Enthoven: To give you some context, over 80 per cent of our wage bill is fixed. There is a small component which is variable. That does include premiums sold as a factor, but it does also include a QA score, compliance et cetera.

Senator KETTER: I noted that you had been awarded a particular award in three of the last five years, that of preferred life insurer. But in every firm there is always potential for issues. Given that your function is promotion and marketing of the Real Insurance branded policies, how can we be confident that there are not going to be any sales practices which fall in line with what ASIC has found or suspected?

Mr Grobler: I think, firstly, it is important to know Greenstone will not outsource any of those functions that Richard listed earlier. We do not outsource that to other providers. All we have is our customers, so we will try and do the best by our customers over the life of these policies and as long as they remain customers with us. Our internal quality assessors will ensure that those sales staff adhere to all the required steps they need to fulfil.

Mr Enthoven: For each team of 10 salespeople there is a team leader who is monitoring those 10 people all the time. Over and above that there is an independent quality assurance process, and over and above that there is an internal audit process. We also get audited regularly by our underwriters to ensure that we are complying with their rules. So there is a very robust quality control program in place. That is not to say we have not had individual cases. Where we have individual cases we treat them very seriously, and it is grounds for dismissal.

Senator KETTER: ASIC is urging all companies to review their remuneration arrangements to ensure they support quality outcomes for consumers and better manage conflicts of interest. Is that something your firm is doing?

Mr Enthoven: We have already done that in our view. As I said, over 80 per cent of our wage bill is fixed, and there are no incentives for sales only. So there is nobody who can earn any extra income just for making sales. It is a balanced scorecard which includes sticking to scripts, quality assurance et cetera.

Senator KETTER: How does that at-risk component of the remuneration compare with other direct channel life insurance companies that you are aware of?

Mr Enthoven: It is very difficult for us to comment on other people's compensation schemes.

Senator O'NEILL: Can I ask about the balance on that scorecard. Is there a percentage weight to all of those different elements you have identified, or is it unweighted?

Mr Enthoven: There is an overriding variable which is that if you breach your compliance obligations there is zero commission. That is a hurdle, effectively, that needs to be overcome to get anything.

Senator O'NEILL: Right. Do all of your staff meet that basic hurdle?

Mr Enthoven: No. From time to time we have had staff that breach. They do not get commission, and disciplinary proceedings are commenced.

Senator O'NEILL: What percentage of your staff would that be?

Mr Grobler: A small percentage. It could be that they are placed under performance management, where they go back into the coaching or the training and development arena, and only once they have completed that to our satisfaction will they come back onto the sales floor. But it is a small percentage.

Senator O'NEILL: Assuming they pass the hurdle, what percentage allocation to the other elements that you were discussing with Senator Ketter do you place on sales as opposed to other quality assurance measures? How do you calculate that on the balance scorecard?

Mr Grobler: It differs from sales unit to sales unit. They sell various insurance products, not only life insurance products, so there is quite a detailed and integrated remuneration structure.

Senator O'NEILL: Would you be able to provide that to us on notice?

Mr Grobler: We could certainly.

Senator O'NEILL: And could you give me a general understanding in the life insurance sector?

Mr Grobler: It is difficult to compare. As I mentioned, some distributors out there will outsource the sales function. Typically, that is done on a time-and-material basis where they will pay the sales staff or the third party a once-off fee. But I do not have information on how they are remunerated.

Mr Enthoven: Our understanding is that our fixed versus variable is more fixed than what other people are seeing in the industry. The reason we know that is that, when people join us from our competitors, they comment that one of the reasons they have joined us is that there is less at-risk compensation.

Senator O'NEILL: I think I have a misapprehension in my head that you have got a group of people who are together, you are closely supervising them and you have got these standards there. But I think I just heard you talking about almost subcontracting?

Mr Enthoven: No, no. We do not outsource anything, categorically.

Senator O'NEILL: Right. So what did you just say about—

Mr Enthoven: Our view is that the fixed component of our compensation is higher than that of our competitors and obviously it is much higher than—

Senator O'NEILL: Yes, but before that you were talking about not being sure what happens—

Mr Enthoven: I am not sure about what happens with other companies, not our customers. Our customers are 100 per cent serviced by our staff.

Senator O'NEILL: I will check the record to see what I heard and I might ask a question.

Mr Enthoven: I apologise if I was not clear or misspoke.

Senator O'NEILL: No, I think it was something Mr Grobler said. Thanks.

Senator KETTER: Just finally, has ASIC been in touch with you yet in relation to this review?

Mr Enthoven: Yes. Actually, we volunteered. We volunteered to meet with them, present our claims data and give them an update on the industry. We understand that their review of the direct life industry is going to commence imminently.

Senator KETTER: I think it was supposed to commence before January.

Mr Grobler: It has commenced already and, via our life insurer, they have already requested certain information. They raised the request for information with Hannover Life Re and, subsequently, because we perform most of those functions, we are working with Hannover to supply that information. I think that is due back to ASIC at some date in June.

Mr Enthoven: Sorry—when I say 'commence', I mean commence engaging on a face-to-face basis with the industry. But we have a very close and active relationship with ASIC. We regularly meet with them and update them. Our modus operandi is that we are much better off with them knowing and understanding our business than hearing things through the grapevine.

Senator KETTER: Mr Grobler, I might have misheard what you said. Did you say that ASIC is likely to complete its investigations by June?

Mr Grobler: No, I do not believe so. That is when the first round of information is due back to them. We will supply the information to Hannover Re and they will then supply it to ASIC sometime in June.

Senator WILLIAMS: [inaudible] will be working for ASIC next week, Senator.

CHAIR: We are nearly out of time and I believe the deputy chair has two quick questions.

Senator O'NEILL: I do. One is about the multiple policies and single payout. Are you impacted by that practice in any way?

Mr Grobler: No, we do not ask questions on existing cover. We will not be able to manage that information as our sales agents do not provide any advice. We can offer life insurance policies between the sum of $100,000 and $1 million and it is up to the customer to decide what cover they require—

Senator O'NEILL: And, if they have cover with you, and they have cover with MLC and they have cover with somebody else, you would still pay when they claim?

Mr Grobler: Yes.

Senator O'NEILL: Because we have been hearing about multiple policies and single payout.

Mr Grobler: On income protection it is different. There is no impact if you have life insurance through your superannuation fund and maybe another two or three stand-alone life insurance policies with other providers. That will not impact on a real life insurance policy. On income protection though, there is a standard clause in most income protection policies that says they can offset other income. Once you are disabled and you can no longer generate an income but you are getting income from two other income protection policies already, it would be unfair on top of that for you to end up earning more than you earned before you were disabled.

Senator O'NEILL: I understand that it is to deincentivise that sort of corrupting.

Mr Grobler: But in those instances, the premium would be returned pro rata. For instance, if somebody was insured for $5,000 per month benefit and we end up after offsetting only paying them 2½ thousand dollars, half of their premium would be refunded because in that instance they were over insured.

Senator O'NEILL: If there was another insurer involved, who decides which policy is paid out on? How do you determine that?

Mr Grobler: I think it is first in to first base—

Senator O'NEILL: So you make your claim—

Mr Grobler: We do not assess that. That would be the insurer. They will assess what income is accessible from other policies and they can offset.

CHAIR: How would they know?

Senator O'NEILL: That is the question.

Mr Enthoven: We will take that on notice. We do not do that. I am not suggesting that it has not happened but, personally, I do not recall a single case of that happening to any of our customers.

Senator O'NEILL: The last one you mentioned was codifying financial hardship. There have been a few submissions that talk about it. What do you mean by that, and what do you recommend?

Mr Grobler: From 1 July, all of the insurers that we work with have signed on to the code of practice. They have agreed what the internal financial hardship processes will be. We manage that on their behalf because in all instances it is responsible for premium collection et cetera. From memory it is if a customer suffers from financial hardship—and there is a definition around that—we will waive up to three months' worth of premiums while they still stay on cover. That is to help them through periods where they might have been retrenched or they are without income while they look for new employment. We will waive the premiums for that period and then they continue to pay thereafter. But they stay on cover.

Senator O'NEILL: Is that three months standard practice across the industry?

Mr Grobler: It is open to the discretion of the insurers but that is what our insurers agreed on.

CHAIR: Thank you, gentleman. We are out of time and the other witnesses are here. We appreciate you coming here today. Answers to any questions taken on notice should be provided to the secretariat by 9 June 2017. Once again, thank you for attending the hearing and for your evidence today.

ANDERSON, Mr Philip, Chief Operating Officer, Life and Investments, Zurich Financial Services Australia Ltd.

BAILEY, Mr Tim, Chief Executive Officer, Life and Investments, Zurich Financial Services Australia Ltd.

[11:33]

CHAIR: Gentlemen, welcome. I remind committee members and witnesses that while this is a public hearing, care should be taken to protect the privacy of individuals and that arguments should be made without naming individuals. I invite you to make a short opening statement. At the conclusion of your remarks, I will invite members of the committee to put questions. If your opening statement is in a written format, one of the secretariat might ask you for a copy after you have finished. Thank you.

Mr Bailey: Thank you for the opportunity to be here today. We have operated in Australia under the Zurich brand since 1961, making us one of the few life insurance brands still active from that time, so we have both longevity and stability. Last year we acquired Macquarie Life's risk business, further reinforcing our commitment to the Australian market. As a member of the FSC and a major life insurer, we have naturally observed and participated in the ongoing reform dialogue.

We are supportive of more transparency and accountability in the sector, and we believe the FSC Code of Practice and enhanced reporting of claims and lapse data are steps in the right direction. Whilst recognising there is more to be done we are optimistic that these changes, combined with the Life Insurance Framework and professional standards, will help rebuild consumer trust in our sector and help to deliver a more informed and more protected community.

As part of a Swiss company we have a reputation for being prudent and running our business in a sustainable way. It means we are financially healthy, with a good quality customer base, which maximises our ability to deliver on our core customer promise of paying claims. We believe that consumers should have the choice in how they buy life insurance. We also believe they should have the means to make informed decisions. This is why we believe a vibrant, growing financial advice profession is vital, and this is why Zurich is a longstanding champion of the financial advice profession. The majority of our business is written through financial advisers.

Making financial solutions more accessible means making them more available, affordable and understandable, and to this end we believe there is too much complexity in our sector, and, as an industry, we should strive for greater simplicity. The duty of care to customers and communities is one that all major life insurers take seriously, and it is certainly at the very centre of everything that Zurich does.

The opportunity to improve exists in every facet of life, and our industry is no different, but it would be a great shame if the ongoing coverage around the sector drives people away from protecting their livelihoods. We hope that by sharing our insights we can contribute to the process of returning the reputation of life insurance to its rightful place as a vital, respected industry known for putting customers first.

CHAIR: Thank you. In regard to your comment about returning it back to the reputation it had previously, where do you think the reputation is at present, where do you hope it will get back to and what steps do you think you need to take to get back to that point?

Mr Bailey: We clearly need to improve confidence in the sector. There have been challenges over the last couple of years. I do believe that the steps that we are taking as an industry—implementation of the Life Insurance Framework, professional standards and advice, code of practice, looking at areas like minimum standard definitions, enhanced claims reporting, enhanced lapse reporting—are all steps forward in the right direction to provide more transparency and start to provide that confidence. Essentially, we need to operate in a sustainable way which is consistent and in our customers' best interest. That is certainly what we are aiming to do.

CHAIR: Were these steps initiated, as Senator Williams asked one of our witnesses this morning, after the TV show that showed CommInsure a bad light, or were you already on that path?

Mr Bailey: I believe we were already on that path. The conversations around the Life Insurance Framework started in 2014, or sometime before that, and I think we were having a constructive dialogue at an industry level before then around making further enhancements. I do believe we have accelerated progress over recent times, which is essentially a good thing.

CHAIR: Do you have insurance policies that are not underwritten at the time of the collection of premiums?

Mr Bailey: No, 96 per cent or so of our business is written through financial advisers, which all goes through a very full underwriting process. We have a small direct portfolio which also goes through an underwriting process. With the direct business it is a more simplified underwriting process; it varies by product, but all of our products are underwritten at the time of inception.

Senator WILLIAMS: Zurich has pulled out of group insurance, I noticed. You are no longer involved in group insurance?

Mr Bailey: Correct. We are not in the group market. We pulled out of the group insurance market in 2014, so we have a very small runoff portfolio now. We essentially exited group in 2014.

Senator WILLIAMS: Most of your business is done through retail, which I think is a very good thing. How many financial planners does Zurich have? Roughly—I do not mean the exact number; I am not going to hang you up to dry if you say, 'Look, we've got 326,' and there are actually 327.

CHAIR: Are you sure?

Senator WILLIAMS: Positive.

Mr Bailey: That is good because I cannot give an exact number. We would typically write business in any given year through around 3,000 financial advisers. We do not own any advice directly. We are a pure manufacturer.

Senator WILLIAMS: You do not have your own advisers?

Mr Bailey: We do not have our own advisers, no. We operate purely through independent financial advisers, and there would be around 3,000 to 4,000 financial advisers who write Zurich products.

Senator WILLIAMS: Do you have ties with the banks and their advisers as well? I am referring to contracts and agreements.

Mr Bailey: We have positions on approved product lists across many parts of the industry, so that would be across the institutionally-owned and bank-owned licensees through to medium-sized and smaller sized licensees. We have positions on approved product lists across a number of parts of the advice industry.

Senator WILLIAMS: You would have seen the *Four Corners* story about the outdated definition of a heart attack, which was two milligrams per litre of troponin, or whatever the figure was. Under the new measurements of troponin you could never get above 0.5, so you could never reach the criterion. Have you reassessed your criteria standards when it comes to those serious problems and trauma events such as heart attack?

Mr Bailey: I will kick off, and then, Phil, feel free to add. Our process is we would update our products typically twice a year, so on a six-monthly basis. When we go through those product updates we will look at definitions at those points in time to understand where we need to update definitions to match changes in medical advancements. In the case of the heart attack definition—in terms of a specific instance last year—we had already moved to an updated definition, I believe, in 2012, so we had changed that definition.

Senator WILLIAMS: Tell us the changes you made then, Mr Bailey? What were your changes to the definition of heart attack when you changed it in 2012? What was it in 2011, and what is it now?

CHAIR: Take it on notice.

Mr Bailey: I would need to take that on notice.

Senator WILLIAMS: Please do.

Mr Anderson: I might just add, Tim—

Senator WILLIAMS: Please do, Mr Anderson.

Mr Anderson: In our current heart attack definition, definitely troponin is not one of the criteria. Just looking at the wording of our definition, which I took a copy of, we list what the criteria are—so, cardiac changes, ECG tests, et cetera—but also, and importantly, we actually do have a clause that says:

> If the above tests are inconclusive or our noted diagnostic techniques are impractical to apply or have been superseded, we will consider other appropriate and medically recognised tests.

That is in our policy.

Senator WILLIAMS: One of the complaints people bring to me is this clarification of TPD—totally and permanently disabled. I was told years ago that if I was a right-handed orthopaedic surgeon and I used my right hand to hold a scalpel and the tools to do knee replacements, hip replacements, or whatever, and I had my right arm chopped off that I would be classed as TPD because I could not carry on in my profession. Is it the case now that being classed as TPD under your criteria means you basically cannot shave yourself, toilet yourself or walk around? Are they your criteria now for TPD?

Mr Bailey: I will start now, and Phil can continue.

Senator WILLIAMS: Explain your criteria.

Mr Bailey: I think one key aspect to consider is 'own occupation' versus 'any occupation'. As consistent with the rest of the industry, we would offer the choice of those two types of cover. Clearly, it is a narrower definition as it relates to 'any occupation' rather than 'own occupation'. It does depend on whether the coverage that you have relates to an ability to undertake your own occupation or any occupation. I think that is an important, relevant point in this discussion.

Senator WILLIAMS: You basically have a separate policy?

Mr Bailey: It is a separate option.

Senator WILLIAMS: If I am a specialist surgeon and my hands are my livelihood, if I insure for TPD I would pay a higher premium because I would be covered under that specific occupation—is that what you are saying?

Mr Anderson: Yes, if you have an 'own' definition TPD then it is your occupation. So if you cannot perform as a surgeon, that is your occupation.

Senator WILLIAMS: Did you want to interrupt, Chair?

CHAIR: I am just clarifying. Obviously there is a difference in the premiums.

Mr Bailey: Yes. There is a difference in the premiums on that basis. Clearly the level of risk from the insurance perspective and the likelihood of claim are different across those two coverages, and the premium is different on that basis.

CHAIR: I was not going to ask you what the premium for a senator would be!

Senator WILLIAMS: Yes, it has to be different because we work a bit harder than you lot down there, who work bank hours!

Senator O'NEILL: That is a general comment!

Senator WILLIAMS: What percentage of claims has Zurich rejected?

Mr Bailey: It varies by type of cover. But in the case of death claims, if you take the periods that we reported to ASIC last year, we paid 99 per cent of claims, and we would have rejected one per cent of death claims.

Senator WILLIAMS: Is that both retail and direct, Mr Bailey?

Mr Bailey: That is across retail and direct. I would need to check the exact statistics for direct, but it is such a small part of our portfolio that it does not impact the overall percentage, if you like. Clearly that is for death claims. The rate of decline would be higher on other coverages, but we were below the industry average for declines on all four of the coverages—across term, TPD, trauma and income protection—in the statistics reported last year.

Senator WILLIAMS: What about disputes? How do you handle your disputes resolution? Are they in-house? When someone says, 'Look, I've been treated badly by Zurich; I've had a claim; you've rejected it; I don't think you've treated me fairly,' how do you handle those disputes? You seem to be keen to answer, Mr Anderson.

Mr Anderson: We have an internal process which is a claims review panel. So, if a customer does complain, and in particular about a complaint, we will go to the claims review panel. That is chaired by an independent person. We have another independent claim and legal expert who sits on it as well as some of our internal people, me included. We look at it. We simply look at it as: is the decision the right decision; is the decision a fair and reasonable decision from the customer's perspective? If we do not think it is, then we can overturn the decision. If we think it is an appropriate decision, then we provide that guidance as well.

Senator WILLIAMS: If I am the customer and I say, 'Well, you might have reviewed it in your in-house independent panel, but I'm not happy,' where do I go to then?

Mr Anderson: To the financial ombudsman.

Senator WILLIAMS: Yes. That would be the next step. I have not seen the full detail of the tribunal the government wished to establish, or the extended FOS, but it is basically a one-stop shop for loans, investments, financial advice, life insurance et cetera. Do either of you have an opinion on the formation of that tribunal?

Mr Bailey: I think from my perspective we would just support anything which makes the process as transparent as possible and as easy to access as possible for consumers.

Senator WILLIAMS: And hopefully quick and hopefully inexpensive, not like a court system.

CHAIR: Before I pass on to the deputy chair, I would just like to expand on the group insurance. Why did you get out of it?

Mr Bailey: It was before my time in this role. I joined in 2015. Essentially, we have a large and growing retail business, and I guess that is our specialism capability. We had a small group business, and I think that, to compete effectively and offer a strong proposition in the group insurance market, you really need to have scale. I think it was really a question of saying that we did not have that scale and that our core focus and core capability was in the retail space.

Senator WILLIAMS: It offered you a better deal, in other words.

Mr Bailey: As I say, really we had a very small group portfolio and—

Senator WILLIAMS: So the big player probably undercut you on the quote.

Mr Bailey: There is a scale problem, yes, exactly.

CHAIR: I did have another question, but I will pass over.

Senator KETTER: Mr Bailey, you mentioned approved product lists, and I think you said that your products are sold by around 3,000 advisers. Are those advisers within vertically integrated groups or in independent—

Mr Bailey: Both. It would really be right across the financial advice sector, so that would include advisers who are within licensees that are owned by institutions, and it would also include advisers through to medium-sized and small-sized independent licensees.

Senator KETTER: Are you familiar with the concept of shelf space fees?

Mr Bailey: I am familiar with the concept of sponsorship, which we would pay as part of the arrangements for those APLs. That would typically cover education, training et cetera on Zurich products for the advisers within those licensees.

Senator KETTER: Can you tell us how much—what sorts of fees you are paying in that regard?

Mr Bailey: Those fees are set by and agreed with the licensee, so it is probably not appropriate for me to comment on the specifics of those, but I am certainly happy to provide that through an appropriate process on notice.

Senator KETTER: I would appreciate that. Perhaps you could tell us: how do those fees compare, for example, with the vertically integrated groups on the one hand and the independent groups on the other? Is there a difference?

Mr Bailey: I do not think there is a difference per se whether it is vertically integrated or not. I think that it does tend to reflect the scale of the licensee, so the number of advisers and the amount of training and education which would be required to therefore cover all of those advisers. I would see that as being the primary driver rather than anything else.

Senator KETTER: It has been put to the committee that some of those shelf space fees that are charged are disproportionate to training costs, or education costs, of advisers. What is the process behind determining the fees? Is it purely negotiation, or is there some transparency in relation to the charge going back to the training or education?

Mr Bailey: We would agree with the licensee what is covered in terms of the education and training activity that would happen as part of that fee. So that would be professional development days, conferences, training materials et cetera, and we would have transparency of what that is.

Senator KETTER: Are you familiar with the FSC's work in preparing a draft APL standard?

Mr Bailey: I am, yes.

Senator KETTER: I understand that their standard—and this may have changed; I am just looking at an earlier submission to us—indicates that two product APLs are acceptable. Under that standard, there seems to be no sanction proposed for an APL with only one insurer. Are you having any input into this standard?

Mr Bailey: The debate around that standard at an FSC level is primarily being driven through the Advice Board Committee rather than the Life Board Committee, of which I would be a member. I think that we see what is there as a step forward around transparency of who is on the APL and the basic provision of requiring more than one provider. I think our view would be that probably three providers are something which would give more appropriate coverage and access and choice for advisers and consumers.

Senator KETTER: In terms of the APLs that you are currently placed on, can you tell us: what is the range of providers that are on each of those APLs?

Mr Bailey: Yes. It does vary, but the majority of them would be four to five providers on those APLs. That would be probably the most typical number.

Senator KETTER: In the vertically integrated groups, would I be correct in assuming it is a lesser number?

Mr Bailey: It tends to be between three and five. I think there are two bits with the vertically integrated groups. You have the bank-salaried planners, and the number of insurers on the bank-salaried planner APLs does vary. With the aligned dealer groups, which are owned by the banks, those would tend to be typically four to five providers on the APL, with processes to enable advisers to go off APL if there is a product off the APL which would be in the best interests of the customer.

Senator KETTER: Just going back to the education and training: can you tell us: typically, what is involved in the training and education of advisers in respect of your products?

Mr Bailey: Yes. That would be a combination of things like professional development days and training materials. We would have access to the advisers to be able to provide content detail on the Zurich product and proposition. It would include attendance at conferences and things like that to be able to, again, provide access to information around the Zurich products and the Zurich proposition. It tends to be that type of thing.

Senator KETTER: Would these be Zurich conferences or generic?

Mr Bailey: No, the licensee conferences. I am happy to provide more information to you on notice if that would be helpful.

Senator KETTER: Yes. Thank you.

Senator O'NEILL: I will just follow up on that line of questioning. That was where I wanted to go as well. Could you explain what such a conference might look like, where it would occur and who would be in attendance.

Mr Bailey: It will vary, but typically it will be the financial advisers. You will have some of the manufacturers represented as well. There will often be experts in particular fields.

Senator O'NEILL: Just go right back. How many people? How many from Zurich? How many from others? Where does it occur, and how frequently?

Mr Bailey: They vary greatly.

Senator O'NEILL: Okay. Give me a big one and a small one.

Mr Bailey: Okay, a small one.

Senator O'NEILL: You have been to these things. I do not have any sight of them, so I am trying to get a visual and a richer understanding.

Mr Bailey: That is fine. I have to say personally—and probably for Phil as well—that I do not attend many of those conferences. It tends to be other members of our team, so it is a little bit difficult to comment fully. The conferences could be in a variety of locations. It could be a one-day conference which is basically content from expert speakers and potentially from the product manufacturers. It may be more appropriate for me to take that question on notice.

Senator O'NEILL: In Sydney, do product manufacturers all chip in to create these conference days, or is it Zurich-specific?

Mr Bailey: These are not Zurich-specific. We would do professional development days where we would invite advisers to attend events where we are providing information about Zurich, or they could be licensee conferences, which are really driven by the licensees and which are more about attendance of their advisers.

Senator O'NEILL: Which banks do you have your products in?

Mr Bailey: In terms of where we are represented on APLs, as I mentioned, there are aligned dealer groups which are owned by the banks, and we have positions on a number of those APLs. I cannot give you an exact number. Again, I am happy to provide further detail on notice as appropriate.

Senator O'NEILL: But who are you with? Which banks are you with?

Mr Bailey: In terms of the salaried financial planners, we are a member of the APLs of ANZ and NAB.

Senator O'NEILL: And only those two?

Mr Bailey: In terms of the salaried planners, yes.

Senator O'NEILL: And in terms of the other group?

Mr Bailey: The aligned dealer groups?

Senator O'NEILL: Yes.

Mr Bailey: We would have APL positions on some of the aligned dealer groups under the ownership of each of the banks.

Senator O'NEILL: So ANZ?

Mr Bailey: Yes.

Senator O'NEILL: And NAB?

Mr Bailey: Yes.

Senator O'NEILL: And Commonwealth?

Mr Bailey: Yes.

Senator O'NEILL: And Westpac?

Mr Bailey: And Westpac.

Senator O'NEILL: Anyone else?

Mr Bailey: AMP, and then a suite of APLs across the rest of the market. That basically means that those advisers have access to Zurich products. In terms of inclusion on those APLs, the licensees run a very robust due diligence process, which would look at our service record, our claims record, our products, the overall quality of our proposition, competitiveness, underwriting approach et cetera.

Senator O'NEILL: And they think you are pretty good to put you on, and then they say, 'We think you're really good, but you'll have to pay us some money as well,' and they then use the money that you pay to them to do what?

Mr Bailey: Predominantly education and training of their advisers.

Senator O'NEILL: Which is provided by you, or provided by somebody else. Do you pay them to let you train their people?

Mr Bailey: It supports the cost of training on the Zurich proposition.

Senator O'NEILL: So you pay them, but you do the training as well. You pay them twice: you pay them money and you also pay them with your expertise.

Mr Bailey: We need to contribute some of the expertise, clearly, yes.

Senator O'NEILL: But you pay them money as well?

Mr Bailey: To support their costs associated with that education and training.

Senator O'NEILL: Which you provide.

Mr Bailey: They have costs associated with that education and training as well. As I mentioned before, I am happy to provide more information on notice, if you want the detail.

Senator O'NEILL: That is great, but I am trying to understand how the whole thing works, because basically this is shelf space that has turned into, 'Now we pay an education fee.' We have heard that the quantum of money is quite significant; this could potentially be a corrupting behaviour that is not necessarily good for the industry or for clients who are seeking insurance; the limitation of options that have been provided by the banks through their financial advisers, whether it is salaried or otherwise, is not a full enough suite to give Australians the best value for their money; and it is not transparent. That is where I am coming from.

Mr Bailey: I think the draft under the APL standard ensures that that transparency needs to be there. As I mentioned, our view is that there need to be probably more than two providers to provide appropriate choice across APLs. I think the balance, if approved product lists get wider, is that then really requires advisers to have the full, detailed knowledge across all of those products. I think there is a trade-off between the breadth of choice and their ability to fully understand the breadth of propositions, especially given that we are in a very competitive market where all of us are continuously looking to improve our customer proposition.

CHAIR: With this APL, is there a point or a level where you say, 'It's not worth being on their APL, because they're not writing enough policies for us, so we're just not going to pay the fees anymore'? Do you have that in your structure?

Mr Bailey: We would typically look at the partnership. It is really about the time and effort and resource that we put into that partnership and the cost of doing that. Clearly, yes, we would look at whether that is consistent with the level of business we have with that partner.

CHAIR: Not at that point, I would say.

Mr Bailey: Yes. But I would stress they are typically ongoing partnerships. The key aspect, given the quality of the research process that we see, is very, very robust in terms of assessing product capability, underwriting claims, service levels, longevity, stability et cetera. It is a very robust process. We see that as a very positive thing

which enables advisers to have access to providers and products which have been through that very, very robust due diligence process by the licensees.

Senator O'NEILL: I am just ruminating: new players would have to have an awful lot of money. How much do you have to have to get into this?

Mr Bailey: I am happy, as I said, to provide that. What I would say is that I think they are much less significant than perhaps you may believe, but, again, it is a commercial arrangement. I would be happy to provide it in private, on notice.

Senator O'NEILL: Where are the conferences that people go to located?

Mr Bailey: Again, it can vary.

Senator O'NEILL: Are any of them outside Australia?

Mr Bailey: Conferences can take place outside Australia, yes.

Senator O'NEILL: So that would be thousands of dollars to get people to attend those conferences?

Mr Bailey: Yes, based on the cost of airfares and accommodation et cetera.

Senator O'NEILL: How are people selected to go to those conferences? Is it a reward for effort or is it based on sales volume?

Mr Bailey: I think it is probably a question for you to be asking the licensees, because I think that is what you are asking about. As a product manufacturer, as Zurich, we do not run any conferences overseas.

Senator O'NEILL: If you were willing to take on the training yourself, would that be acceptable to the banks? Would banks still let you put your product in their suite if the only thing you offered them was to do the training at a cost only to you? Would that be enough, or do you have to give them money to be able to play in the game?

Mr Bailey: I cannot really answer the question on behalf of the licensees. As I mentioned, we do not own any licensees. But clearly there is an effort on both parts to ensure that that training is robust and appropriate, and I would imagine the licensees would want to ensure that they have a degree of control over that training process to ensure that they are very comfortable that their advisers have the right level of training.

Senator KETTER: What are the licensees' costs that you are asked to cover? You are providing the training itself, but you mentioned—

Mr Bailey: The licensees have to undertake a significant amount of training and education. Clearly they need our support in terms of the content of our proposition to help them do that. They are undertaking the majority of the training and education activities.

Senator O'NEILL: Why is that a cost to your business and not a cost to their business? Why are you paying for the training of their people in addition to providing training specifically about your product?

Mr Bailey: As mentioned, it is a contribution to that process to ensure that their advisers are appropriately trained on the relevant product set.

Senator O'NEILL: Is it just established practice and this is what you have to do to get in? Everybody says: 'That's just the way it is. This is the culture. This is what we've always done and this is what has to be done. If we're going to get on their APL, we've got to hand over the money.'

Mr Bailey: It is a reasonably well established process within the industry.

Senator O'NEILL: Yes, I guess that is one of the things with culture: it is often unquestioned. But, as an outsider, it seems extremely interesting that you pay twice—in kind with your knowledge and training about your product and also for their training more broadly.

Mr Bailey: I do not really see that we are paying twice. I would note as well—and I am sure you are aware of this—that those fees are flat fees. There is no volume component on those fees.

Senator O'NEILL: I will be very interested to receive more detail and read more about that.

Prof. Otlowski: We are happy to provide that.

Senator O'NEILL: Could you give me a bit of a precis of your approach to mental health and the underwriting of mental health, in both direct and advised life insurance. And do you do income protection too?

Mr Bailey: We do income protection. I will ask Phil to provide more commentary around this. As mentioned at the start, over 95 per cent of our business is written through financial advisers, and there is a very extensive underwriting process associated with that. In our smaller direct portfolio, we have a simplified underwriting process. However, it is still a full underwriting process.

Senator O'NEILL: Were you here for the Real evidence, Mr Bailey?

Mr Bailey: Yes.

Senator O'NEILL: Is it a similar process, or is it quite different? I know sometimes there is considerable variation.

Mr Bailey: I will pass to Phil to comment on this.

Mr Anderson: I am not familiar with the Real products, other than that I see the advertisements on TV, like all of us.

Senator O'NEILL: Were you here for their evidence today, Mr Anderson?

Mr Anderson: Yes.

Senator O'NEILL: You are as familiar with theirs as I am now, so let us go from there.

Mr Anderson: We have, essentially, three direct products. As Tim said, it is a very small part of what we do. Two of those products are what I would call simplified underwriting, but they are still relatively robust. By that I mean we have a life cover and an income protection cover. For the life cover, we ask a base 24 questions, and it is done through an underwriting engine. For example, if I applied and said, 'I've got a history of asthma,' there would be some further questions that would be asked. For the income protection product that we have, we ask 36 base questions and, again, we have a cascading—

Senator O'NEILL: So there are red flags and they answer some more questions?

Mr Anderson: Exactly. The other product, which is a very small percentage of—

Senator O'NEILL: Can you clarify with regard to mental health in that suite first?

Mr Anderson: We would normally ask, as we would normally ask for a retail product, about the history of mental health—stress, anxiety or depression. Those products do not have a mental health exclusion. Even though they are simplified, there is still a relatively robust underwriting approach up-front, and clearly that is designed to do what we want to do, which is be able to pay valid claims promptly.

Senator O'NEILL: Do you have the same approach of having a standard fee, a loaded premium, and then denial as a part of that process?

Mr Bailey: We would look through that underwriting process and clearly reflect that there are differing levels, depending on the nature of any mental health condition, the time elapsed since that condition, the time elapsed since any treatment and the work history over the intervening period. We would take all of those types of things into account before then concluding: can we offer cover on standard terms, does there need to be a loading, do we need to offer with an exclusion, or—

Senator O'NEILL: Deny?

Mr Bailey: Or can we not offer cover, yes.

Senator O'NEILL: And if you could take on notice just to give the stats on that split, that would be helpful—and what conditions you have excluded on as well.

Mr Bailey: And what conditions we would exclude on? Certainly, yes. I think we can possibly give you an indication of the stats first.

Mr Anderson: We have got from May 2016 to May 2017.

Senator O'NEILL: Sure.

Mr Anderson: We had just over 8,000 applications.

Senator O'NEILL: For direct insurance?

Mr Anderson: This is direct and retail, so it would be proportionate to the business that we write.

Senator O'NEILL: So what percentage is direct? You said it was only small.

Mr Anderson: It is three or four per cent.

Mr Bailey: It is around four per cent of our portfolio, so you can assume, basically, retail predominantly.

Senator O'NEILL: Okay, from the numbers. Yes.

Mr Anderson: Of those 8,000-odd applications, 14 per cent disclosed a history of some sort of mental depression or anxiety or stress. Relative to the total application, the number that were excluded or declined is very low; it is less than two per cent. If we look at the 14 per cent that disclosed a health history for the disability products, which I think is probably what you are more interested in, for total permanent disability 82 per cent were offered terms, with 43 per cent offered standard rates; and for income protection 70 per cent were offered

terms, with 30 per cent offered it at standard rates. And I would just say that, in underwriting an application, there are obviously other factors or other disclosures that are not related that could impact the decision.

Senator O'NEILL: Such as?

Mr Anderson: Any health history. I guess I am saying that the number of applications where it is isolated to the only disclosure being mental health—I do not have those numbers. I have a breakdown of standard rates, and with accepted and with exclusion applied. In the exclusion applied, I think that the majority—but, as I say, I do not have the numbers.

Senator O'NEILL: Yes. If you could take that on notice, that might be best.

Mr Anderson: Sure.

Senator O'NEILL: I think you understand what I am trying to get a picture of.

Mr Anderson: Yes.

Senator O'NEILL: Were you here when I was asking questions of NAB this morning?

Mr Anderson: No.

Senator O'NEILL: Okay, so I will just see if you are aware of the disclosure by MLC that they have a box on their application form where people tick it, and it provides them with the opportunity to—well, it gives permission for them to seek one of their proposed clients' full medical history. Do you have such practices, and what is your view about that?

Mr Bailey: We would ask that question of the client, and we do so through a very clearly disclosed process. It is a separate page which clearly articulates what we are asking the client to agree to.

Senator O'NEILL: Do you have a copy of that?

Mr Bailey: I am not sure we have a hard copy, but we can certainly provide it very easily, yes. It is a separate page, so it is clearly articulated, not in the middle of a series of text et cetera. It is very clearly separately displayed.

Senator O'NEILL: Give me a precis of what it says.

Mr Bailey: I would need to check the exact wording. The essence of what we are trying to do is obviously, through the underwriting process, to ensure that we have as full an underwriting process as possible, to understand exactly what the position is when we offer cover. Through doing that, we are ensuring that we are minimising the possibility of any disputes at claim time.

Senator O'NEILL: But you are maximising the potential invasion of privacy by undertaking that process for individuals who might not have seen the documents that you ask them to tick a box to reveal to you.

Mr Bailey: To tick and sign. It is very clearly disclosed. And it is really, as I say, that around the underwriting process we want to make sure that we have got access to all relevant information and avoid any possibility of disputes at claim time.

Senator O'NEILL: You do not see anything ethically wrong with that practice? Do you see anything wrong with that practice?

Mr Bailey: We make the request in a very clearly disclosed way—

Senator O'NEILL: Yes, you have made that clear. But is there any ethical consideration to what you are actually doing by asking people to tick a box to give you, as an insurer, access to their entire health record, which is now an e-health record and can be sent with the push of one key?

Mr Bailey: I think what we are trying to do is make sure we have clear information at the underwriting process and at claims time.

Senator O'NEILL: You already have an incredible degree of power. That just gives you every access to every detail of every individual.

Mr Bailey: Clearly, at claims time we request very specific information which would relate to a specific consideration—

Senator O'NEILL: Yes, at claims time, but up-front you ask for people's entire history.

Mr Bailey: No. We go through the underwriting process where we ask a number of questions—

Mr Anderson: With regard to requesting personal medical attendants' reports, we would be very specific. If somebody disclosed that they have a history of diabetes, we would write to the doctor and say, 'Can you please tell us about their history of diabetes, including their weight and their control through the different blood tests that

they use?' We would ask specifically. We do not go to a doctor and say, 'Can we just have all of Phil Anderson's health history?' We just—

Senator O'NEILL: That is not what you ask people to sign. You ask them, 'Can we contact your doctor about specific things you have disclosed in this process?' It is not a general absolution to get everything?

Mr Anderson: No. We do not know what people are going to disclose in any given case, so there is a medical authority that is on a separate page. It is not folded in small font in a signature section. It is a medical authority that says, 'I hereby disclose and give permission to Zurich'—or whoever the insurer is—'to access my medical records effectively to assess my application for insurance.'

Senator O'NEILL: 'To access my medical records' is pretty general.

Mr Anderson: Yes. I think the reason is that everybody's situation is unique and everybody's medical history is different. I think the previous witness, from Real Insurance, said—

Senator O'NEILL: They are also, generally, pretty private things, and there might be disincentives. This is what we are hearing. There are now disincentives for people to seek help from doctors because something will go on a record—for example, they might have had an episode of mental ill health—so they are avoiding getting the health care that they need for fear of not being able to get insurance. They know that the power has shifted so much against them, particularly small businesses seeking income protection.

Mr Bailey: It is important for the equity to all of our policyholders that we take account of the relevant information at the underwriting process to appropriately price risk and to minimise disputes at claims time. I would emphasise that is important to the equity across our entire portfolio. That is what we are essentially trying to do—make sure we have clearer information at underwriting. We are looking to offer the appropriate cover and minimise disputes at claims time, which is clearly in the consumer's interest. That is essentially what we are trying to do.

In the case you mentioned regarding mental health, I think that, as an industry, we recognise that we need to improve the underwriting processes as they pertain to mental health. It is—

Senator O'NEILL: Improve for what purpose? I know that it is a hit you are taking in terms of mental health claims, but there should be an improvement also for people, for your customers.

Mr Bailey: Yes, I think a clearer and more tailored process for customers is absolutely right. Questions framed in the right way as being able to—as I said, we would consider aspects like the timing and the nature of any condition, treatment, the time elapsed since then et cetera, but we can clearly improve the process. Indeed, we have—

Senator O'NEILL: Improve it in what way? Improve in your favour or improve in the favour of your clients? Because there is a tension—

Mr Bailey: I think it is both.

Mr Anderson: I think it is both. As a person who has applied for insurance, if I have a relevant health history that the insurer wants to know about, I think it is absolutely appropriate that they should find that out so that they can underwrite me up-front, and therefore I do not have uncertainty at time of claim. Everything is disclosed. That is really when it works well. In my opinion—

Senator O'NEILL: What are the implications of that for doctors in terms of making notes and observations about their clients? One doctor sees a patient and does not even disclose to the patient that they think they might be suffering from depression. They just give them a tablet; there is a trusting thing and they do not disclose that. You read it; you find out about it. That could have a terrible impact on that person. You are acting in place of a doctor. A doctor might have observed somebody and said, 'They appeared to be stressed.' That might then lead to a denial or an increase in the premium.

Mr Anderson: If we were not certain or it was not—as you say, in the vast majority of cases that we do see, people usually go to the doctor and say, 'Look, I'm not sleeping well; I've got work stress,' or whatever a scenario might be. If there is any uncertainty in the medical record, we will simply speak to the doctor and find out: what is the story? What is going on? With the vast majority, though—that we see, anyway—people do disclose. We get the information and we apply whatever we feel are appropriate terms to give people cover. And, as I said previously, of people who disclose a history of any mental illness—or mental health, I should say, because it is a fairly broad section—80 per cent are offered cover.

I know we are going to stay on this one, but to go back to previously, with looking at mental health in isolation at the underwriting time, the numbers I have for that 12-month period are that, for TPD, 3.5 per cent were

declined due purely to mental health—that was the only reason for the decline. For income protection, 5.5 per cent were declined due to mental health in isolation.

Senator O'NEILL: Okay. What is the definition of mental health? You use the words 'work stress'. I do not think you would insure any senator or anybody who is working here today if we talked about stress.

Mr Bailey: Through the underwriting process, we do look at the full continuum of conditions. For example, clearly that could be situational or medical.

Senator O'NEILL: Or it could be your language practice that you call stress just a busy workplace.

Mr Anderson: Absolutely.

Mr Bailey: That is why we would look into the situation in detail to make sure we are making an appropriate assessment.

Senator O'NEILL: Have you had any discussions with the Royal Australian College of General Practitioners or the AMA about the pressure that these practices apply to doctors in terms of breaching the patient-doctor confidentiality agreements?

Mr Anderson: No, I am not aware of any discussions.

Senator O'NEILL: Are you aware of the industry initiating or participating in conversations with those important representative bodies around these matters?

Mr Bailey: I believe that may be happening through the FSC.

Senator O'NEILL: When did you become aware of that?

Mr Bailey: I would need to take that on notice.

Senator O'NEILL: If you could take on notice when that has occurred and any details of those sorts of conversations, that would be very interesting for the committee. Probably I will have more questions on notice around a whole lot of matters.

Senator KETTER: Chair, I have a couple of questions regarding genetic information. Being a Swiss company, do you have some experience with what is happening in the Swiss jurisdiction?

Mr Bailey: I do not, but I am happy to provide it on notice.

Senator KETTER: Okay. In Australia, we do not have any legislation as such. You comply with the FSC genetic testing standard, No. 11, which allows you to access existing genetic test results. In terms of diagnostic genetic testing, perhaps you could explain to me what type of genetic testing information you are able to access or request.

Mr Bailey: This is so rare. I am happy to take that on notice. We do not request any genetic testing information at the point of underwriting. It is obviously an evolving and developing field. I believe it is extremely rare that anything of that nature would be disclosed at underwriting time. I am happy to provide more information on notice.

Mr Anderson: It would be a very, very small percentage, and I am not aware of the specifics. But I think, for conditions such as Huntington's chorea or haemochromatosis, the genetic tests—just from my reading as a member of the general public, not as a chief operating officer of Zurich—are getting more specific in the results, and therefore they are a little bit more accurate with regard to a person being likely to or not likely to have such conditions. I am not aware of genetic tests that are used in Australia with regard to, for example, having a greater risk of heart disease or cancer—other than family history, of course, which is not a genetic test.

Senator KETTER: Right. In regard to family history, I understand that only first-degree relatives can be inquired about for underwriting purposes. Is that your understanding?

Mr Anderson: That is my understanding, yes.

Senator KETTER: I understand these standards are being reviewed by the FSC working group. I am not sure where that is up to. Are you participating in that?

Mr Bailey: We are obviously members of the FSC and a number of the working groups. I am not so close to this particular working group. I believe there were some updates to the standards just recently, and certainly we comply with those. It is clearly a topic which I think is going to require further debate as this area evolves.

Senator KETTER: Do you believe Australia should have legislation similar to that in other countries that prohibits the use of genetic information by insurers in setting premium levels?

Mr Bailey: I do not really have a view on that at this stage.

Senator KETTER: I understand in some countries there are developments where insurers are paying for genetic testing as part of a wellness program. Is this happening in any of the jurisdictions where you operate, to your knowledge?

Mr Bailey: Zurich operates in a great number of markets globally, so it is not something I am specifically aware of it. Again, obviously, I am happy to—

Senator KETTER: Could you take that on notice?

Mr Bailey: provide you any information on that. Certainly.

Senator KETTER: Thank you.

Senator O'NEILL: I have a more specific question on that issue. I have had correspondence with a family who have had a child diagnosed with autism. I understand that there is a test called a DNA microarray that is now considered a standard test for developmental delay, and gene deletion or duplication matters. This information is actually available and, I understand from this correspondence, is already impacting people in terms of exclusion from insurance. We are talking about young children who are looking at a lifetime of not being able to get insurance. Are you aware of that?

Mr Bailey: I am not aware of that.

Mr Anderson: I have never heard of that, sorry, no.

Senator O'NEILL: Okay. It might be something of interest, because the evidence we have received so far is telling us that this is something in the future, but we are actually hearing about real-life, real-time experiences and reports of exclusion based on current testing that is required to be able to provide a treatment. I guess it is the same sort of thing as mental health: 'Do I avoid getting a test, because I do not want to have to disclose something that means I am not going to get insurance.' We have heard evidence of parents of people with mental illness doing anything they can to avoid getting a claim put through from a doctor that indicates mental ill-health, because of the insurance implications. Does this concern you, and what processes do you have in place to deal with this emerging problem?

Mr Bailey: I would need to look into the specific question. I am happy to come back to you on notice about that.

Senator O'NEILL: Mr Bailey, did I hear you say that you are part of the working group of the FSC around life insurance?

Mr Bailey: Zurich is a member of the FSC. As with all of the other life companies, we are represented on the Life Board Committee, yes.

Senator O'NEILL: Can I encourage you to raise those complex questions? I know that there has been a lot of talk about back office and changes with regard to remuneration in the sales et cetera, but I think there are other very big ethical questions on the horizon that also need to be attended to.

Mr Bailey: I am certainly happy to raise that.

Senator O'NEILL: Thank you.

CHAIR: We have run out of time. The senators might write to you with some specific examples to assist the FSC in that area. Thank you for appearing today. If you have taken questions on notice, the answers should be provided to the secretariat by 9 June 2017. They will provide you with a list of those questions on notice from the *Hansard*. Again, thank you for attending the hearing and for your evidence today. The committee will now suspend for lunch.

Proceedings suspended from 12:30 to 13:16

MORPHY, Mr Timothy, Chief Executive Officer and Director, MedHealth Pty Ltd

CHAIR: I now reopen this inquiry into the life insurance industry. The committee welcomes MedHealth Pty Ltd. I remind committee members and witnesses that, while this is a public hearing, care should be taken to protect the privacy of individuals and that arguments should be made without naming individuals. I now invite you to make a short opening statement, and, at the conclusion of your remarks, I will invite members of the committee to put questions to you.

Mr Morphy: On behalf of MedHealth, I appreciate the opportunity to appear before this joint committee. At the outset, I would like to highlight that MedHealth does not directly participate in the life insurance industry—that is, MedHealth is not an insurance company; does not market, sell or manage any insurance-related products; and does not determine any insurance claim in any jurisdiction or scheme, including the life insurance market. Rather, MedHealth provides a range of health risk management services and related medical support services. These are utilised by a wide range of industries and organisations, which include the life insurance industry.

By way of a brief background, MedHealth was originally founded as mlcoa in Melbourne in 1986. Today, MedHealth owns and operates six core business units in Australia, each with dedicated management and operational teams. MedHealth is committed to being a quality provider in relation to all of its services, and is the only provider of its type that we understand is quadruple ISO certified. This is outlined in our submission.

A particular service provided by several MedHealth business units that I understand is of interest to the committee is the facilitation of independent medical examinations, often referred to as IMEs. An IME is an assessment of a person by an independent medical specialist to form an independent expert clinical opinion regarding an aspect or aspects of that person's injury or medical condition. MedHealth does not directly provide IMEs. Rather, it facilitates the provision of an IME from an independent medical specialist for and on behalf of a requesting organisation. IMEs are requested in the life insurance market by life insurance companies, law firms representing life insurance companies, and law firms representing individual clients with a life insurance product. Once the IME has been completed, it is then provided to the requesting organisation as one of many inputs into its decision regarding a claim. Neither the independent medical specialist conducting the IME nor MedHealth have any part or any role in the decision-making process regarding that claim.

IMEs are an example of expert opinion evidence. When an independent medical specialist is preparing an IME, they are preparing expert medical opinion evidence that can ultimately be relied upon by a court. There are extensive rules governing expert opinion evidence. In addition to the serious criminal sanctions that apply to any witness who intentionally misleads a court, a finding that an independent medical specialist had intentionally sought to mislead a court would be expected to render them liable to be disciplined, if not struck off, for professional misconduct. In this regard, MedHealth and independent medical specialists take their responsibilities and obligations very seriously. A quality IME, and one of high integrity, can be regarded as one that: firstly, where possible, provides a clear answer or answers to the questions posed by the requesting party; secondly, clearly articulates the reasoning, the logic and any supporting evidence relied on by the independent medical specialist, and this includes referencing any relevant studies, journals or peer-reviewed articles; and, finally, is able to withstand scrutiny in a court of law.

In summary, MedHealth provides a range of services, including facilitating IMEs. We do not, ourselves, provide IMEs; this is done by the independent medical specialist. We are not an insurance company and we do not determine any claims in any jurisdiction, including the life insurance industry. We are committed to providing quality services and quality processes, an example of such being our ISO certification. With that said, Mr Chairman, I am happy to take any questions.

CHAIR: Thank you. Senator Williams.

Senator WILLIAMS: Thanks, Mr Morphy, for your time here. Who owns MedHealth?

Mr Morphy: MedHealth is owned by a US-based company called ExamWorks.

Senator WILLIAMS: What is it called?

Mr Morphy: ExamWorks.

Senator WILLIAMS: Is ExamWorks a publicly-listed company in America?

Mr Morphy: It is privately owned.

Senator WILLIAMS: So a privately-owned company in America owns MedHealth, which is based in Australia—is that correct?

Mr Morphy: Yes, we are the Australian operations of the international—

Senator WILLIAMS: Wholly and solely owned by this ExamWorks in America?

Mr Morphy: Yes.

CHAIR: Can I interrupt? Mr Morphy, can the secretariat get a copy of your opening statement?

Mr Morphy: Certainly.

CHAIR: Thanks.

Senator WILLIAMS: And MedHealth owns—do you pronounce it 'Melcoa'?

Mr Morphy: Mlcoa is one of the operating companies that—

Senator WILLIAMS: What do you call it—'Melcoa'?

Mr Morphy: We use the abbreviation, of M-L-C-O-A, which is a mouthful I know, but that is what we say.

Senator WILLIAMS: We live on acronyms around here, I can tell you.

Mr Morphy: I know; we have one.

Senator WILLIAMS: So you own mlcoa, Next Health, ASSESS, MyClinic and CaseWorks—correct?

Mr Morphy: Yes, we operate all those businesses.

Senator WILLIAMS: So mlcoa, Next Health, ASSESS and MyClinic are IME organisers, are they?

Mr Morphy: Facilitators. That is one of the things they do: they facilitate IMEs—

Senator WILLIAMS: Give us an example. Say I am an insurance company—no; let us just turn it around a bit.

CHAIR: You don't look anything like one!

Senator WILLIAMS: I am too poor to be one! Say I put a claim in to my insurance company for trauma. I have had a heart attack. Let us say I am with Zurich. Zurich comes to, say, mlcoa, and says, 'Will you organise a doctor to check Williams out.'

Mr Morphy: This is one avenue that could be. The insurer would approach us and say that they would believe that, in determining your claim or their way forward, they would benefit from the opinion of an independent medical expert. They would contact us and explain the circumstances. So let us say it was a heart condition. Then it would be the right specialist—right geography; right credentials. We would suggest to them the options that we have or the doctors available that could meet those requirements. They would select one and then we would go into a process of facilitating the IME, which would include receiving all the relevant information for the doctor to review, prior to an examination and an interview. Then the doctor would prepare a report, and reference and look for any independent evidence that they wanted to assemble in terms of research. That report would be prepared. It would be QA'd. It would be reviewed by the doctor, finally approved by the doctor, and returned as an IME to the requesting party.

Senator WILLIAMS: So you obviously have a list of doctors—

Mr Morphy: Yes, we have an extensive—

Senator WILLIAMS: And they could be cardiologists; they might be orthopaedic surgeons.

Mr Morphy: Absolutely.

Senator WILLIAMS: They might be ophthalmologists—they could be anything.

Mr Morphy: Yes. I would say we would cover virtually every medical specialty and subspecialty. Part of the proposition that we offer is that we have this extensive panel of experts.

Senator WILLIAMS: When you put these doctors on the list, do you get a share of their fee? I was told that if you have got a doctor on your list, the doctor gets 70 per cent of their fee and you get 30—is that correct?

Mr Morphy: Well, I would say it is a case-by-case negotiation. So, basically, the doctor agrees to be represented by us, to come onto our panel, and we would agree with that doctor, up-front, the commercial terms of that arrangement, but that would vary, and does vary, by doctor and jurisdiction and specialty. There is no fixed model there.

Senator WILLIAMS: But in some cases you would get—

Mr Morphy: Yes, we would certainly take, yes—

Senator WILLIAMS: a share of the doctor's payments?

Mr Morphy: In terms of the payment being made by the requesting party, we would receive a part of that for our services.

Senator WILLIAMS: You would also get paid by the insurance company?

Mr Morphy: Depending on who is requesting it. Typically the people requesting it are responsible for paying it.

Senator WILLIAMS: I am doing a claim with Zurich. Zurich gets you to do the work. They pay you and you also get a slice of the doctor's remuneration.

Mr Morphy: We think about it as a set fee. We share in that fee with the doctor. They take an amount. We take an amount for the services that we provide.

Senator WILLIAMS: Who sets that fee?

Mr Morphy: The fee—we charge the insurer.

Senator WILLIAMS: The doctor's fee.

Mr Morphy: That is a case-by-case negotiation.

Senator WILLIAMS: For example, I have had a heart attack. I go to a cardiologist. How does that doctor charge me? I put in a claim.

Mr Morphy: That doctor does not charge you. The relationship is between the requesting party and the doctor who prepares the IME. The doctor has nothing to do with the treatment—

Senator WILLIAMS: So Medicare would have some payment and also the doctor for doing their job?

Mr Morphy: No. It is completely outside. It is not a treatment relationship at all, and that is a very important thing to note.

Senator WILLIAMS: I am glad you clarified that. So the insurance company pays the doctor?

Mr Morphy: In our case, the insurance company pays us. We then pass on the agreed commercial amount, which we have agreed with the doctor. It is their share of that.

Senator WILLIAMS: mlcoa, Next Health Group, ASSESS Medical Group, mi-Clinic are all IME providers. Is that a good way to describe them?

Mr Morphy: Amongst other things, that is certainly a key part of their businesses.

Senator WILLIAMS: CaseWorks says their job is simplifying the claims process. Is that correct? CaseWorks is obviously different from the other companies.

Mr Morphy: CaseWorks does not provide IMEs, but it works around insurance industries, providing other services that are valued by organisations.

Senator WILLIAMS: Explain the job of CaseWorks. They work in the insurance companies, do they?

Mr Morphy: What we identified through CaseWorks—and this was simply a market opportunity—is that we looked at the claims process in the life market and we identified that there were some particularly frustrating aspects to it. One of those aspects frustrating everybody was the completion of the claims form. So the initial product and the key product—

Senator WILLIAMS: So, if I am going to put a claim in and I am having trouble putting it in, I would come to CaseWorks for advice?

Mr Morphy: Typically how it happens is that if you contacted an insurer and said, 'I would like to make a claim,' they would send you out a claim form. At that point, there is a lot of frustration for everybody in terms of completing it. The proposition is that we allowed the insurer to say, 'With your consent, we can pass your claim information to CaseWorks and they will arrange for a nurse to go to a place of your choosing, typically your home, and they will assist you in completing the claim form.

Senator WILLIAMS: So the insurance company would employ CaseWorks to help me put the claim form in.

Mr Morphy: Engage us. Yes. They pay for the service. We would attend. We would help you complete the claim form and then we would submit the claim form back to the insurer. We tracked very carefully the satisfaction with this, and it was a highly satisfying experience for everybody. The insurer had the claim form completed quickly, and they could return it to make a fast decision. The claimant had assistance in completing the claim form. Everybody was very satisfied with that service.

Senator WILLIAMS: Would CaseWorks recommend an IME?

Mr Morphy: No. With CaseWorks, that service is purely an assistance service. It is a nurse that is there purely to assist the person completing the claim form. The nurse would not even know what an IME was, and certainly it is not part of their role at all.

Senator WILLIAMS: One concern I have—and I have to be very careful how I put this to you—is that I have spoken to two doctors and they have told me that their reports have been altered.

Mr Morphy: Altered?

Senator WILLIAMS: Yes. I find that very concerning. They will not come before this committee. They are lying underground. They are gun shy et cetera. Have you ever heard of any cases where doctors' reports have been altered by a centre company, an organising company, such as yours?

Mr Morphy: No. And we would have absolutely no desire to do that. That would be a ridiculous thing for us to do in the first place.

Senator WILLIAMS: It would be fraud.

Mr Morphy: It would be fraud—

Senator WILLIAMS: It would be a criminal act.

Mr Morphy: It would be a ridiculous thing. Ultimately, the report is the report of the doctor. They are the one that ultimately would be standing in court to defend it. Yes, it would be fraud and it would be an absurd thing for anyone to engage in. It is the doctor's report.

Senator WILLIAMS: I hope these two doctors I have spoken to have been lying to me but I doubt they have.

Mr Morphy: I cannot comment on that. I would not say it. Certainly it is not something—

Senator WILLIAMS: I am not going to name them. I am not going to throw them over a cliff without a parachute, but that is one of the concerns I have. I hope through this inquiry that your industry, along with other IMEs, gets a clear message that if there are ever any tamperings with doctors' reports or changing of reports, I am sure there will be serious consequences.

Mr Morphy: I totally agree with you. It would be outrageous if that was being done.

Senator WILLIAMS: I will bring you the story investigated by Adele Ferguson some months back. Life insurance is supposed to bring relief to the lives of sick and dying people.

MLCOA came to my attention when a reader sent in an internal memo from MLCOA's operation in Tasmania to staff and independent consulting doctors. It was published in 2014 but its contents ratchet up the debate on the growing army of IMEs.

It refers to a number of issues, including the return of files. "All doctors are required to check their rooms/homes/offices etc for any MLCOA Hobart patient files that have not been returned to us." It goes on to say "we have a lot of loose papers relating to supplementary requests etc that are awaiting return of the file". It says when removing confidential patient files from "our rooms" ask for TNT or Express Post bag for return of files.

Do you know anything of this?

Mr Morphy: I am certainly aware of the article and I am aware of the internal memo. I think the particular manager who wrote that, had they known that would be subject to public scrutiny, might have been a lot more careful with their language. But it is the case that doctors do require access to material to prepare for examinations, and it can be extensive. We have protocols and processes to make sure that that paper is checked out and returned to us. But, yes, that is the case that they do need to get it. We follow them up all the time to return it if it was not naturally returned. So we are aware of that.

Senator WILLIAMS: You are aware of that memo. When did you become aware of it? Was it before or after the story?

Mr Morphy: After the story, or as the story was prepared, if you like.

Senator WILLIAMS: When Adele Ferguson spoke to you?

Mr Morphy: Yes, I was asked to comment on it .

Senator WILLIAMS: Yes. She quotes:

The head of MedHeath Tim Morphy said he hadn't seen the memo but it would have been a general reminder to doctors to be careful with files. He said it is unavoidable at times that doctors have to have access to files and that means sometimes taking them away. "It goes on in the medical world all the time."

In a follow-up statement he said: "Given the volume of documentation, independent consultant doctors often request to review this material either in their clinical rooms or at their home offices. This naturally demands a rigorous process for document security which includes tracking documents to ensure their receipt and return. We utilise Australia Post traceable services to assist consultant doctors in returning documents. Consultant doctors are fully apprised of their obligations with regard to document security."

The memo also refers to "court matters" where it says "When having to present at court in relation to a person you have assessed, it is important you are in possession of your final report which is held by MLCOA and not the report you may have

on laptops etc. The report held by the client after amendments in Q&A can differ from the report you have stored on your PCs etc which may not be the draft you have before the amendments were made".

So this memo is telling them that when they go to court, do not take your draft report take the final report from mlcoa.

Mr Morphy: As I say—

Senator WILLIAMS: I will give you a chance in a minute. Mr Fullegar said: '

… MLCOA sees the need to send out a note to examiners not only to warn them they may end up in court, but how to prepare for it.

Can you explain all of this please?

Mr Morphy: I can say every independent medical examiner understands there is a possibility that they could be asked to defend their report in court—that is in the forefront of their mind—in front of a judge and in front of their peers.

Senator WILLIAMS: Let me stop you there. I am a doctor. Mlcoa has come to me and said, 'I want you to do a report on this person who has put a claim in.' I write the report and I send it to you—to mlcoa. Right? I give the report to mlcoa. Let's say I am working at mlcoa—

Mr Morphy: There is a quality-assured process of a draft being prepared and being reviewed by the doctor and our quality assurance staff, but the final version of the report is always, without exception, reviewed, approved and signed by the doctor. One of the services that we offer—

Senator WILLIAMS: Oh yes.

Mr Morphy: is to make sure we store the completed and signed report. That is something that we guarantee, if the doctor does need to access that report: 'Come to us and ask, because we always have your final report.'

Senator WILLIAMS: On one of those reports that the doctor prepares that goes in the final report, how many pages would be in that report?

Mr Morphy: It could vary tremendously.

Senator WILLIAMS: Would it be 15, 20, 40?

Mr Morphy: It could be. I do not think they would typically be that long, but they would typically be between five and 15 pages. They can be quite substantial, depending on what is being asked to be done.

Senator WILLIAMS: The doctor that I met with told me that they flick to the last page and say, 'Sign here,' and many times the doctors sign those reports and have not read them word for word through each page. Perhaps we need a situation where, just like when you go to your solicitor to do something, you have to initial each page after you have read it. That is what a doctor told me—that in too many cases they might have a dozen reports; they have been finalised; they just sign the back page and move on. Have you ever heard of that happening?

Mr Morphy: I would be amazed if a doctor—as I say, this responsibility falls on the doctor. If a doctor is signing any report without reviewing it, I would think that would be a very odd situation. I am not aware of doctors being so—

Senator WILLIAMS: I am just telling you that that is what a doctor told me.

Mr Morphy: That they are so lackadaisical about what they are signing that they do not read it?

Senator WILLIAMS: I am just telling you. That is what a doctor told me. They have done the report. They get the summary, the 15 pages. They go to the back page and sign it without reading it. So perhaps we need a situation where they must acknowledge that they have read every page. That is what I am suggesting.

Mr Morphy: By signing it, I would argue, they are acknowledging that. But if you—

Senator WILLIAMS: You are arguing that by signing it they are acknowledging that everything in it is true in fact. I am telling you that a doctor has told me that they rush through them, sign the back page and move on. We will agree to disagree. You are saying it has been done perfectly; when they sign it, they have read every word, word for word. I am saying that a doctor has told me that is not the case. So we have a problem.

Mr Morphy: If there is a doctor that does not take their obligation seriously, they would not be someone welcome in our organisation.

Senator WILLIAMS: Why would you tell a doctor to take the final report to court and not the report that they prepared? Run that past me again, please.

Mr Morphy: The language used there is regrettable. What that was saying is that we acknowledge that there are drafts prepared on a journey to a final report being reviewed and signed by the doctor. We always keep that

final signed report. The concern was that a doctor might take a draft, not the final report. Again, if the language had been tighter, that is what they were trying to say: 'Make sure you come to us for your final signed report. Make sure you don't have a draft.' It is as simple as that.

Senator WILLIAMS: If a doctor drafts a report, and it might be 15 pages, and hands it to you, what would be changed in it? Explain to the committee the difference between the doctor's draft report and the final report.

Mr Morphy: Very simply, the doctor does all their background reading. They sit down and they do an examination and an interview. Then they would sit there, and a lot of them just, through a dictaphone, draft a very general report, really to get their thoughts down on a page. They then ask for that first transcribed report back, and then they would start reviewing it and refining it. As I said, these are 10- to 15-page documents. They are complicated, and they want to be incredibly precise. So there is a process by which the doctor would start the process of preparing their report. Once the doctor is happy with it, we have a quality assurance process where we check it and read it for its accuracy in terms of typographical errors, completeness and its clarity. If there are any suggestions, they would go back to the doctor, specifically highlighted. The doctor would look at them, agree or disagree, and then the report is finalised. It goes back to the doctor. They review it and they sign it. That is the process. This is not a process that has not gone through extensive scrutiny. This is an ISO-certified quality assurance process, and it is all designed to make sure that that report is 100 per cent correct and the doctor is 100 per cent confident that the end result is perfect. That is what it is there for.

Senator WILLIAMS: I agree with you totally, Mr Morphy, but what I am concerned about is when I speak to two doctors who tell me that reports have been tampered with and altered. Now, clearly, you are right, and the doctors I have spoken to are wrong and are lying to me! I do not think that is the case. If you look around your community, who do you trust? Probably not your local politician. But you trust your pharmacist, your chemist; you trust your doctor et cetera. When I meet with these doctors and they talk to me, and I talk to them over the phone, what reason would I have to say they are lying to me? This is the conflict we have in this whole inquiry, Chair.

Mr Morphy: It is very difficult for me to comment on unknown sources. All I can do is point to what we do and the seriousness with which doctors are being held to account in what they are doing. Ultimately, it is a doctor who is standing up in court defending what they have done. We have zero interest, reason or logic to want to alter that. That is not our job. Why would anyone want to do that? It does not make sense to me. There is no logic for it.

Senator WILLIAMS: Why would a doctor want to meet with me in person and bring documents with him and show me the documents and tell me about how they have been altered? I promised him I would cover for him and his name would never be mentioned—I would never call him to the committee or whatever—to protect his future. Why would a doctor do that to me?

Mr Morphy: Without knowing the circumstances or who it is, I have no idea. Perhaps they have an axe to grind with us or an organisation. How could I possibly comment? All I can comment on—

Senator WILLIAMS: No, he did not have an axe to grind with you; I can promise you that.

Mr Morphy: All I can comment is: not only does that not happen, or we have no interest in it happening, but there is no reason or logic for us to do that. There is no benefit to us to do that. It just does not make sense. Why would we do that? How could that possibly—

Senator WILLIAMS: Perhaps the inference to me was this. When you are working for the insurance companies, (1) it may save the insurance company money, and (2) it may guarantee you more work because you are seen to be a company that is working for the insurance company and saving money. That is what a cynical person may say or think.

Mr Morphy: Again, you just need to think through the logic of that. The insurance companies come to us for a quality IME. What is a quality IME? It is one that ultimately stands up to scrutiny in court in front of their peers. That is what they want to assist them in their decision making. To just give them something they might want to hear—what do they do with that? Just do not ask for an IME. It is just not a logical proposition.

Senator WILLIAMS: But no doubt you have an interest in growing your business. You want to return profits back to America, which owns you. Since you have been here, you have bought several businesses in Australia. Is that correct?

Mr Morphy: We have acquired businesses, yes.

Senator WILLIAMS: Yes, exactly. Where more than one independent medical examiner is used for a claim, what is the reason for this?

Mr Morphy: It could be multiple reasons. The most obvious one would be that there are different specialties required.

Senator WILLIAMS: Explain that. Go on. Different specialties required? Continue with more explanation on that, please.

Mr Morphy: Okay. You have been in an accident. You have suffered a range of injuries that require different specialists to look at them—a shoulder injury, a knee injury. You have suffered psychological damage; there is a psychiatrist involved. There could be three, four or five specialists required to look at different injuries. It is not uncommon.

Senator WILLIAMS: Okay. Was there an increase in revenue for companies such as MedHealth over the past five years? Has your company grown much in its revenue?

Mr Morphy: We have been a successful company, and we have grown, yes.

Senator WILLIAMS: How do you define 'successful company'? Can you give the committee some idea of how successful you have been?

Mr Morphy: As a private company, we do not discuss our commercial performance publicly, Senator.

Senator WILLIAMS: I wish my farm was as successful as your business. What avenues are available for independent medical examiners to report any wrongdoing by insurers—or any wrongdoing in the whole IME industry?

Mr Morphy: They are members of professional bodies, and they can also go to any regulatory body that sits above an insurance company. They could report it to us, and we would deal with it as well.

Senator WILLIAMS: They could report it to ASIC?

Mr Morphy: They could report it to ASIC. They could report it to the police.

Senator WILLIAMS: They could report it to APRA?

Mr Morphy: APRA—I think there are a range of options.

Senator WILLIAMS: Or they could tell a politician.

Mr Morphy: What's that?

Senator WILLIAMS: They could tell me.

CHAIR: Deb—Senator O'Neill.

Senator O'NEILL: You're welcome—

Senator WILLIAMS: How demeaning, Deb!

Senator O'NEILL: It is all right. We have been friends a bit too long probably.

CHAIR: Just for the record.

Senator O'NEILL: You could imagine, Mr Morphy, that Senator Williams's conversations with these doctors have exercised the minds of the committee quite seriously. I would like to think that your business model is perfect and not flawed in any way and that all the people who participate in it do it with good intention and nothing unethical happens, but life has probably taught me at this point in time that nothing is perfect and people do engage in practices that might be considered unethical when the full light of day is shone upon them.

We do not doubt Senator Williams' interactions with these doctors, who do claim very clearly that there has been tampering with their records. You have made quite a good argument for why that is not good for your organisation and not good for those people. But I put it to you that one of the incentives to perhaps not notice that practice of a change to a report could be to keep their job, as an independent medical examiner that is on your books, and simply ignore a change. That might not be material to them in any way, maybe they think it is not going to have any impact on the client, and they just let it go through.

Mr Morphy: I do not, Senator. I do not understand. Again, I go back to the point: what would be the logic of that? People, whether they are plaintiff lawyers or insurance companies, come to us for a quality IME, and ultimately what they are looking to understand is answers to questions, and they are looking for something that will withstand the scrutiny of court and peer review in a public forum. If they do not want that, then they do not ask for an IME. But that is what we do. They are looking for that support. So the whole idea that either a doctor would tell them something that is not that or we would alter it so it is not that is absurd, because why ask? What are they going to do with something where they are told, 'Here's an IME, but by the way it is not correct'? What do they do with that—rely on it or not rely on it? It is just quite absurd as a suggestion. I cannot say anything other than that. There is no logic to why we or a doctor would engage in that.

Senator O'NEILL: The point is that there might be situations where the doctors who are looking after the person who is making a claim have a very different view from the IME doctor.

Mr Morphy: I would acknowledge that there is sometimes disagreement about what an IME says between a treating doctor and an IME doctor—or amongst expert doctors there can be disagreement. But the concept that experts can disagree is not something that is foreign to the judicial system. That happens all the time. The idea in medicine that there cannot be differing opinions is not foreign either. Second opinions are something that are well understood as a concept. But, again, that to me is just more evidence that it is quite absurd for someone to suggest that there is a vested interest in not providing a quality IME. Why tell someone, whether it is, as I said, a plaintiff lawyer or an insurer or any interested party, something that is ultimately not true? What would they do with that if they cannot rely on it? It just does not make sense.

Senator O'NEILL: In your submission, Mr Morphy, there is a paragraph which says: 'A request for an IME is done by a letter of referral with a structured series of questions from the requesting party in relation to a person's injury or medical condition. These questions are put to the independent medical specialist for consideration and response to the requesting party.' And then, in the final sentence, it says: 'It is not'—and 'not' is in bold print and underlined—'the role of the independent medical specialist to formulate, propose, suggest or change the questions asked.' That really drew my attention. If you are asking for an independent medical expert but you are so determined to contain their capacity to respond to a case that they as a medical specialist are not allowed to 'formulate, propose, suggest or change' any of the questions, that seems to be very restrictive.

Mr Morphy: It is not restrictive; it is designed to be very precise. From the point of view of the medical specialist and our point of view, this is a very precise science. They are being asked to comment on very specific questions. They do not see their role as one to imagine what else could or should be asked. They respond—

Senator O'NEILL: They do not see that as their role, or you do not allow them to see that as their role by such a direction?

Mr Morphy: They do not see that as their role. They do not see doing that as their role. They see their role as being asked the questions and they will respond if they can. I think I also note in there that it is not uncommon for a specialist to say, 'I cannot answer that question,' based on either their own area of specialty or a lack of information, and they would be the first ones to say that. They are the ones that could be put in a position of having to defend a position they have taken, and they do not want to be exposed in open court, in front of their peers, for saying something that cannot be defended.

Senator O'NEILL: I have to say the notion of restricting it down to an absolutely specific question, rather than allowing a specialist with, I assume, decades of experience to have a fuller and more personal discernment capacity, is a concern for me.

Mr Morphy: Okay. I understand. Right now, this can be a question raised with medical specialists and referrers, but it is ultimately a question of the approach that they have taken being quite precise in focusing on the questions that are asked. They do not see it as their role to be creating or developing other questions.

Senator O'NEILL: What year did your company start here?

Mr Morphy: The original company, mlcoa, was founded in 1985—certainly well before my time. I have been with the company about four years in my current role.

Senator O'NEILL: Prior to that, what methodology are you aware of that was employed to get medical advice?

Mr Morphy: It was a similar methodology, I think. mlcoa was one of the original founding companies, if you like, of this industry. It has certainly gone through—

Senator O'NEILL: A series of iterations?

Mr Morphy: a series of evolution around how it has developed. Under its current ownership—and this is my involvement—the particular focus has been around the ISO certification to absolutely commit to the quality processes and subject yourself to external review around those processes.

Senator O'NEILL: I note that in your submission. Thank you. Your business has grown. You expressed to Senator Williams that you were not willing to disclose the degree of your success in dollar terms. Could you give it to us in terms of your percentage growth over the last couple of financial years.

Mr Morphy: I will take that on notice and come back to you on our growth. As I say, the principle is that we regard our commercial performance as commercial-in-confidence.

Senator O'NEILL: Going back to your statements that, on a case-by-case basis, the fees are negotiated and agreed with the doctor and remain commercial-in-confidence, could you just give us an indication of the range of remuneration a doctor might be able to receive for a single case.

Mr Morphy: The range would be from $500, $600 or $700 up to many thousands of dollars, depending on what they are being asked to do.

Senator O'NEILL: What would I get for $500 or $600? What sort of service is that?

Mr Morphy: There are some very short, specific questions in certain jurisdictions where it is a very focused, contained proposition that can be done relatively quickly and efficiently. For example, reviewing a permanent impairment as the only question might be something that is not as expensive as a detailed review of diagnosis, treatment paths and return to work frameworks, for example.

Senator O'NEILL: The second model that you just spoke about there—diagnosis, treatment, recovery pathways et cetera—is many thousands of dollars. What is the maximum dollar amount that you would expect?

Mr Morphy: There is no maximum or minimum. It is a moving—

Senator O'NEILL: Would it get to hundreds of thousands of dollars?

Mr Morphy: No.

Senator O'NEILL: Okay. I am just asking for a ballpark figure.

Mr Morphy: Two to three thousand dollars would be the maximum range that I would be aware of.

Senator O'NEILL: Roughly how long do you think it would take a doctor to do the $500 or $600 service with the limited questions that you referred to?

Mr Morphy: Again, that would vary tremendously, but it would take—if you allow for the reading of materials, the examination, the interview, preparing the draft report, any research that is required, review of drafts and finalisation of drafts—typically three or four hours of a doctor's time, or maybe a bit more.

Senator O'NEILL: And the $3,000 is presumably a sort of—

Mr Morphy: It can run into days of work for a doctor, depending on what it is.

Senator O'NEILL: And that is the fee that the doctor receives?

Mr Morphy: Yes.

Senator O'NEILL: And, in addition to that, you would receive money on top. I assume for a $500 or $600—

Mr Morphy: We would receive a share of the overall fee that is being paid for the IME.

Senator O'NEILL: Is the fee that you have just given me with or without your part of it?

Mr Morphy: That is without. Sorry, I took you as referring to what the doctor would receive.

Senator O'NEILL: So you would receive moneys in addition?

Mr Morphy: Yes, as a share.

Senator O'NEILL: So the bill to the insurance company for the $3,000 service could be something like $4,000?

Mr Morphy: It could range, yes.

Senator O'NEILL: Could you charge an insurance company more than that for a $3,000 service?

Mr Morphy: The fee that the client charges is negotiated and agreed up-front with each client. As to how the amount is split between us and the doctor, that is a completely commercial matter that the insurance company is not made aware of or involved in. It is not a question or an issue for them. It is a commercial relationship between us and the doctor.

Senator O'NEILL: I am assuming there is enough of a gap in there for you to be reasonably profitable in what you are doing. Are there standard industry-set fees for these services?

Mr Morphy: It varies tremendously. I think the latest trend is that these services are secured by major referrers under a tender where they are very specific about what they expect in terms of the process they want supported and the prices they are prepared to charge. They are often quite complex formulas because they recognise the fact that there are different specialties, there are different geographies that are harder to service and there are different complexities in exams. But, yes, there is a broad fee structure—

Senator O'NEILL: Are you the major supplier who would respond to a request from an insurance company?

Mr Morphy: We are one of them. There are a range. It is a competitive market. There are a number of organisations that provide services of our type. We like to think we are the premium provider in the market.

Senator O'NEILL: Premium because of the quality of your service or the scale?

Mr Morphy: The quality of what we provide and the breadth of our panel. We are able to arrange a range of doctors in a range of specialities across all geographies and jurisdictions.

Senator O'NEILL: How would you assess your market share?

Mr Morphy: Unfortunately, there is no public way to assess that. I would be purely guessing. I would hate to speculate.

Senator O'NEILL: How many competitors do you think you have in the field?

Mr Morphy: Again, it is hard to know, but I would say there would be 30 to 40 competitors of our nature, plus there are doctors who do this individually which could run into many hundreds of doctors who are prepared to do this work individually. Again, I do not—

Senator O'NEILL: Doctors could provide that service across a range of different—

Mr Morphy: Certainly doctors work with a range of different providers. They are not restricted to just be with one.

Senator O'NEILL: For some of the doctors who are doing this, could this become their sole source of employment?

Mr Morphy: It could certainly become a significant part of their employment, yes.

Senator O'NEILL: If that became their sole source of employment, they might become quite needy of that income at a certain point of their professional lives. They would want to keep the relationship well established.

Mr Morphy: They would want to be providing quality IMEs if they wanted to be in that industry, yes.

Senator O'NEILL: In terms of the doctors from whom you require services, what is the highest amount that you have paid to one of those doctors in the course of a financial year?

Mr Morphy: That is very commercially sensitive material and I am not prepared to discuss individual arrangements with doctors. I am sure you can understand—

Senator O'NEILL: I am not asking for any specific name or any—

Mr Morphy: The fact of what is paid is highly sensitive so I am not prepared to answer that.

Senator O'NEILL: Can we just hold for a second.

CHAIR: While we are holding, Mr Morphy, is there any connection between MLC and mlcoa when it started.

Mr Morphy: No, they are completely separate companies. There is no relationship.

CHAIR: You say you have 12 different types of MedHealth customers listed—you can take this on notice if you like—but out of the 12 there is only one with regard to treating medical practitioners and their patients. What percentage of those, out of your total business, would—

Mr Morphy: That is a relatively small part of the business.

CHAIR: And the majority of people who come to you would dispute a claim?

Mr Morphy: No, not at all. The vast majority of cases are as input to resolving or deciding a claim up-front.

CHAIR: Why couldn't an insurance company or any of these customers of yours just go straight to a specialist and ask them to do the job?

Mr Morphy: They can and they do.

Senator O'NEILL: While the secretariat is getting some advice about that question, I will ask you to reconsider providing that indication of a quantum of how much a person could earn if this was the sole thing that they invested in doing.

Mr Morphy: I am saying that I think it is commercially sensitive as to how much doctors would earn or could earn, or how much we would pay them. On the basis of confidentiality, I do not want to answer that question.

Senator O'NEILL: Okay. We might explore that a little more. In your evidence this afternoon, you talked about the doctor sending in his report. You went through some detail with Senator Williams about the number of drafts. You also mention a quality assurance review. How many drafts would doctors provide to you?

Mr Morphy: Again, it varies tremendously, driven mainly on the complexity and the length of the report being written, and also the experience of the doctor doing it. Some doctors are able to produce very robust drafts

very quickly from a dictation. Some require multiple drafts to refine their position, if they want to a comfortable signing off. Again, it varies tremendously.

Senator O'NEILL: When you say 'a robust draft', what you mean by that descriptor?

Mr Morphy: Some doctors are able to dictate, from their examination and notes, quite a complete report that does not require them to do a lot of refining. Some doctors require or like to refine their reports a lot.

Senator O'NEILL: Why would a doctor be sending you a draft that is not robust?

Mr Morphy: We arrange transcription of the report. It is one of the services we do. The doctor will dictate the report and we will take the dictation, prepare the draft verbatim of what they have dictated and give it back to them. That is the first draft.

Senator O'NEILL: But then what happens to it? Do you send them some directions about needing to make it more robust?

Mr Morphy: No. The doctor will then refine their position—make any changes. Again, that may cycle two or three times as they are saying, 'I'm comfortable with it.' We will then go in and changes will be made through transcription. It will be quality assured, where a quality assurance officer will read it and make any observations about simple things like grammar, layout or typographical errors. They will review it for its consistency and clarity. That will be provided back to the doctor. The doctor will review that, agree or disagree with that, respond to it or not—as is their choice—and then they will say, 'This report can now be finalised.' It goes in. A final version is prepared and given back to the doctor. The doctor reviews it, signs it and says, 'This report can now be issued.'

Senator O'NEILL: The changes that you are talking about to make it more robust would seem to be substantive changes rather than small changes of grammar and syntax.

Mr Morphy: No. Let me be clear: it is reviewed for its consistency and clarity. There is no feedback given whatsoever on medical terms or medical issues. It is purely reviewing it from the point of view of an independent reader—the quality assurance officer—for its clarity and consistency. That feedback is given to the doctor. The doctor then responds to that in the best way they feel fit. Ultimately, it is their report.

Senator O'NEILL: Just so I am clear so far: these draft documents, which could be five to 15 pages on average, could go through a number of iterations—

Mr Morphy: They could, yes.

Senator O'NEILL: where they are reviewed by a quality assurance officer who has what qualifications?

Mr Morphy: All of our quality assurance officers go through an internal training program to be allowed to be a quality officer. Again, this is all part of the ISO standard that we work to.

Senator O'NEILL: What internal training do you give them and what qualifications do they have to have to do this?

Mr Morphy: Most of them have industry experience, but we have an internal training—

Senator O'NEILL: In the industry as what?

Mr Morphy: They have been involved in the insurance industry. Most of them have experience having seen and understood an IME. That is typical. Some of them have a legal background or a nursing background, for example. What they all go through is—

Senator WILLIAMS: A nursing background?

Mr Morphy: A nursing background—they have been a nurse.

Senator O'NEILL: How many of these officers do you have and are they all degree qualified?

Mr Morphy: They are not necessarily degree qualified, but they are experienced. I would need to take the exact number on notice. I can come back and tell you, but we have an internal process or program of training and testing to allow people to become a QA officer.

Senator O'NEILL: Do they actually have access to the draft document? Is it a locked PDF that they just make comments on, or can they actually get in and change text?

Mr Morphy: I would have to take that on notice. I am not exactly aware of what IT access they may have. I will have to take that on notice.

Senator O'NEILL: You talked about review and refinement, and language that might have been tightened. What do you mean by that?

Mr Morphy: As I said, from the point of view of the doctor, what they want to produce in terms of quality is a document that is clear and concise. So they will look at their work, ultimately, and ask the question: is this clear and concise? Could it be improved? The doctor is always free to refine their final report before they sign it and issue it. That is in their vested interest, and it is in our vested interest to make sure that it is as robust and high-quality as possible. That is what we support them in doing.

Senator O'NEILL: I might come back with a couple of questions, but I will hand over now to Senator Ketter.

Senator KETTER: Pursuing that line of questioning, have there been occasions when it has been found that the final report on your system is different to the report that the doctor believes they signed off on?

Mr Morphy: No. That is a very tightly controlled process. The final signed report is taken by us and stored indefinitely and provided to the requesting party. We are always 100 per cent confident that we have the final signed report.

Senator KETTER: Has there any been any accusation that that is the case?

Mr Morphy: No, not that we do not have the final signed report. No, not that I am aware of.

Senator KETTER: Going back to the business model, explain to us a bit more about how you generate revenue through your business. This is not having a go at your particular business. This is just the sector of the industry that you are in. How does it work?

Mr Morphy: In terms of IMEs?

Senator KETTER: Yes, but particularly as to how your organisation does it.

Mr Morphy: We, as an organisation that facilitates quality IMEs, look to have a panel of doctors that cover all jurisdictions, all geographies and all specialties.

Senator KETTER: Could they be GPs as well?

Mr Morphy: No, GPs do not typically provide IMEs. We do not have any on our panel. They are all expert medical practitioners. Any party that wishes to secure an IME is able to come to us with confidence that we will be able to facilitate for them an IME that suits their particular unique requirements, whether that is by jurisdiction or a particular insurance scheme. We have appropriately credentialled people available. Then there is that process of being able to facilitate an examination. For example, we have a network of clinics—facilities across Australia—so people can come to a facility to facilitate the IME and be examined. We have all the infrastructure to support that happening.

Senator KETTER: Where do you generate your revenue as a business?

Mr Morphy: One of the key parts is from IMEs. Organisations ask us for an IME. We facilitate it. We charge them, bill them. One of our key costs is obviously to pay the doctor for providing it. We look, obviously, to cover our own costs and, yes, we look to make a profit out of doing that.

Senator KETTER: So your revenue only comes from the medical profession?

Mr Morphy: It comes from the main users. I think that is why I outlined for you in section 3 of my submission a long list of the different industries that are users of IMEs. We approach all of those industries to make them aware of the fact that we are a quality provider and can typically service them. So we get IME requests from them.

Senator KETTER: And do you receive a fee from them?

Mr Morphy: Yes, they pay for the IMEs. When they request an IME and we provide it, they pay us. That is the standard model. It is as simple as that.

Senator KETTER: By going through your organisation to get the IME, would they be paying a premium on what an ordinary person would get?

Mr Morphy: Again, it is very hard to know, but they are free to approach doctors. How much a doctor would charge them individually, I cannot comment on. Potentially. But I cannot comment on that. I am just speculating.

Senator KETTER: Looking at your MedHealth customers, can you tell us where the majority of your work comes from? Is it evenly distributed across that list? Where would the big revenue come from?

Mr Morphy: Probably the first seven would be where the predominant amount of our business would come from, and quite an even spread.

Senator KETTER: The first seven.

Mr Morphy: Yes, all the points 1 through 7.

Senator KETTER: Would it be fair to say that the vast majority of your business would come from organisations that have a vested interest in either the minimisation of the insurance claim or the declining of the claim?

Mr Morphy: No.

Senator KETTER: You do not agree with that.

Mr Morphy: We provide IMEs for plaintiff law firms. So no, I do not think so.

Senator KETTER: You mentioned the first seven were the most significant. I presume the legal market is the one you are referring to there.

Mr Morphy: What you are really asking me to do is to speculate on the decision-making that happens once the IME is provided.

Senator KETTER: No, I am not asking you to speculate. There is a vested financial interest in the outcome of the claim.

Mr Morphy: There is always a financial consequence of the claim being determined. Again, it is not a matter of convenience that I sit here and say, 'We have nothing to do with that.' It is a very deliberate construct. We provide quality IMEs. That is an input into decision-making by either a plaintiff lawyer, an insurer or a court. We quite deliberately stay out of that. We do not have the experience or expertise, so we stay away.

Senator KETTER: I am not accusing you of getting involved. I am just asking the question. The top 7 of the clients that compose the vast majority of your business are entities that have a vested financial interest in the minimisation of a claim or the declining of a claim.

Mr Morphy: You would need to ask them about their vested interest. I would not want to sit here and speculate. That is not my industry and not my job.

Senator KETTER: Would you agree that there is potential for conflicted advice from doctors? If they are on your panel, and they become financially dependent on that work coming through to them, would you agree that there is a potential for conflicted advice?

Mr Morphy: No, I do not. I think the key possibility for a doctor being negatively impacted is not providing a quality IME. That is what they are being asked to do. And that ultimately is to give the right answer—an answer that stands up to scrutiny. If someone does not want that, do not ask. But if they have a reputation for not doing that then they have a problem. I do not see how they would survive in the industry.

Senator KETTER: We seem to accept that there is a need to look at conflicted remuneration when it comes to financial advisers and other people who provide a service. Why do you think that doctors are not in the same situation?

Mr Morphy: Because fundamentally, as an independent expert witness, what they are being asked to do is provide a robust defensible position in relation to particular questions. That is the core of their reason for existing and being there. That is what they need to protect.

Senator KETTER: How many individual claimants would use your company's services for an IME?

Mr Morphy: That is commercially sensitive. Basically, you are asking how big our business is, and that is commercially sensitive.

Senator KETTER: No, I am just asking about what I would think would be a fairly small proportion of your business. You listed it there: treating medical practitioners and their patients. It is on your list under section 3.

Mr Morphy: Which one?

Senator KETTER: In your letter, under section 3, the last dot point is 'treating medical practitioners and their patients'.

Mr Morphy: That is a relatively small part of the business. It reflects some specific schemes—one that was actually developed in South Australia around return to work. The concept there, if you would like me to explain it, was that the workers compensation scheme in South Australia funded a service whereby a treating general practitioner could refer their patient for a funded independent report, which was prepared by one of our doctors or any doctor doing this, and then given back to the GP to support them in managing and supporting their patient.

Senator KETTER: But otherwise, unless there was some sort of government backing for that--

Mr Morphy: It is part of the scheme, yes. Theoretically it could be done, but that would just fall under the normal health system.

Senator KETTER: This comes back to my point that the vast majority of the clients that use your service are entities that have a vested financial interest in a minimised outcome from an insurance claim.

Mr Morphy: Again, you do need to ask the users. It is not for me to speculate about that.

Senator KETTER: In part 5.4(a) of your letter talking about the recruitment you say you maintain a diverse panel of specialists. Do you have difficulty in recruiting specialists to your panel?

Mr Morphy: It is an ongoing process. Some areas are harder to recruit for than others. Some geographies are harder to recruit for, some are easier. It is an ongoing process, but, as I said, I think one of the things we are proud of is we have a very large, diverse, high-quality panel, but it is an ongoing process of recruitment.

Senator KETTER: It could be quite a lucrative engagement for a medical specialist to be involved with your organisation?

Mr Morphy: They get remunerated for it. How it compares to, for example, operating, I think you would need to ask doctors as to what their views about that are.

Senator KETTER: In terms of the documentation, in the second final page of your submission under the heading of 'Coordination of information', you talk about you say that you retain control over the supplied medical information and the final report in a secure environment.

Mr Morphy: Yes.

Senator KETTER: That final report is very important to claimants.

Mr Morphy: Critically important, yes.

Senator KETTER: It could be a deciding factor in what happens to somebody. What is the potential there for—and I am not suggesting that you would be necessarily aware of this—tampering or altering that medical information which is entirely within your control there?

Mr Morphy: I cannot guarantee none, but I would say absolutely minimal opportunity, and certainly the intent from our perspective under an ISO standard is to be able to secure information to ensure that does not happen.

Senator KETTER: Who retains that information? Is it any particular person who has a responsibility for a particular claim?

Mr Morphy: We have a process that is under the broad governance of the management team and the executive. Obviously I am ultimately responsible for that. It is a detailed process and program for securing information. I can take it on notice as to more detail if that is what you want about how exactly that is done.

Senator KETTER: Yes. Who has access to those files? Who retains them?

Mr Morphy: I need to take that on notice.

Senator KETTER: That is an extremely important part.

Mr Morphy: Absolutely. We take information security incredibly seriously and are currently investing heavily in that to keep improving what we are doing. I would hope that, if we are here in a year's time talking about this, if you ask, it would be a different world again in terms of where we have got to in that ongoing investment and improvement in information security.

Senator KETTER: Are any of the claims officers that have access to this information subject to remuneration packages linked to the outcomes of particular insurance claims?

Mr Morphy: Absolutely not.

Senator KETTER: Can you describe—

Mr Morphy: Sorry, any of our staff?

Senator KETTER: Yes.

Mr Morphy: No, absolutely not. Firstly, we do no have visibility in terms of what a claims outcome is. As I said, and I keep emphasising this point, it is not just an answer of convenience, it is an answer of deliberate construct, that once the IME is handed over we do not want and we do not have any involvement in decision making. We recognise it is an important, potentially very important, input into decision making, but that is it. It is not the only thing and it may or may not be ultimately relevant. Very deliberately we do not get involved in that. We are not skilled, experienced or knowledgeable to do that. It is not our job. If we did, we would be setting up a conflict. Very deliberately we hand over the report and we have no involvement in decision making. Specifically to your question of do we link remuneration to it, absolutely not because we could not do it anyway because we

do not know what happens with it. Again, that is a very specific and deliberate construct. It is not just a convenience. We think carefully about that.

CHAIR: Senator Ketter, we are running out of time. We have a question from Senator Williams.

Senator WILLIAMS: Mr Morphy, in a question from Senator O'Neill earlier on did you say that some of these doctors, these specialists, employed might do a job for four or five hours for a $500 or $600 payment? Were they the figures you used?

Mr Morphy: There is a range of time involved and there is a range of figures that they might be remunerated. It is a very, very fluid situation. All I can say is that there is a range of how long it may take and what they may be paid.

Senator WILLIAMS: If there were a case where they would do four or five hours work for $500 or $600, if I were a cardiologist or orthopaedic surgeon or ophthalmologist or whatever, I would not get out of bed for $100 an hour. The question I am going to follow up with is: are many of these specialists in the field that you use elderly or looking towards retirement?

Mr Morphy: Some of the specialists are older people, yes. We are not ageist though. As long as they have met all, and hold all, the appropriate qualifications and registrations, and we believe they are producing quality IMEs, we allow them to work. We are not in the process of dismissing people based purely on their age.

Senator WILLIAMS: I thought you might have to have some sort of case of heading towards retirement or semi-retirement because young, 30-year-old or 40-year-old specialists in the modern day field would be on thousands of dollars an hour, not hundreds.

Mr Morphy: As I say, the circumstances for each doctor participating obviously vary. You should interview some doctors who are doing this and asking them that question.

Senator WILLIAMS: I would like to interview some doctors. They will not come along.

Mr Morphy: I will leave that to you to resolve.

CHAIR: We will just go now to Senator O'Neill.

Senator O'NEILL: Are the reports of the IME provided to the claimant and the claimant's doctor?

Mr Morphy: Are they provided? It does depend on the circumstances. Once we hand it over to the referring party the obligations that they have under their own arrangements as to who gets it is a question that they resolve. Obviously if we were to receive a court order we would comply with it. It is a very fluid situation and very different in different jurisdictions as to how that is resolved. So there is not just an answer to that question.

Senator O'NEILL: I do not know that you will be able to answer this. What weight does a report from an IME have in comparison to a report from a treating doctor?

Mr Morphy: Sorry, what was that?

Senator O'NEILL: What weight, relative weight.

Mr Morphy: It would completely depend. Put it in front of a court and obviously the whole concept of an independent, expert report is exactly that. I think I mentioned this in the submission. They are independent, they are not treating, they do not have a relationship to any party beyond being paid for their report. They do not have social relationships or a personal relationship with any of the parties. That is what gives them the ability to stand up in court and say, 'I am independent. I am an expert and here is my opinion as to what the situation is.'

Senator O'NEILL: The assumption is that all of these independent medical examiners are putting forward reports for courts. Is it the case that they always go to court or is it just that they are just returned to the insurance company who then will make a determination one way or the other?

Mr Morphy: Certainly they do not always go to court.

Senator O'NEILL: In terms of the scrutiny test that you have been alluding to in your evidence, how many occasions are there? I am assuming quite a number of occasions in which there is no scrutiny applied other than the insurer seeing the IME's report.

Mr Morphy: That is correct. What I can tell you is that every doctor—and this is one thing that I know because you can ask any doctor this question—in the front of, the back of and the middle of their mind is that they may be standing up, which is a distinct possibility, defending what they have written in an open court in front of their peers.

Senator O'NEILL: Or they may not.

Mr Morphy: Or they may not, but that is the possibility.

Senator O'NEILL: In what percentage of cases is the IME's report advanced to court rather than just directly to the insurer?

Mr Morphy: As a rough estimate it would be approximately five per cent of the time, I would think.

Senator O'NEILL: Five per cent go to court?

Mr Morphy: Yes.

Senator O'NEILL: And 95 per cent never see the light of day of that sort of scrutiny?

Mr Morphy: They do not end up in a court. Again, what happens to them after we hand them over to either a plaintiff lawyer who is requesting them or to an insurance company is up to them. We have done our job in providing a quality IME. They then deal with that.

Senator O'NEILL: Five per cent—that is very interesting. Are there any industry standards for independent medical examiners and who is responsible for ensuring compliance with those standards?

Mr Morphy: There is no industry regulator in relation to IMEs. However, as I have said, doctors preparing IMEs are subject, as expert evidence, to a series of codes of conduct for experts and their own professional regulation. Those are the standards. We have self-imposed standards in relation to being ISO certified.

Senator O'NEILL: Does the AMA have a role to play here in guidance for independent medical examiners?

Mr Morphy: No. To the extent that these people are doctors and they maintain professional registration, they are subject to the rules of their governing bodies.

Senator O'NEILL: But there is no particular oversight body?

Mr Morphy: There is not a specific industry body regulating IMEs.

Senator O'NEILL: Does the Australian Health Practitioner Regulation Agency have any oversighting of independent medical examiners?

Mr Morphy: To the extent that they have to remain registered—that they must meet their registration requirements.

Senator O'NEILL: What avenues are available for independent medical examiners to report any wrongdoing by insurers?

Mr Morphy: I think this question was asked earlier. There are multiple avenues for them to raise concerns.

Senator O'NEILL: I will look at the *Hansard* for that. I have clearly missed it. With regard to the question that I asked you earlier, I believe it is in the public interest to have an understanding of the nature of the remuneration that somebody who works exclusively as an IME might attain if they dedicated their time to that. I ask you again if you would put a quantum on the record.

Mr Morphy: Can please I take that on notice?

Senator O'NEILL: If you would not mind doing that and providing it to us in writing, the secretariat will be in touch.

Mr Morphy: Okay.

CHAIR: We are out of time. Thank you for attending today. Any answers to questions taken on notice should be provided by 9 June 2017. The secretariat will write to you with the list of questions that have been given to you on notice from the *Hansard* record. Thank you for attending the hearing and for your evidence today. The committee will now suspend for a short break.

Proceedings suspended from 14:27 to 14:34

LACAZE, Dr Paul, Head, Public Health Genomics, Department of Epidemiology and Preventative Medicine, School of Public Health and Preventative Medicine, Monash University; and Founding Member, Australian Genetic Non-Discrimination Working Group

TILLER, Ms Jane, Legal and Social Adviser, Public Health Genomics, Department of Epidemiology and Preventative Medicine, School of Public Health and Preventative Medicine, Monash University; and Member, Australian Genetic Non-Discrimination Working Group

OTLOWSKI, Professor Margaret, Law Dean, University of Tasmania; and Chair, Australian Genetic Non-Discrimination Working Group

CHAIR: The committee welcomes witnesses from the Australian Genetic Non-Discrimination Working Group. Their submission is No. 60. I remind committee members and witnesses that, while this is a public hearing, care should be taken to protect the privacy of individuals and that arguments should be made without naming individuals. I invite you to make an opening statement.

Prof. Otlowski: We did send through a one-pager. I trust members of the committee have that. If not, I can make that available.

CHAIR: We might request a copy of your opening statement as well—as soon as you have finished reading it.

Prof. Otlowski: That is what I am saying. I have copies of my opening statement available here. We are grateful to have the opportunity to appear before this inquiry. As background, it might be helpful to let the committee know a little bit about my own expertise in this area. I have been involved in academic research for some years in the area of genetic discrimination. I led research under an Australian Research Council grant, a Discovery Project grant, that examined in an empirical way the phenomenon of genetic discrimination in Australia. I was also a consultant to the Australia Law Reform Commission inquiry looking at the use of genetic information, including in insurance. Then for six years I was a member of an NHMRC principal committee, the Human Genetics Advisory Committee. I mention those things to provide background and to explain that I do have some experience in this area on which I can draw.

The summary we have prepared, and which I want to briefly touch on before we open for questions, represents a crystallisation of our original submission. It represents the view of a majority of our working group. We have two key recommendations. The first is that the Australian government should enact legislation to regulate the use of genetic information. Ideally this could be through flexible legislative instruments. The second recommendation is that, until such legislation is in place, the Australian government should enact a ban or moratorium on the use of genetic data by life insurers. I want to make it clear that when we talk about genetic test information we are talking about predictive genetic test information regarding individuals who are not presently affected by a condition.

I will run very briefly through the points that underpin our submission and that lie behind these recommendations. The first relates to the relevance of genomic test information in health care and how this is increasing but clearly has privacy implications, as well as raising concerns about genetic discrimination. These concerns are growing as the use of genetic test information is increasing. This in turn puts pressure on people when it comes to financial matters—insurance is the obvious example. The 2003 report of the Australian Law Reform Commission inquiry recommended allowing the continued use of genetic test information by insurers, but that recommendation was predicated on balancing that with key consumer safeguards. It is regrettable that key aspects of those safeguards were not implemented. The area has not really been addressed by the Australian government since that time. In the meantime, there has been significant investment at the state level—in the tens of millions of dollars—in the implementation of genomics health care, yet the insurance implications and the effect on public uptake have not really been addressed.

Internationally, many countries, for a range of legal, ethical and social reasons, have enacted moratoria or legislation or have in some other way restricted the use of genetic test information by insurance companies. In contrast, in Australia we have a self-regulated industry subject only to the exemption that exists in the Disability Discrimination Act which gives the power to discriminate in insurance underwriting—but that is not specific to genetic test information. Reflecting, as we have been doing, on the developments over the last 10 or more years, it is our submission that this self-regulation approach is not in the public interest. We have identified a number of particular concerns. These include fears about genetic discrimination being a deterrent to people undertaking clinically useful genetic testing, as well as discouraging people from participating in research. We also question the adequacy of data for robust decision making.

Despite claims by insurance companies that adverse selection would result if they were not given access to genetic test information, we note that there is very little or no evidence put forward to support such claims. In fact you only need to look at the European experience—and now more recently that of Canada, where legislation has been introduced to restrict such access. Expert evidence given before that decision was made suggested that it would not have a significant impact on insurers or the operation of an efficient insurance market.

In our original submission we have made a number of cascading recommendations. In the interests of transparency, I should say that these were achieved through a consensus approach within our working group. We have some 12 members. There were some differences of view about the optimal method for regulating this area, but I want to stress that everyone in the group believes the status quo is unsatisfactory and that something needs to be done. In the course of appearing before this committee and in engaging with the working group, we have sought to crystallise our key recommendations, and we came to the strong conclusion that direct government action is needed. We believe a legislative ban or moratorium is required. This reflects the majority view of the working group.

Senator WILLIAMS: Thank you for your evidence and your presence here today. I was on an inquiry with former Senator Heffernan that looked at the relationship of a particular gene to breast cancer.

Prof. Otlowski: Yes, BRCA1.

Senator WILLIAMS: That is the one. I could not think of it. What you are saying is that you do not want to see insurance companies go into gene testing because a young lady might have the BRCA1 gene, marking her as likely to develop breast cancer. As a result, insurers would either not want to give her life insurance—especially trauma insurance—or they would want to charge her an exorbitant premium. This is the guts of your argument? Am I on the right track?

Prof. Otlowski: Yes, it is. The essence of our argument is that we do not think it is appropriate for life insurers to be given access to genetic test information for a whole range of reasons. In part it is the inadequacy—

Senator WILLIAMS: First of all, it would be very unfair, would it not?

Prof. Otlowski: We think so.

Senator WILLIAMS: Because you cannot help the genetic make-up you are born with.

Prof. Otlowski: Indeed.

Senator WILLIAMS: If you are highly likely to develop, for example, breast cancer, you could be paying enormous premiums to cover your trauma, your income replacement and even your life policy—because of something you have no control over.

Prof. Otlowski: That has certainly been one of the factors behind some of the European countries adopting an outright ban.

Senator WILLIAMS: That has happened in European countries, you say?

Prof. Otlowski: Yes.

Senator WILLIAMS: They have legislated, have they?

Prof. Otlowski: Yes, they have—quite a number. There have been dozens of European—

Senator WILLIAMS: Can you name some?

Prof. Otlowski: Yes. Belgium, Austria, Denmark, Germany, Norway and Portugal are among them. There is a whole list. Some have legislation while some have had a moratorium—and some have had a moratorium and then legislation. It is an extensive list. It is linked to their endorsement of the European bioethics convention, which has certain protections against discrimination.

Senator WILLIAMS: I remember that inquiry very well because it was very traumatic for some of our witnesses. One young lady of about 35 had her breast removed purely because she was highly likely to develop breast cancer. It was a terrible inquiry as far as the stress levels were concerned. The last thing we want is to have genetic testing to make life even more difficult for those people who are unfortunate enough to have that in their genetic make-up.

Prof. Otlowski: There is that general issue of fairness and people having no control, but, from the research I referred to earlier, there are also examples where people who have had that mutation identified have taken steps. There was one particular instance where we could conclude, in a verified sense, that it was unjustified discrimination. A woman had prophylactic surgery, a double mastectomy, to remove her breasts, and then the insurer did not take that into account in the way they should have. Our independent assessment of this case indicated that she should have been treated as having lower risk than the average Australian woman—so there

was clear discrimination in the way her application was treated. That casts doubt on the capacity of the insurance industry to fairly deal with such matters.

Senator WILLIAMS: Poor girl! You said in winding up your opening statement something about the status quo. I missed it. What did you say about the status quo?

Prof. Otlowski: The nature of the self-regulation—

Senator WILLIAMS: This is of the insurance companies?

Prof. Otlowski: Yes. More than a decade ago, recommendations were made assuming that certain things would be done, that there would be some way of working out which genetic information could be safely used for underwriting. Initially this was designated to be the activity of a new committee, which ended up being the Human Genetics Advisory Committee. But, if you look carefully at the government's response, you see that it said of the list of recommendations that they were a matter for the industry—at the time it was IFSA and the Insurance Council of Australia. During my six years on the Human Genetics Advisory Committee, it was constantly a matter on the committee's agenda. We were very limited in our ability to do anything, because action required the cooperation of the industry—and nothing ever eventuated. That experience tells me that leaving it to industry to make the rules, to regulate its own use of genetic test information, has not worked. Something stronger is required.

Senator WILLIAMS: I ask the secretariat to take particular note of what we have just discussed. I think it is a very important issue for the committee to address in its recommendations.

CHAIR: Are there many people in Australia who have had genomic mapping done?

Prof. Otlowski: If by 'genomic mapping' you are referring to whole genome sequencing, that would be rare—because that is quite a new development. Paul would be able to speak to that. But for decades people have been having some form of genetic testing. That has become progressively more available as the relevant gene has been identified for more and more conditions.

Dr Lacaze: Looking at every gene in someone's genome is quite a new concept. It has only been made possible recently because of technological advances.

Senator WILLIAMS: It was not around 200 years ago when life insurance companies were established, was it?

Dr Lacaze: Certainly not. It is still mostly done in the research realm. For example, the research study that I work on is part of a very large clinical trial and cohort study of about 15,000 Australians who have provided blood samples for research purposes—purely altruistically. Part of what we are doing is genetic research to look at their whole genomes to try to work out the genetic contribution to some of the things that happen in their life, whether they are health or disease related. We are doing some whole genome sequencing. One of the things we really want to find out is how frequently we see genetic changes in these healthy individuals that are being used to predict risk—or assume risk—in other settings. What we are finding is that we see them commonly in healthy people—more commonly than we originally thought we would. Part of the reason for that is that the only data we have to date on genetic testing has been from affected families and patients who have been tested in a clinical setting. That is one of the fundamental things. There is a lot more research that is needed to understand the complexity of genetics in relation to future disease risk.

One of the other complications is that, if you are doing research and you find something that might be informative for an individual and their health and you feel ethically obliged to disclose that result from your research to them, they would then be obliged to tell their insurer that result, and then the insurer could make decisions based on that result which we do not have full transparency into. This is a real concern of mine with the study that I am working on in that, if we find things and disclose them to our participants who have provided samples altruistically and then have negative concerns or issues with their insurance, that is a big issue. That is where it all started for me. The further I explored this issue, the more I saw it was a real problem in our country under the current regulation—so much so that, when people and families go to the clinic for genetic testing for medical reasons, they are often informed that they need to go and revise their insurance before they take the test. For me, that really does not seem like a good situation at all for anyone. Further to that, many people then decide not to take the test that they need that might be critical for their health and management of their risk, because they have a perceived or real insurance concern. For me, that is another real problem.

CHAIR: What about at the other end of the scale with someone having their testing done and saying, 'Well, I'm never going to get sick, so I'm not going to take any insurance out' and then having a mutation later in life and getting caught because of that? Is that a crazy theory?

Ms Tiller: At the moment, it is easy to look at what can happen later versus what can happen now. I think that with genetic testing one of the risks is that people think that you can look at your genetics and find out everything about someone—you can tell them when they are going to die—and that is just not a reality now. At the moment, the testing that we are concerned about people not taking up is for things like BRCA, Lynch syndrome, which leads to colorectal cancers. These are things where there are pretty well understood risks and they know that something can be in the family, and people decide not to find out whether they have a particular gene change that could tell them whether they need extra screening or they can access extra surveillance. They choose not to take up that testing, because they are concerned about what is going to happen with insurance and insurance discrimination. That is published data.

Dr Lacaze: We would like to think that an insurance concern should not be part of the medical decision-making process for a test that might be very important for them in mitigating their future risk.

Ms Tiller: Equally, based on what Paul said, people are not being involved in research, because they are concerned that genetic results will be returned to them and they will have to disclose them to insurers when there is no benefit to themselves but enormous benefit to health care, to technology, to genomic knowledge. This testing and this research is really necessary for us to advance forward, but, while there are insurance concerns and while people are aware that they might be discriminated against on the basis of this, they will walk away from research. That is a really critical thing for our genomic future.

Dr Lacaze: Especially at this point in time, when we are starting to understand how we could use this information to provide better health care. There is an implementation effort. Other countries have taken steps and been progressive to make this not an issue for them to advance their research, industry and clinical care. That is what I think we need to do.

CHAIR: What stage are we at on the research scale compared to other countries?

Dr Lacaze: We are very competitive.

CHAIR: Are we advanced, above average or—

Dr Lacaze: I would say definitely above average. I think we have fantastic biomedical science in Australia and still have an opportunity to resolve this issue before it becomes a major public concern. However, if we do not resolve the issue in a timely way, we may restrict our ability to remain competitive and progress in the way that we should.

Ms Tiller: And there are things like the 100,000 Genomes Project in the UK, where they are sequencing hundreds of thousands of people's genomes, but the UK has protection in place. They have a moratorium where genetic test results are not used other than in specific contexts above a certain policy limit and only for Huntington's Disease. So people in the UK, when they are enrolled into this enormous nationwide study, have no fear about how their information is going to be used by an insurer. The public buy-in and engagement with research is unfettered by that, whereas here, if the situation stays as it is now, we have real concerns that, if Australia wants to move into that kind of study, it just is not going to be able to have the same public engagement and buy-in. The public will rightly fear what is going to happen.

CHAIR: If you had the theoretical opportunity to put one recommendation in our report, what would it be? What would be your primary recommendation?

Prof. Otlowski: I think it is there on our one-pager: that there is a restriction on insurers' access to genetic test information, whether that is through a legislative ban or a moratorium, so the consumers, the patients and participants in research have no fear about the risk of genetic discrimination. This should not be left to the industry with self-regulation; it should be direct intervention by government to restrict access to genetic test information of a predictive kind as opposed to diagnostic to explain why someone is already sick.

Senator O'NEILL: In your recommendation you use the term 'restrict' the use of genetic information, but here you have written 'regulate'.

Prof. Otlowski: Ideally, we would want no genetic test information to be in the hands of insurers. You could say that is a form of regulation. Clearly, there are a number of ways that restrictions can be achieved. It can be through a total ban. It can be through a moratorium whereby there is an agreement that the concordant—

Senator O'NEILL: But, to be clear, the policy outcome is that there would be no access to genetic information that was predictive?

Prof. Otlowski: Yes, that would be the ideal situation.

Senator O'NEILL: I just wanted to be very clear about that.

CHAIR: What if the person who is applying for life insurance has to tick the box about access to medical records?

Prof. Otlowski: We would be concerned about that. We were here in the previous session, and that concerned us as it concerned you, Senator O'Neill.

Ms Tiller: That is one where we would call for a ban on the use of genetic information, but part of what we are asking for in regulation is that, for example, if they had access to a medical record through the checking of the box or another way, there is legislation that regulates how they can use it and that restricts their access to it specifically as genetic test information separate from the rest of the medical record.

Prof. Otlowski: So either they do not have access or they are not allowed to use it for insurance underwriting if it is sitting there on the record.

Senator O'NEILL: Yes. I am thinking that not having access would have a whole lot more appeal.

Prof. Otlowski: That would solve a lot of it.

Senator O'NEILL: It is difficult to think about how that might be managed given the different systems for e-health recordkeeping across the country as it is, the different usage structures and also just the pressure that is on doctors.

Dr Lacaze: There are electronic health records that do not contain any genetic information and then there are some internal hospital systems that can. So there have been some mechanisms set up to deal with sensitivity around genetic data. How that would scale across the whole country is not figured out yet to my knowledge.

Ms Tiller: If there were clear regulation about that, it would force medical systems to take that into account, whereas now it perhaps is not something that is well regulated. It is not on the agenda.

Prof. Otlowski: Certainly, to the extent that genetic information is recognised as sensitive information—for example, under privacy legislation—there is already that recognition that it is of a particularly heightened sensitivity because of the familial nature and the capacity for people to be harmed through discrimination and stigmatisation.

Senator O'NEILL: I think mental health has quite a degree of similarity, and that has been well prosecuted in the course of these inquiries.

Prof. Otlowski: Yes.

Senator O'NEILL: I have a number of questions and might go over some of the general introductions you have given, but I want to work my way through them because I think they are quite good ones. I also want to acknowledge that you have had a meeting with Mr Keogh. Is that correct?

Ms Tiller: Yes.

Senator O'NEILL: He is sad that he cannot be here, but I think there are some important pointers from him with what we need to get on the record. Could you briefly outline the basis for why using genetic information is not a good basis for underwriting decisions?

Prof. Otlowski: I can perhaps begin to answer that. It is the concern about the adequacy of current data—

Senator O'NEILL: And the quality of its—

Prof. Otlowski: and the robustness in an actuarial, statistical or other basis. Insurance exemption allows insurers to do things if they have quality data that can help justify their decisions. Our concern is that in this area, and particularly with whole genome sequencing and much expanded genetic testing, we just lack the comprehensive quality data that allows robust decision making. Rather than allowing decisions to be made on potentially inadequate data or leaving it to individual complainants to have to call on the insurer to demonstrate that their decision was justified, which is putting the onus somehow in the wrong direction, the safer thing is simply to say do not have access to it, and then these problems do not arise.

Senator O'NEILL: The robustness you explained a little, Dr Lacaze. What the sample size is right now and the nature of that sample means that there is a certain degree of robustness that has not been reached because we have not got to a point of scale where that can be more or less assured.

Dr Lacaze: Exactly right, and that is what the research community is working towards—understanding everything better. In general, genetics is not deterministic to begin with. That is something we need to get past. Our understanding of our genes might provide some insight into risk but at the moment that is extremely imperfect information, yet it might still be pertinent to some people and some families who could use that information to take steps for closer surveillance or prevention of disease. But it is not at the level where it could be used to discriminate or predict with certainty future outcomes, or be considered actuarial.

Senator O'NEILL: Yes, so it could be subject to actuarial capture because it has statistical appeal for them, but the validity of those statistics, you say, is still highly questionable because it is a burgeoning field.

Dr Lacaze: Yes.

Senator O'NEILL: Plus, then, the ethical questions in addition to that. Is there a basis for increased premium or risk based on genetic information and does the data back that up?

Prof. Otlowski: In the event that insurers were denied access—we would refer to the European experience and the UK experience, where there seems to have been no significant impact on the life insurance industries in those countries. The research that was undertaken before Canada recently enacted its reforms also indicated that they anticipated insignificant or minimal impact. You may go through looking at some modelling exercises, but I think the best example is to look at the experience of countries which have actually done this. It was actually interesting to note presenters to the inquiry earlier saying either they did not necessarily take a view one way or the other about whether they should be entitled to genetic test information or they did not think that their companies were using it. So perhaps for individual insurance companies it is not such a big issue. It was quite interesting to hear that evidence this morning.

Senator O'NEILL: I did clarify with you that you have reduced your original seven recommendations down to two. You clearly want no access for insurance companies to genetic information. Your second recommendation is that there should be a moratorium immediately—am I reading that correctly?

Ms Tiller: Yes.

Prof. Otlowski: To be clear, we are talking about genetic test information. We do understand that family history has been used for many years and we do not think it would be reasonable to say insurers cannot ask questions about family history. So it is the information through predictive genetic testing that we are identifying as the issue that needs to be restricted.

Senator O'NEILL: Genetic testing is really sort of family history on steroids—isn't it?

Dr Lacaze: Not necessarily.

Senator O'NEILL: Explain to me what is wrong about that analogy.

Dr Lacaze: There are always exceptions where people within an affected family might have a positive gene test result but never be affected by that disease. Likewise, there are people whom you might find that genetic change in who do not have any family history who may or may not get the disease. So this is part of what is required with far, far more research to understand completely.

Senator O'NEILL: Over the course of a lifetime, there might be somebody whose parents died at 50 but they live in a different context and live well into their eighties or nineties. We know that because time has provided us with that, but the appeal of science to lock it in can be something that would shorten that in an arbitrary and dangerous way.

Dr Lacaze: Also some people do not know their family history. Some people have a family of males. They may be carrying the BRCA gene, for example, but just by chance they have had sons instead of daughters, so you do not see it. So there are all those factors to consider.

Senator O'NEILL: You have made it clear that the industry policies and the industry self-regulation is inadequate. I will just leave it with what you have said there.

Dr Lacaze: I would like to put on the record that we need to give them credit for trying to make progress and taking the effort to understand this extremely complex issue. But we fundamentally do not believe that industry self-regulation is adequate for this issue.

Senator O'NEILL: And the background in which you make that statement is because the recommendations that are 10 years old have not yet been enacted or acted on.

Prof. Otlowski: Yes, that is the case. The *Essentially yours* recommendations about clearly identifying which tests could be used in a valid way were never implemented. Also recommendations about creating easy, accessible and cheap appeal avenues were also part of the *Essentially yours* report and left to the industry. These also have not been implemented, even though there was discussion and, at the time, goodwill expressed but it just never came to fruition. I know there were activities at the higher level within the peak bodies endeavouring to enable things to happen but without any government pressure it never came to fruition.

Ms Tiller: Fundamentally, the concept of allowing an industry that benefits from using certain information to regulate its use of that information is a flawed concept.

Senator O'NEILL: A slight conflict of interest?

Ms Tiller: Absolutely. The secretariat might be in touch with you to track down that document that you refer to.

Ms Tiller: We are happy to provide any documents that you are interested in.

Senator O'NEILL: Thank you. Should a moratorium or the legislation that you are recommending be subject to reviews?

Prof. Otlowski: Some of the commentators discuss that. It comes down to the need for future-proofing. Clearly this is a rapidly moving area and it is one of the reasons I talked about legislation, if it was going to exist, to be perhaps a flexible legislative instrument. It is very difficult to predict now where will be in five years time. And it is one of the reasons that within the working group there have been differences of view: do you lock it in with legislation?; do you try for a more flexible approach? A moratorium, if it could be organised—

Senator O'NEILL: Or legislation, if it was enacted, definitely subject to review because of the changing nature of the field?

Dr Lacaze: Yes.

Senator O'NEILL: Could you explain what an exemption for negative predictive test results would mean?

Ms Tiller: To counter family history?

Senator O'NEILL: Yes.

Ms Tiller: The concern that some people have is that—for example, if they have a number of family members who have died of breast cancer—when they go to an insurer they will look at their family history and rate them as a higher risk for dying of breast cancer, and if a family DNA change is found and they do not have that DNA change, their risk then becomes lower by having that test result, if that makes sense to you. So if you cannot use that negative test result—so 'I don't have the DNA change'—to tell the insurer, 'I'm at lower risk,' some people think you are in a worse position. For example, in the UK with the moratorium that they have agreed, insurers cannot use your test results to discriminate against you, but you can tell them about negative results to remove that family history impact on you.

Senator O'NEILL: Okay. so there is a sort of positive discriminatory capacity with a negative test result?

Ms Tiller: That is right—the ability to give your insurer that negative test result to say, 'This family history should not apply to me in rating my risk.'

Senator O'NEILL: I will leave that there and then if there is time I want to talk about another matter.

Senator KETTER: I recently visited the Kinghorn centre in Sydney and became aware that Australia is a leading player in genome sequencing. It was put to us that there are some great advantages to this technology, particularly in relation to quality of life for people down the track. This is all heading towards us, and certainly I appreciate that there should be appropriate safeguards in all of this, but I look at some of these developments in other countries. In South Africa, they are particularly looking at direct-to-customer genetic testing and identifying opportunities for people to have better health going forward, but then there is the potential for that information to have an adverse impact if the insurance company then has negative information. So there are a lot of things to think through.

Prof. Otlowski: Indeed, and at the moment, really, any genetic test result, it seems, would be required to be disclosed. It does not differentiate.

Dr Lacaze: It is a really important point. The test results you can get in a clinical genetic service in Australia have a huge amount of quality control around them and clinical oversight, and those direct-to-consumer tests are completely different. They are not in any way the same. They are making risk calculations based on a completely different method that is not really scientifically supported by the genetics community, by and large, and there is a lot of concern about releasing that risk information to people directly without any genetic counselling or medical oversight whatsoever. It is something that is emerging and needs to be considered carefully.

Ms Tiller: And insurers under the current regulations can require applicants to disclose any test information. So, if they have obtained the direct-to-consumer test from America, which has we do not know what kind of information or quality control, applicants will be required to disclose that if asked by an insurer under the current regulation.

Senator KETTER: Is guidance sought from expert geneticists when developing and reviewing actuarial modelling of the impact of predictable genetic disease?

Prof. Otlowski: I am sure that occurs in some cases. What I would question is whether this is done systematically. Certainly, in the research that was undertaken as part of the Australian Research Council funded

study, we came across a number of cases where there were adverse decisions made against individuals that would have denied them insurance or perhaps had exclusions or added to their premiums. It was only through the intervention of a clinical geneticist or someone equivalent that the insurer backed down and changed their position. So there would have been an unlawful decision if there had not been intervention through the proactivity of the patient with the expert that they brought in. No-one is suggesting bad faith on the part of insurers, but I do think there may be some educational gaps or not always the expertise at hand to make the required decisions—and we discussed the lack of robust and reliable data in the first place.

Senator KETTER: Are there any other jurisdictions which have legislation which you would suggest we emulate?

Prof. Otlowski: Yes, there are a number of jurisdictions. I can certainly send you a publication which refers to the countries which have introduced legislation and/or moratoria. It is an extensive list.

Senator KETTER: Would Germany be on that list?

Prof. Otlowski: Yes.

Senator KETTER: I note that, in the case of Germany, according to a law of genetic engineering and predictive testing, information is permitted to be requested and used where the sum assured exceeds the amount of 300,000 euros.

Prof. Otlowski: Yes, and this comes back to the question of whether you regulate and set some exceptions. Jane referred to the UK position, where there is, broadly speaking, a moratorium and no disclosure, but there is one exception. In the UK, it is for just one genetic test, namely Huntington's disease, and that is an unusual condition—autosomal dominant and full penetrance. Different countries will set moratoria and perhaps have some exceptions. That is a matter for, I guess, public debate as to what is fair and to protect the industry from risk of adverse selection. There may be some nuances between jurisdictions to avoid very, very large insurance applications being made where people know more about their condition that they do not have to disclose.

Senator KETTER: Should there be an independent oversight body to regulate how genetic data is used?

Prof. Otlowski: This is an interesting point. It was in between our recommendations. In a sense, we had something similar when we had the Human Genetics Advisory Committee. Firstly, it has been disbanded, but there was also a sense that it did not necessarily have the expertise to conduct this. I would also note that the UK had the Human Genetics Commission, which has also been disbanded. Whether we set up a separate and new oversight committee—reflecting on what has occurred with the setting up of these bodies that have been disbanded, it does not give me a lot of confidence that that is necessarily the correct approach.

CHAIR: How much work are they going to have at the moment?

Prof. Otlowski: Whether you can have a moratorium—in the UK they no longer have a standing committee, yet they have a moratorium that is reviewed every few years. I think there is an understanding by the insurance industry that if they are not voluntarily agreeing to this moratorium there will be legislation restricting their use. So there is this cooperative but in a way uneasy tension that comes to the right outcome. It is based on the fact that at the present time there are not that many people that have a genetic test result that is then going to influence insurance and adverse selection and so on. That has been in place now for more than a decade, since 2001 I think, so it is not as if it is just a short-term thing. It seems to be a good solution that has endured for some time.

Ms Tiller: Certainly one thing we would say is that there needs to be a government department that takes ownership of this issue. We have come to this hearing today because we think it is something that really needs to be strongly considered in the context of life insurance, but it needs to be strongly considered by the government at large. There does not seem to be a department that is tasked with taking care of and having oversight over this issue. There should be one, not necessarily a committee, but it should be in someone's portfolio to be responsible.

Prof. Otlowski: I would have thought, particularly considering the major investment that is now occurring in some jurisdictions, including Victoria, New South Wales and Queensland—$25 million each for the translation of human genomics into clinical care—it should become a health focus. Research is also health oriented. That would seem to be the optimal positioning of such oversight, from our perspective.

CHAIR: What about the ownership of the individuals' test results?

Dr Lacaze: That is a complicated issue that maybe we do not have time to discuss, but I think it is something that needs to be considered. Across the globe it is happening differently. Some governments are setting up safe mechanisms for storing genetic data of their population. Other people are providing their genetic information to commercial companies. At other times it is a hospital system that is storing the genetic information. At other times the individual themselves takes ownership of their own genetic information. So there are different models

emerging. I think something that is aligned with our strong health system that allows us to safeguard the security and privacy of genetic information for medical use is something that we need to figure out.

CHAIR: To go further, when a body discovers a new gene, say, does that remain in the ownership of the individual or can it be patented by the people who discovered it?

Dr Lacaze: That is another long conversation.

Senator O'NEILL: There has been a bit of lobbying around that over the years, hasn't there?

Senator KETTER: In relation to Financial Services Council standard no. 11 on genetic testing policy, which has just been updated in December last year, I know that your position is moratorium. Are there any specific comments you would like to make about the FSC standard no. 11?

Prof. Otlowski: Yes. I do want to acknowledge that the life insurance industry, particularly at the time of the Australian Law Reform Commission inquiry, had been very cooperative, endeavouring to be transparent. They had undertaken to collect genetic test information and publish the use by insurers, and that had occurred for some years, although more recently it had fallen away. We see efforts to create some protections, such as not to require individuals to have genetic testing undertaken so that there is no pressure to have testing. So we want to acknowledge the good things that have been done. Indeed, the research that I undertook would not have been possible unless the people from the peak bodies had made their data available to us. I want to give credit where credit is due, as Paul has done.

We have been looking very closely at the standard—the recent revisions. There are some concerns. In some cases, some of the new, revised wording seems to be watering down some of the protections. Where it had previously said 'must' it is now 'should' and language that is not quite as prescriptive—

Ms Tiller: 'May'.

Prof. Otlowski: Or 'may'. And there is a question about asking people whether they are considering taking a genetic test, which does not seem appropriate to us, because what is the relevance of that? You only have to disclose what you know in terms of an outcome of a test. So it seems difficult to understand the relevance of that.

Senator O'NEILL: Trying to get ahead of the day where they are told to correct their insurance before they—

Prof. Otlowski: Yes.

Ms Tiller: The recent iterations of the standard, in our view, are more concerning. We do not see that it is necessarily producing more protection for consumers; it is producing less and it is trying to tighten up access for the insurance companies and trying, like you say, to avoid people being able to have information that the insurers do not have.

Prof. Otlowski: To our mind there is nothing inappropriate for a person to take out insurance before they have a test, because at that time they know more than what they would have to disclose through family history. So it just seems irrelevant.

Senator O'NEILL: But, if you have to answer the question, 'Are you considering taking the test?' then that changes that—

Prof. Otlowski: But then what would an insurer do? Would they say, 'So we are not going to insure you,' or 'You will await the outcome of that test'?

Senator O'NEILL: Exactly. That is what I reckon they will do.

Ms Tiller: That is what they want to do.

Prof. Otlowski: But I do not think that is a justifiable decision for an insurer to take. If you look at the insurance exception and so on—

Dr Lacaze: One of the issues is that we do not actually know what the insurer is going to do when someone says yes to that question or when they provide a research finding, like they are obliged to. Or if there was no mention of direct consumer test results in the policy, what is an insurance company going to do with that information? There is a lot of uncertainty about what happens after the event.

Ms Tiller: Again, this comes back to it being a self-regulated industry. So they have revised the standard and it has come out and now that is how they are regulating themselves. And there is no oversight of whether that adequately protects consumers. There is no question of whether it is appropriate to ask consumers whether they are considering genetic testing. No government body looks at that. It just comes out and that is now the policy that they abide by, and if that does not protect consumers there is not much consumers can do about that.

Dr Lacaze: And they have had some interaction with some members of the genetics community. They will say that they have had input from the genetics community—

Senator O'NEILL: The sector is being consulted.

Dr Lacaze: Yes. We do not believe that is representative of the genetics community.

Ms Tiller: And there has been consultation, but—

Dr Lacaze: And it is completely at odds with the official Human Genetics Society of Australia statement, which is public, which is more in line with what we are saying about the need for a moratorium and the restricted use.

Ms Tiller: The Human Genetics Society of Australia's position statement on this issue calls for legislative reform and a moratorium on the use of genetic information. We can provide you with a copy of that document as well.

Senator KETTER: Have you detected any lack of willingness on the part of the public to involve themselves in research because of the potential insurance implications?

Ms Tiller: Yes.

Prof. Otlowski: It is now well documented. I know Paul could speak from experience on this. There has been published research, documented in various contexts, and it is a real fear.

Dr Lacaze: There have been a number of studies done, but especially in one of them, when people were asked the reason for them not participating in the research after the informed consent process, for over 50 per cent of all the people that said no, insurance concerns were the reason why.

Senator O'NEILL: Can you provide us with the research and the publications that you referred to?

Dr Lacaze: Yes.

Ms Tiller: We have also had researchers that we work with tell us that, when they have sought human research ethics committee approvals for research, the committees have started to require that they send people off for independent legal advice about insurance applications before they will be enrolled in the study.

Senator O'NEILL: That is going to slow down your research quite a bit.

Ms Tiller: It slows down research, but the cost—who can afford to go and get independent legal advice before they enrol in a study that is purely altruistic?

Dr Lacaze: And the reality is, the more informed someone is, the more likely they are to not participate in research, which is a big problem.

Ms Tiller: The ethics committees are trying to protect people. No offence to them, but it does mean that research is not viable and people will not agree to participate, because of these issues.

Senator O'NEILL: Following on from that, could I invite you to provide us with information—even if you think of it after this afternoon—that you think would inform our deliberations going forward. Could you explain to me what microarrays are?

Dr Lacaze: There are various different technologies that can be used to measure someone's genes. They range from single gene tests, or a single thing that you are looking for—that one thing, and that is all you want to know. Then there are more global methods, like DNA sequencing, where you can actually read every letter in that gene or in hundreds of genes or in the whole genome, and see how those letters change. A microarray is something in between, where there is a chip that has special sequences of DNA bound onto it. You can then take a sample of a patient's DNA and kind of bind it to this glass slide. It will give you a corresponding readout of features of that individual's DNA based on what is measured on that chip. It has been around for longer than DNA sequencing, but it is a more global way of measuring many different types of possible genetic changes at the same time in a single test.

Ms Tiller: It will not tell you every single change that might be in your DNA code, but there are a number of known sites where there are changes, and it can test known sites or it can give you an idea of whether there are known changes.

Dr Lacaze: In the diagnostic world it is used for diagnosing childhood and developmental issues, commonly as a diagnostic tool.

Ms Tiller: We were here for your earlier session. Commonly, if a child comes into a genetics clinic and has a number of features that look like they might be genetic, there might be an underlying syndrome. Either a number of genetic tests have been done and the cause has not been found or the clinicians are not sure as to what it might be. Routinely, a microarray and another test—called a fragile X test, which looks for fragile X syndrome—will be done as a first port of call to see globally whether there is anything that we should be looking at, any deletions or

additions of DNA that might be explaining this cause. So it is common for a child with an undiagnosed potential genetic disease in a clinic to have a microarray ordered as one of the early tests.

Senator O'NEILL: My understanding is that it is becoming a standard test in that sector.

Ms Tiller: Very common, yes.

Senator O'NEILL: That data is available, and it is being done predominantly in children.

Ms Tiller: It is also done in adults, but certainly it is a common thing in children for it to be done if there is a suspected underlying genetic disease.

Senator O'NEILL: Are you aware of insurance being refused on the basis that a microarray result is known and children cannot get insurance?

Dr Lacaze: It would be classified—as any other type of genetic information would under the current regulations—as something that has to be disclosed to insurers that they are able to act upon. So in a way it is no different from another type of genetic information from a test. It is just a method that is used. Sometimes the microarray test can pick up things that you were not expecting or that are not clear-cut, so findings are uncertain or need interpretation from experts.

Ms Tiller: The answer is: we are not aware of any instances. We do not know. Overwhelmingly, the answer is: we do not know what insurers are doing and we do not know what happens.

Senator O'NEILL: I have been familiarised with correspondence that indicates that that is the case.

Ms Tiller: Certainly I would not find it shocking, having seen what has happened in a number of other scenarios and a number of other cases with other types of DNA testing. It would not shock me to know that that is happening. But I certainly do not know of any individual cases myself.

Senator O'NEILL: I note that we have the CRCs, which were celebrated here earlier this week, and I know that there are plans by the Autism CRC to sequence over 1,000 autistic children. There is considerable concern about allowing that to happen in the current context. Do you believe that the recommendations you have put forward would provide a sufficient protection for those families—who are already facing quite a degree of challenge—to encourage them to participate in that research project?

Dr Lacaze: I believe that they would, yes. Autism is one of the many cases where there is not actually a way of diagnosing the disease clearly based on just a genetic test, and there is far more research required to understand the genetics of autism. That is probably what that research project is involved in trying to do. In order for that to happen, people need to participate in that research without being concerned about what the implications might be, beyond all the other issues that they are already having to deal with. A lot of these types of projects now are bridging research and clinical care, as we gain knowledge about what all of this information means. And so it is even more of a reason why safeguards are needed—so that transition, from research to actually being able to help people, can be made.

Senator O'NEILL: So that is prioritised over insurance.

Ms Tiller: Yes.

Dr Lacaze: Yes.

Ms Tiller: And if there was a complete ban on the use of this genetic testing information by insurers, in obtaining consent you would not even have to discuss insurance with people. You would not have to say to them, 'insurance is not a concern,' because it would not have to come up.

Dr Lacaze: Wouldn't it be wonderful if we could say to those families that we are doing all that we can to help them, and that they do not have to worry about insurance implications?

CHAIR: That would be great. To conclude: if you have been asked questions taken on notice, the answers should be provided to the secretariat by 9 June 2017. Once again, thank you for attending the hearing and for your evidence today—and thanks for travelling here. I know you have come a long way.

Ms Tiller: Thank you for having us.

LONGSTAFF, Dr Simon AO, Executive Director, The Ethics Centre

[15:32]

Evidence from Dr Longstaff was taken by teleconference—

CHAIR: The committee welcomes Dr Simon Longstaff from The Ethics Centre. I remind you that while this is a public hearing, care should be taken to protect the privacy of individuals, and that arguments should be made without naming individuals. I now invite you to make a short opening statement, and at the conclusion of your remarks, I will invite members of the committee to put questions to you.

Dr Longstaff: Thank you, I have a couple of brief comments. Firstly, I would like to apologise for not being there in person. This is the second occasion on which I have had to join the committee by telephone, and I would have preferred to be there face to face. Second, just to advise that at four o'clock I have to leave to catch a flight—I am calling from an airport lounge.

CHAIR: Okay, we will take that on board.

Dr Longstaff: The third thing is that, as the committee knows, The Ethics Centre has not made a submission to this inquiry, and so I am appearing as a result of a request from the committee. So, if I seem to be thinking aloud in responding to your questions, that is because I will be thinking aloud.

CHAIR: Okay. Do you have an opening statement?

Dr Longstaff: No.

CHAIR: You did make a submission to the whistleblower inquiry though, didn't you?

Dr Longstaff: I did indeed.

CHAIR: Yes. Do you think we might have got our wires crossed?

Dr Longstaff: I do not know.

Senator O'NEILL: One of your colleagues gave evidence to our Melbourne hearing into whistleblowing. Because of the ethical nature of some of these considerations, we thought it might be helpful to also hear from you, Dr Longstaff, about the ethics of some of the issues that we are confronting. Were you able to hear the evidence from the previous witness in today's hearing?

Dr Longstaff: I did, yes—well, not all of it, just part of it.

Senator O'NEILL: Just to kick us off, can I invite you to reflect on what you heard—in terms of the ethical tussle between the privacy of people's health information and the need for the insurance industry to remain viable and to serve its purpose in our society? That is the point. I suppose we have to find some middle way. If you can respond to that in your opening remarks, that would be helpful.

Dr Longstaff: Okay, thank you, Senator. I was that person who gave evidence in Melbourne last time.

Senator O'NEILL: Oh, sorry.

CHAIR: We just did not recognise you.

Senator O'NEILL: You look like the invisible man, today, Dr Longstaff.

Dr Longstaff: Going back to first principles in relation to insurance and genetic testing, one of the original purposes for which insurance was developed was to deal with the uncertainty of the human condition. People would pool their resources in those conditions of uncertainty with an expectation that, if some adverse event occurred to them, then the pool of others would draw upon those resources to try to make provision for it, whether it was in terms of funeral insurance or health insurance or ultimately life insurance. Although the quality of genetics is such as the moment that there are very few conditions where one can be absolutely certain about the likely consequences of a particular discovery around an individual's genome, nonetheless, the tendency to try to get greater and greater precision and predictability in relation to the likely fate of an individual by the insurance industry starts at some point to cut across the very purpose for which insurance was originally developed, so much so that, if you could get to a point where you had absolute certainty about the particular fate of a particular individual based on their genome, there would not longer be insurance in the sense in which we understand it. It would simply be a risk-weighted calculation that the individual and the company would take. All of the communal benefits that come from insurance would progressively be lost. We are a long way from that, but that is one of the first concerns at the level of basic principle I might have about this.

The second issue is to do with some of the matters where I heard people giving evidence a moment before. We certainly do not want, as a matter of the public interest, to find people withdrawing from the prospect of engaging in research, which should be for the benefit of the community as a whole, for fear that they will be apprised of

information, which they would then have to disclose to an insurer, to their detriment. If we are focusing here in those aspects of your inquiry around insurance, particularly life insurance, I would be counselling caution about allowing the insurance industry to push too far in terms of either demanding testing or using the results of testing in order to risk-weight the individual. The balance is probably about right now, but it could become adversely set in due course.

Senator O'NEILL: Thank you. If I could go to some more general questions. One of the recommendations that has been made to us by the Australian Genetic Non-Discrimination Working Group was that the government should basically immediately enact a moratorium on the use of the genetic data by life insurers and move to have a no-use restriction embedded in legislation—

CHAIR: And no access.

Senator O'NEILL: and no access. What are your views about that, Dr Longstaff?

Dr Longstaff: I would support that.

Senator O'NEILL: Excellent, okay. One of the things, I think, Senator Ketter explored—and he might want to jump in here—was some of the potential conflict of interest in the sector with regard to professional practice by independent medical examiners and the service model that they provide to, I think, mainly government agencies, who were at the top of the list, in whose pay they were employed and in whose interest it was to decrease the amount of claim et cetera. How are these conflicts of interest best managed in your view?

Dr Longstaff: There are two dimensions to this. The first is that, although many members of professions operate independently of any other organisation like sole traders, there are many lawyers who work for corporations, engineers who work for corporations, and others who develop a thick enough ethical skin, if you like, around them so that they are able both to operate in a self-interest corporate environment while also maintaining very high ethical standards. We think that is an unexceptional achievement for people to be able to do that. One response to this conflict is to say to what degree can we be confident that these examiners have very thick ethical skins such that they never ever allow the interests of those who are paying their fee to affect their capacity to provide disinterested judgement.

There may be some circumstances where we think that this unlikely to be a sufficient protection, because in these cases it is ultimately the interests of an individual, who is usually relatively powerless in relation to the entity that is trying to make the assessment. In most cases we would want to try to reinforce at a structural level the degree of independence of those people who have to exercise judgement. How one does that in practice I do not know. I do not know whether or not there is some kind of statutory reserve that pays the fees for such people or whether there is some kind of independent third party who acts as an intermediary between those seeking the information and the doctor or other person making the assessment. But, if we do not think that that ethical skin is going to be sufficient, then we need to put in place that mechanism.

Senator O'NEILL: Thank you. That was embedded in some of the questions we did ask earlier today of the service provider of independent medical examiners. It was pretty clear that, currently, there are no formal oversight bodies that look after independent medical examiners to see how thick that ethical skin is, as you described it.

I do not have any further, detailed questions. I know it was a short appearance, Dr Longstaff, but it was extremely helpful. Can I indicate that I might have some more questions on notice after we review the day, and we might have some sticky questions that we would like to send to you for your formal response.

Dr Longstaff: Okay, quality over quantity.

Senator O'NEILL: I think that is absolutely the case. Thank you.

Dr Longstaff: I will try to come in, yet, another guise next time I appear.

CHAIR: Dr Longstaff, thank you for taking the time to take our call and, hopefully, we have given you enough time to catch your plane.

Dr Longstaff: Absolutely.

CHAIR: Any questions that were taken on notice should be provided by 9 June 2017. We thank you for appearing at the hearing today via teleconference and for your evidence.

Dr Longstaff: Thank you very much, goodbye.

CHAIR: That concludes today's proceedings.

Committee adjourned at 15:42
